# The evolution of vertebrate somatostatin receptors and their gene regions involves extensive chromosomal rearrangements

**DOI:** 10.1186/1471-2148-12-231

**Published:** 2012-11-29

**Authors:** Daniel Ocampo Daza, Görel Sundström, Christina A Bergqvist, Dan Larhammar

**Affiliations:** 1 Department of Neuroscience, Science for Life Laboratory, Uppsala Universitet, Box 593, SE-75124, Uppsala, Sweden; 2 Present address: Department of Medical Biochemistry and Microbiology, Science for Life Laboratory, Uppsala Universitet, Box 582, SE-75123, Uppsala, Sweden

**Keywords:** Somatostatin receptors, Whole genome duplications, Chromosome rearrangements

## Abstract

**Background:**

Somatostatin and its related neuroendocrine peptides have a wide variety of physiological functions that are mediated by five somatostatin receptors with gene names *SSTR1-5* in mammals. To resolve their evolution in vertebrates we have investigated the SSTR genes and a large number of adjacent gene families by phylogeny and conserved synteny analyses in a broad range of vertebrate species.

**Results:**

We find that the SSTRs form two families that belong to distinct paralogons. We observe not only chromosomal similarities reflecting the paralogy relationships between the SSTR-bearing chromosome regions, but also extensive rearrangements between these regions in teleost fish genomes, including fusions and translocations followed by reshuffling through intrachromosomal rearrangements. These events obscure the paralogy relationships but are still tractable thanks to the many genomes now available. We have identified a previously unrecognized SSTR subtype, *SSTR6*, previously misidentified as either *SSTR1* or *SSTR4.*

**Conclusions:**

Two ancestral SSTR-bearing chromosome regions were duplicated in the two basal vertebrate tetraploidizations (2R). One of these ancestral SSTR genes generated *SSTR2, -3* and -*5*, the other gave rise to *SSTR1, -4* and -*6*. Subsequently *SSTR6* was lost in tetrapods and *SSTR4* in teleosts. Our study shows that extensive chromosomal rearrangements have taken place between related chromosome regions in teleosts, but that these events can be resolved by investigating several distantly related species.

## Background

The availability of a large variety of annotated and assembled vertebrate genome sequences has made it possible to address specific evolutionary questions on a genome-wide scale. This includes both large-scale analyses of genome evolution [1-5] and targeted comparative evolutionary studies of specific gene families. The Ensembl genome database (http://www.ensembl.org) includes genomes for representatives of most vertebrate classes, as well as suitable out-groups for the study of vertebrate evolution [6]. The recent addition of the genomes of the Comoran coelacanth *Latimeria chalumnae* and the spotted gar *Lepisosteus oculatus* complements the previous set of species with pivotal out-groups for tetrapods and teleost fishes, respectively. They are especially important for studies of genomic events that have taken place in either teleost or tetrapod evolution, as is the case for the chromosomal regions described in the present study.

The basal vertebrate whole genome duplications (2R) [1,3,4] and subsequently the teleost-specific genome duplication (3R) [2,7] have expanded numerous endocrine and neuronal gene families, see for example references [8-15]. Here we have subjected the chromosomal regions harboring the somatostatin receptor family genes to a detailed analysis by collecting sequences from a broad range of vertebrate genomes, including several teleost fishes as well as the spotted gar and the coelacanth.

Somatostatin, the short peptide responsible for inhibition of growth hormone release, was sequenced from sheep hypothalamus in 1973 [16] and its discovery was one of the achievements highlighted by the 1977 Nobel Prize in physiology or medicine. Subsequently this 14-amino-acid peptide was sequenced in numerous other vertebrate species and was found to be highly conserved during evolution. Somatostatin is widely distributed and serves both as a neuroendocrine peptide regulating the pituitary, a neuropeptide acting on other neurons, and as an endocrine peptide. In accordance with this, somatostatin has been reported to have many physiological effects [17]. A somatostatin-related peptide was discovered in mouse and human and was named cortistatin or somatostatin-2 [18]. It is now known to be present throughout the tetrapods. In teleost fishes additional somatostatin-like peptides exist named somatostatin 3–6, each encoded by a separate gene [19]. All of these duplicates may have arisen through chromosome duplications in 2R and 3R [19,20].

After the first identification of binding sites for somatostatin, evidence began to accumulate for more than one receptor subtype. The cloning era of G-protein-coupled receptors led to the discovery of five somatostatin receptor subtypes in mammals, named *SSTR1* through *5*[21]. The conserved structure of somatostatin receptor genes consists of a single exon encoding protein products of approximately 360 to 420 amino acids. The somatostatin receptors have been classified into two subfamilies based upon their degree of sequence identity: The human *SSTR1* and *SSTR4* amino acid sequences share 70% sequence identity in the region spanning TM1 to TM7 (including the loops), while *SSTR2*, *-3* and *-5* share 56-66% amino acid sequence identity to each other. All five receptor subtypes inhibit adenylyl cyclases [22] and they can also trigger other second messenger pathways to various extents.

Homologs of the mammalian somatostatin receptors have been described in several teleost fishes, see *Nelson & Sheridan (2005)*[23] for review. However, no *SSTR4* subtype has yet been described in a teleost fish. The known SSTR repertoire in chicken is the same as in mammals and several of the receptors have been studied functionally [24,25]. It was proposed several years ago that the SSTR family expanded in 2R [21] although it was not clear how the appearance of the five members correlated with the two genome doublings. A more recent phylogenetic analysis [26] presented a tree that was unresolved both with respect to species taxonomy and somatostatin receptor subtypes. Other investigators have proposed that the SSTRs arose from a series of duplications throughout vertebrate evolution [27,28].

Our analyses allow us to conclude that the chromosome duplications in early vertebrate evolution (2R), and in the teleost tetraploidization (3R), can explain the known repertoire of vertebrate somatostatin receptors. Furthermore, we have discovered that one of the teleost receptors represents a sixth ancestral vertebrate subtype that we have called *SSTR6*, which is still present in some teleost fishes, the spotted gar and the coelacanth, but has been lost in tetrapods. Thus, the somatostatin receptor system obtained its present complexity already in the early stages of vertebrate evolution. By centering our analyses around the SSTR genes we could also disentangle complex rearrangements in the SSTR-bearing chromosome regions in teleost fish genomes. This has implications for analyses of conserved synteny and the assignment of orthology for genes located in these regions.

## Results

### Phylogenetic analysis of the SSTR gene family; identification of a sixth SSTR subtype

Somatostatin receptor amino acid sequences were collected from genome databases for several species representing most of the vertebrate classes: In addition to tetrapod and teleost fish genomes, the genomes of the Comoran coelacanth (*Latimeria chalumnae*) and the spotted gar (*Lepisosteus oculatus*) were investigated in order to provide relative dating points earlier in the evolution of lobe-finned fishes (Sarcopterygii) and ray-finned fishes (Actinopterygii), respectively. The identified amino acid sequences include predictions from several previously unknown SSTR sequences. These results are summarized in Table 1, and detailed descriptions of the identified sequences are included as Supplemental note 1 (see Additional file 1).

**Table 1:** Summary of the identified somatostatin receptor sequences analyzed in this study

	**Genus and species (genome assembly version)**	**Assigned sequence names**	**Chromosome/linkage group /genomic scaffold locations**
Mammals	*Homo sapiens*	Human *SSTR1*	14: 38.68 Mb
	(GRCh37)	Human *SSTR2*	17: 71.16 Mb
		Human *SSTR3*	22: 37.60 Mb
		Human *SSTR4*	20: 23.02 Mb
		Human *SSTR5*	16: 1.12 Mb
	*Mus musculus*	Mouse *SSTR1*	12: 59.31 Mb
	(NCBIM37)	Mouse *SSTR2*	11: 113.48 Mb
		Mouse *SSTR3*	15: 78.37 Mb
		Mouse *SSTR4*	2: 148.22 Mb
		Mouse *SSTR5*	17: 25.63 Mb
	*Canis familiaris*	Dog *SSTR1*	8: 19.58 Mb
	(BROAD2)	Dog *SSTR2*	9: 10.00 Mb
		Dog *SSTR3*	10: 30.40 Mb
		Dog *SSTR5*	6: 42.65 Mb
	*Monodelphis domestica*	Opossum *SSTR1*	1: 278.65 Mb
	(BROADO5)	Opossum *SSTR2*	2: 217.49 Mb
		Opossum *SSTR3*	8: 91.98 Mb
		Opossum *SSTR4*	1: 598.18 Mb
		Opossum *SSTR5*	6: 153.47 Kb
Birds	*Gallus gallus*	Chicken *SSTR1*	5: 39.75 Mb
	(WASHUC2)	Chicken *SSTR2*	18: 9.00 Mb
		Chicken *SSTR3*	1: 53.39 Mb
		Chicken *SSTR4*	3: 3.27 Mb
		Chicken *SSTR5*	14: 5.64 Mb
Reptiles	*Anolis carolinensis*	Anole lizard *SSTR1*	a
	(AnoCar2.0)	Anole lizard *SSTR2*	2: 96.75 Mb
		Anole lizard *SSTR3*	5: 22.84 Mb
		Anole lizard *SSTR5*	GL343263.1: 1.76 Mb
Amphibians	*Xenopus tropicalis*	Frog *SSTR1*	GL172781.1: 1.07 Mb
	(JGI_4.2)	Frog *SSTR2*	GL172812.1: 1.79 Mb
		Frog *SSTR3*	GL172724.1: 1.41 Mb
		Frog *SSTR4*	GL172884.1: 512.43 Kb
		Frog *SSTR5*	GL172659.1: 446.17 Kb
Coelacanth	*Latimeria chalumnae*	Coelacanth *SSTR1*	JH126598.1: 0.53 Mb
	(LatCha1)	Coelacanth *SSTR2*	JH126581.1: 3.45 Mb
		Coelacanth *SSTR3*	JH129649.1: 0.21 Mb
		Coelacanth *SSTR4*	JH126648.1: 2.61 Mb
		Coelacanth *SSTR5*	JH129247.1: 0.21 Mb
		Coelacanth *SSTR6*	JH127490.1: 0.26 Mb
		Coelacanth *SSTRX*	JH126581.1: 3.47 Mb
Spotted gar	*Lepisosteus oculatus*	Spotted gar *SSTR1*	LG7: 4.44 Mb
	(LepOcu1)	Spotted gar *SSTR2*	LG10: 34.84 Mb
		Spotted gar *SSTR3*	LG12: 34.19 Mb
		Spotted gar *SSTR5*	LG13: 4.69 Mb
		Spotted gar *SSTR6*	LG28: 1.08 Mb
Teleost fish	*Danio rerio*	Zebrafish *SSTR1*	17: 10.35 Mb
	(Zv9)	Zebrafish *SSTR2a*	3: 63.08 Mb
		Zebrafish *SSTR2b*	12: 1.73 Mb
		Zebrafish *SSTR3a*	3: 29.75 Mb
		Zebrafish *SSTR3b*	Scaffold Zv9_NA631: 3.42 Kb
		Zebrafish *SSTR5a*	24: 16.78 Mb
		Zebrafish *SSTR5b*	1: 55.01 Mb
		Zebrafish *SSTR6*	7: 19.63 Mb
	*Gasterosteus aculeatus*	Stickleback *SSTR2a*	groupXI: 9.50 Mb
	(BROADS1)	Stickleback *SSTR2b*	groupV: 6.81 Mb
		Stickleback *SSTR3a*	groupXI: 15.59 Mb
		Stickleback *SSTR5a*	groupXI: 11.72 Mb
		Stickleback *SSTR5b*	groupIX: 14.95 Mb
		Stickleback *SSTR6*	scaffold_47: 436.21 Kb
	*Oryzias latipes*	Medaka *SSTR2a*	8: 10.93 Mb
	(MEDAKA1)	Medaka *SSTR2b*	scaffold5841: 160 bp
		Medaka *SSTR3a*	8: 2.80 Mb
		Medaka *SSTR3b*	1: 29.10 Mb
		Medaka *SSTR5a*	8: 13.75 Mb
	*Tetraodon nigroviridis*	Green puffer *SSTR2a*	3: 10.44 Mb
	(TETRAODON8)	Green puffer *SSTR2b*	2: 4.83 Mb
		Green puffer *SSTR3a*	3: 15.06 Mb
		Green puffer *SSTR3b*	18: 10.39 Mb
		Green puffer *SSTR3c*	Un_random: 59.49 Mb
		Green puffer *SSTR5b*	18: 2.40 Mb
	*Takifugu rubripes*	Fugu *SSTR2a*	scaffold_115: 411.36 Kb
	(FUGU4)	Fugu *SSTR2b*	scaffold_3: 3.77 Kb
		Fugu *SSTR3a*	scaffold_359: 200.56 Kb
		Fugu *SSTR3b*	scaffold_407: 33.36 Kb
		Fugu *SSTR5b*	scaffold_189: 267.65 Kb
		Fugu *SSTR6*	scaffold_164: 38.74 Kb
Invertebrates	*Drosophila melanogaster*	Fruit fly *Drostar1*	3L: 18.55 Mb
	(BDGP5)	Fruit fly *Drostar2*	3L: 18.48 Mb

^a^ The Anole lizard *SSTR1* sequence could not be identified in the most updated assembly (AnoCar2.0), however it is located on genomic scaffold_0 at 284.26 Kb in the previous assembly (AnoCar1.0, Ensembl database version 60).

**Figure 1:**
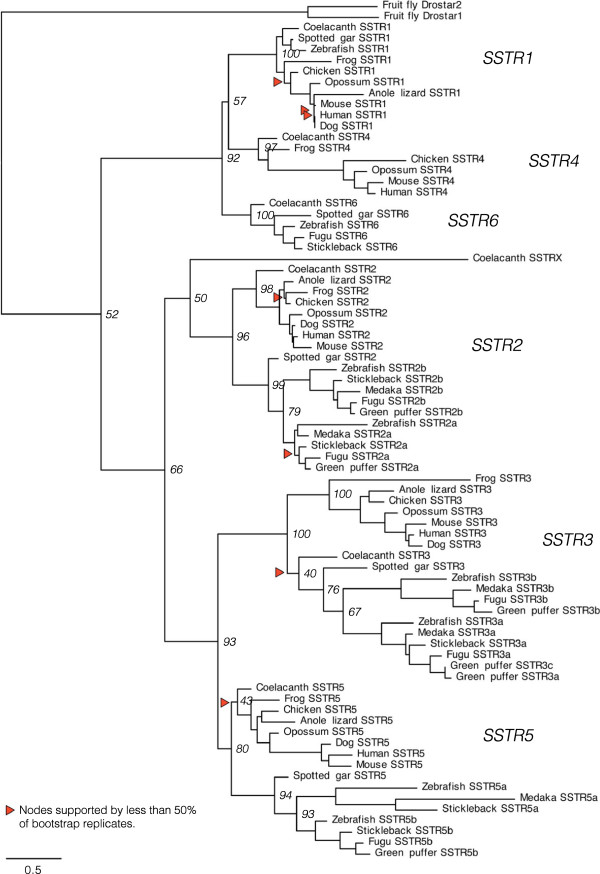
**Phylogenetic maximum likelihood tree of the somatostatin receptor gene family.** The topology is supported by a non-parametric bootstrap test with 100 replicates as well as an SH-like approximate likelihood ratio test (aLRT). The tree is rooted with the human kisspeptin receptor 1 sequence (not shown). Branch support (bootstrap replicates) for deep divergences is shown at the nodes. All branch support values are shown in Figure S1 (bootstrap replicates) and Figure S2 (aLRT) (see Additional file 2). The phylogenetic tree shows six well-supported subtype clusters, with the somatostatin receptor subtypes *SSTR2, -3* and -*5* forming one ancestral branch and the *SSTR1, -4* and -*6* receptor subtypes forming one ancestral branch. This phylogenetic analysis supports the emergence of all six subtypes early in vertebrate evolution, with the subsequent loss of *SSTR4* in ray-finned fishes, before the divergence of the spotted gar and teleost lineages, and of *SSTR6* in the tetrapod lineage. All six subtypes could be identified in the coelacanth genome. A seventh *SSTR2*-like sequence, called *SSTRX* in the tree, could also be identified on the same genomic scaffold in the coelacanth genome (see Additional file 1, Supplemental note 1). There are well-supported teleost-specific duplicate branches of *SSTR2*, *-3* and -*5*, although all could not be identified in all teleost genomes. These duplicates have been named *a* and *b* based on the phylogenetic analysis. There is a third *SSTR3* sequence in the green puffer, called *SSTR3c* in the tree.

The SSTR amino acid sequences identified in the genome databases were used to create an alignment for phylogenetic analyses in order to determine the identity of previously unknown SSTR sequences and study the evolution of this gene family. Using the human kisspeptin-1 receptor as out-group, the resulting phylogenetic maximum likelihood (PhyML) tree in Figure 1 shows that the vertebrate SSTR family consists of six subtype clusters representing the five known SSTR subtypes *SSTR1* through *SSTR5*, as well as a previously unrecognized sixth subtype. We have named these sequences *SSTR6* in our studies*.* In agreement with previous analyses of fewer sequences [21,23,27], the tree has two well-defined ancestral branches; one including *SSTR2*, *-3* and -*5*, and one containing the *SSTR1* and -*4* as well as the *SSTR6* subtype. Both branches are well-supported, and the separate SSTR subtypes form well-supported clusters within each branch, using both bootstrapping and SH-like approximate likelihood ratio statistics (see Additional file 2, Figures S1 and S2). Some subtypes are missing from some species’ genome databases (see Additional file 1, Supplemental note 1). Notably, sequences of the sixth subtype, *SSTR6,* could not be identified in any of the investigated tetrapod sequences, and *SSTR4* sequences could not be identified in teleost fishes or in the spotted gar. All six SSTR subtypes are represented in the coelacanth, demonstrating that the absence of *SSTR4* genes in the spotted gar and teleost fishes, and of *SSTR6* genes in tetrapods likely resulted from secondary gene losses. In teleost fishes, an *SSTR1* sequence could only be identified in the zebrafish genome.

There are teleost specific duplicates of *SSTR2, -3* and -*5* forming well-supported *a-* and *b-*clusters within their respective subtypes. In the spotted gar genome only single copies of the *SSTR2, -3* and -*5* sequences were found, and these branch basal to the respective teleost-specific *a-* and *b*-duplicate clusters, which strongly supports the duplication of *SSTR2, -3* and -*5* early in the teleost lineage. Taken together this means that some teleost species may have up to eight different SSTR family members, including an SSTR subtype that has not been previously described. In our analyses, the zebrafish genome has this repertoire of receptors: *SSTR1, -2a, -2b, -3a, -3b, -5a, -5b* and -*6*.

The known *Drosophila* allatostatin C receptor 1 and 2 sequences called *Drostar1* and *Drostar2* were included in the phylogenetic analyses due to their close sequence and functional similarity with the mammalian somatostatin receptor [29]. These sequences cluster together basal to the vertebrate SSTR sequences and most probably represent an independent duplication event. It was not possible to identify true SSTR orthologs in the tunicates *Ciona intestinalis* and *Ciona savignyi*, or in the Florida lancelet (amphioxus) *Branchiostoma floridae.*

### Syntenic gene families

In addition to making a phylogenetic tree of somatostatin receptors in vertebrates, our aim was to determine whether the SSTR genes were duplicated in the chromosome doublings in 2R. To test this hypothesis, syntenic (neighboring) gene families in the SSTR gene-bearing chromosome regions were analyzed with respect to their phylogenies, using both neighbor joining (NJ) and PhyML methods, and the chromosomal locations of the member genes (see Methods below). In total, 47 syntenic gene families were analyzed. Our results of the conserved synteny analyses are presented as tables comparing the chromosomal locations of all the identified syntenic family member genes in the genomes of human, chicken, zebrafish, stickleback and medaka. Due to size restrictions, the tables have been included as additional data files (see Additional files 3 and 4). The phylogenetic trees of all the neighboring gene families have also been included as additional files (see Additional files 5 and 6). These tables and phylogenetic trees are the bases for our description of the results below.

### Conserved synteny analysis of the *SSTR1*, *-4* and *-6* chromosome regions

**Figure 2:**
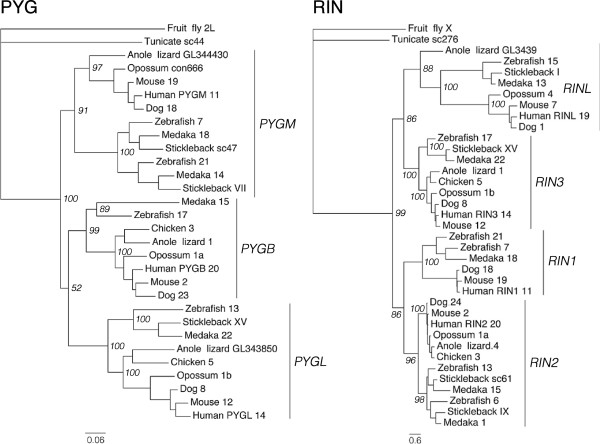
**Phylogenetic maximum likelihood trees of the PYG and RIN gene families.** The glycogen phosphorylase (PYG) and Ras and Rab interactor (RIN) gene families are neighboring families of the *SSTR1, -4* and -*6* chromosomal regions. Monophyletic subtype clusters including both tetrapod and teleost sequences are indicated by bars to the right. Chromosomal or genomic scaffold assignments of the family members are indicated next to species names. Lowercase a and b are used to distinguish sequences located on the same chromosomes. Branch support values (bootstrap replicates) for deep divergences are shown at the nodes. The trees were rooted with the identified fruit fly sequences. All neighboring gene family trees for the *SSTR1, -4* and -*6*-bearing regions, including NJ analyses and all branch support values, are shown in Figures S4-S20 (see Additional file 5).

The chromosomal locations of the SSTR genes as well as the early divergence of two ancestral SSTR branches in the phylogenetic tree suggested that the *SSTR1, -4* and -*6* genes derive from one ancestral *SSTR* gene, and the *SSTR2, -3* and -*5* genes from a separate ancestral SSTR gene, and that these two ancestral genes were located in distinct paralogons (related chromosome groups). Therefore two separate analyses of conserved synteny were done. To investigate whether the *SSTR1, -4* and *-6* genes arose by duplications of a single ancestral gene in 2R we have carried out phylogenetic analyses of 17 syntenic gene families and the chromosomal locations of all neighboring family members were noted and compared between species (see Additional file 3). In summary, all but two of the 17 identified syntenic gene families in the *SSTR1, -4* and -*6* chromosome blocks (Table 2) have phylogenetic trees that either support or are consistent with duplications early in vertebrate evolution (see Additional file 5). These gene families have tree topologies with subtype clusters diverging in the same time window as 2R, i.e., after the divergence of invertebrate chordates and vertebrates but before the divergence of lobe-finned fishes (including tetrapods) and ray-finned fishes (including teleosts). The PhyML topologies of the RIN and PYG gene families are shown as examples in Figure 2. Several gene families also have teleost-specific duplicate clusters, supporting subsequent duplications in 3R, see for example *PYGM* and *RIN2* clusters in Figure 2 as well as the teleost *FLRT1* orthologs (see Additional file 2, Figure S4). Some of the neighboring families have inconsistencies between the NJ and PhyML trees (see Additional file 1, Supplemental note 2), however they were considered supportive if they showed the topology described above for at least one of the methods.

**Table 2:** **Neighboring gene families analyzed for conserved synteny in the *SSTR1, -4 *and -*6*-bearing chromosome blocks**

**Symbol**^**a**^	**Description**^**a**^	**Root (if other than *D. melanogaster)***
ABHD12	*Abhydrolase domain containing 12*	
CFL	*Cofilin and destrin (actin depolymerizing factor)*	
FLRT	*Fibronectin leucine rich transmembrane protein*	*C. savignyi*
FOXA	*Forkhead box A*	
ISM	*Isthmin homolog*	*C. intestinalis*
JAG	*Jagged*	
NIN	*Ninein (GSK3B interacting protein)*	
NKX2	*NK2 homeobox 1 and 4*	
PAX	*Paired box 1 and 9*	
PYG	*Glycogen phosphorylase; brain, liver and muscle variants*	
RALGAPA	*Ral GTPase activating protein, alpha subunit*	
RIN	*Ras and Rab interactor*	
SEC23	*Sec23 homologs A and B*	
SLC24A	*Solute carrier family 24 members 3 and 4*	*B. floridae*
SNX	*Sorting nexin 5, 6 and 32*	
SPTLC	*Serine palmitoyltransferase, long chain base subunit 2 and 3*	
VSX	*Visual system homeobox*	*C. elegans*

^a^ Gene family names and descriptions are based on approved HUGO Gene Nomenclature Committee (HGNC) gene symbols and descriptions, or known aliases from the NCBI Entrez Gene database. Where not all known protein subtypes/isoforms are part of the gene family, the included subtypes are specified.

**Table 3:** **Neighboring gene families analyzed for conserved synteny in the *SSTR2, -3 *and -*5*-bearing chromosome blocks**

**Symbol**^**a**^	**Description**^**a**^	**Root (if other than *D. melanogaster)***
ADAP	*ArfGAP with dual PH domains*	
ATP2A	*ATPase, Ca++ transporting, cardiac muscle, fast twitch*	
C1QTNF	*C1q and tumor necrosis factor related protein*	*B. floridae*
CABP	*Calcium binding protein 1, 3, 4 and 5*	
CACNA1	*Calcium channel, voltage dependent, T type alpha subunit*	
CREBBP	*CREB binding protein*	
CYTH	*Cytohesin*	
FAM20	*Family with sequence similarity 20*	
FNG	*Fringe homolog*^*b*^	
FSCN	*Fascin homolog 1 and 2, actin-bundling protein*	
GGA	*Golgi-associated, gamma adapting ear containing, ARF-binding protein*	
GLPR	*Glucagon, glucagon-like and gastric inhibitory polypeptide receptors*	*C. intestinalis*
GRIN2	*Glutamate receptor, ionotropic, N-methyl D-aspartate 2*	
KCNJ	*Potassium inwardly-rectifying channel, subfamily J member 2, 4, 12 and 14*	*C. intestinalis*
KCTD	*Potassium channel tetramerisation domain containing 2, 5 and 17*	
METRN	*Meteorin, glial cell differentiation regulator*	*B. floridae*
NDE	*nudE nuclear distribution gene E homolog*	
RAB11FIP	*RAB11 family interacting protein 3 and 4 (class II)*	
RADIL	*Ras association and DIL domains/Ras interacting protein*	*B. floridae*
RHBDF	*Rhomboid 5 homolog*	
RHOT	*Ras homolog gene family, member T1 and T2*	
RPH3A	*Rabphilin 3A homolog/double C2-like domains, alpha*	
SDK	*Sidekick cell adhesion molecule*	*C. elegans*
SOX	*Sex-determining region Y-box 8, 9 and 10*	
TEX2	*Testis expressed 2*	
TNRC6	*Trinucleotide repeat containing 6*	
TOM1	*Target of myb1*	
TTYH	*Tweety homolog*	
USP	*Ubiquitin specific peptidase 31 and 43*	
WFIKKN	*WAP, follistatin/kazal, immunoglobulin, kunitz and netrin domain contaning*	*B. floridae*

^a^ Gene family names and descriptions follow the same system as Table 2.

^*b*^ Complete description: *Lunatic, manic and radical fringe homolog. O-fucosylpeptide 3-beta-N-acetylglucosaminyltransferase.*

The positional and phylogenetic data combined demonstrate the conserved synteny between the chromosome blocks containing *SSTR1, -4* and -*6* in the analyzed genomes. In the human genome, these correspond to well-defined regions on chromosomes 14 and 20, and to a lesser degree 11 and 19. Although no *SSTR6*-bearing chromosomal region was used in the selection of syntenic gene families, the ISM (Figure S8), PYG (Figure S13), RIN (Figure S15) and SLC24A (Figure S17) families have members neighboring the *SSTR6* genes in one or several of the teleost genomes (see Additional file 3). This dataset also shows that rearrangements between the homologous chromosome regions have been common in the teleost lineage. For example, genes located on both chromosomes 14 and 20 in the human genome have orthologs that are distributed primarily between chromosomes 13, 17 and 20 in the zebrafish genome in a way that suggests the substantive exchange of paralogs between these regions. This can be seen for the teleost orthologs of the *PYGL* and *PYGB* genes compared with the *RIN3* and *RIN2* orthologs (Figure 2). In the stickleback and medaka genomes there seems to have been fewer rearrangements: orthologs of genes located on human chromosome 20 are located on stickleback linkage group XV between approximately 3.75 and 4 Mb, and on medaka chromosome 22 between approximately 16 and 16.34 Mb, which suggests translocation of small chromosomal blocks (see Additional file 3).

### Conserved synteny analysis of the *SSTR2*, *-3* and *-5* chromosome regions

**Figure 3:**
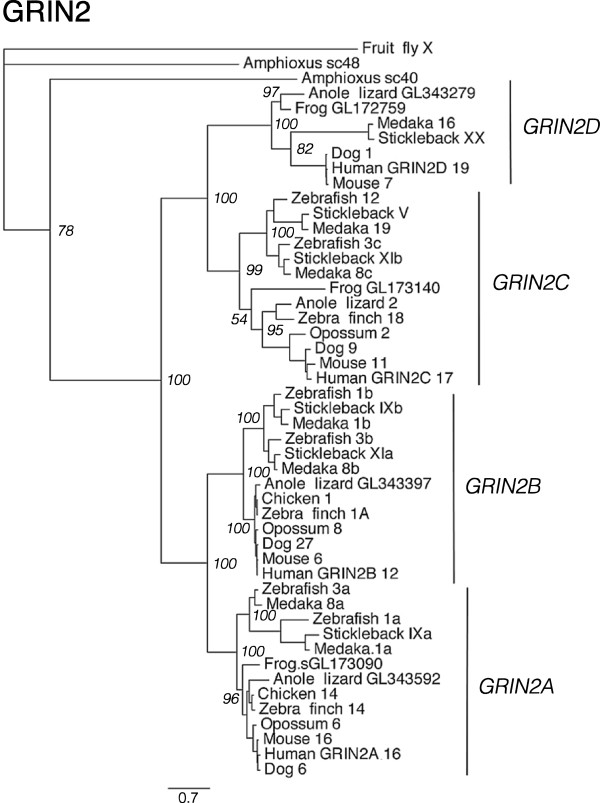
**Phylogenetic maximum likelihood tree of the GRIN2 gene family.** The ionotropic glutamate receptor 2 (GRIN2) gene family is a neighboring family of the *SSTR2, -3* and -*5* chromosomal regions. Phylogenetic methods, monophyletic clusters and leaf names as in Figure 2 .

**Figure 4:**
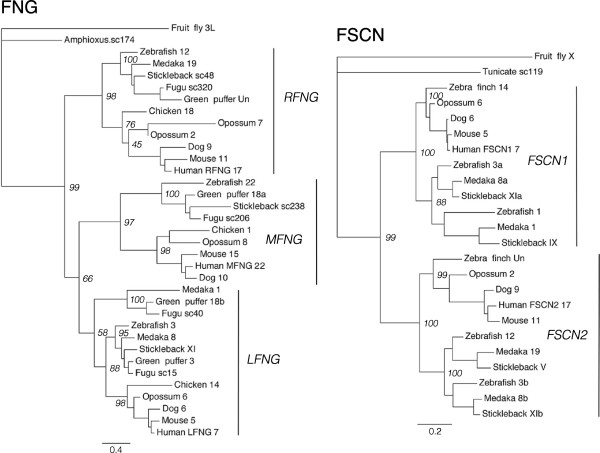
**Phylogenetic maximum likelihood trees of the FNG and FSCN gene families.** The fringe homolog (FNG) and fascin homolog 1 and 2 (FSCN) gene families are neighboring gene families of the *SSTR2, -3* and -*5* chromosomal regions. Phylogenetic methods, monophyletic clusters and leaf names as in Figure 2. All neighboring gene family trees for the *SSTR2, -3* and -*5-*bearing regions, including NJ analyses and all branch support values, are shown in Figure S21-S50 (see Additional file 6).

For the investigation of paralogy relationships between the chromosomal regions that harbor the *SSTR2, -3* and -*5* genes, 30 syntenic gene families were analyzed as described above for the *SSTR1, -4* and -*6*-bearing chromosome regions. To identify these gene families, the *SSTR2, -3* and -*5*-bearing chromosome regions in the chicken and stickleback genomes were analyzed for conserved synteny (see Methods below). Two separate starting points (chicken and stickleback) were used because the chromosomal locations of the SSTR genes in the teleost genomes, with *SSTR2*, *-3* and -*5* homologs located on the same chromosome, suggest a different expansion scenario than the tetrapod genomes, including chicken (Table 1). In this way we could collect a dataset of neighboring gene families without favoring one scenario over the other. In summary, 23 of the 30 syntenic gene families in the *SSTR2, -3* and -*5* chromosome blocks have tree topologies that support an expansion from one ancestral vertebrate gene through 2R (see Additional file 6, Figure S21–S50). Four are consistent with 2R, but show some inconsistencies between phylogenetic methods (see Additional file 1, Supplemental note 3) - ADAP (Figure S21), FAM20 (Figure S28), RPH3A and TOM1 (Figure S47) - while only two are considered inconclusive - CABP (Figure S24) and GGA (Figure S31) (see Additional file 6). Many of the analyzed families also show topologies that support the duplication of family members in 3R. The PhyML topologies of the GRIN2 family (Figure 3) and of the FNG and FSCN families (Figure 4) are shown as examples.

As described previously, the chromosomal locations of all neighboring family members were compared between species (see Additional file 4) and the phylogenetic tree topologies of the neighboring gene families were used to infer the paralogy and orthology relationships. The identified conserved synteny blocks correspond to regions of human chromosomes 7, 16, 17, 19 and 22 in the human genome. This dataset shows that there have been extensive chromosome rearrangements between the paralogous chromosome regions in the teleost genomes, and to some extent in the human genome. For example, many gene families with members on chicken chromosome 14 have orthologs distributed between human chromosomes 16 and 7, and stickleback linkage groups V, IX and XI. In the zebrafish genome these same gene families have orthologs spread over more chromosomes: most are on chromosomes 3, 12, 1 and 24, but there are individual orthologs of two families on chromosome 22 and an unmapped genomic scaffold (see Additional file 4). Several of these rearrangements can be seen for members of the GRIN2 (Figure 3), FNG and FSCN (Figure 4) families, with teleost-specific duplicates in different subtype clusters co-located on the same chromosomes. The human *GRIN2B* sequence also seems to have translocated to chromosome 12. The *GRIN2A*, *-2B* and *-2C* clusters show well-supported teleost duplicate branches, supporting a duplication in 3R (Figure 3). In these branches we observe teleost genes located on the same chromosomes (for instance zebrafish *GRIN2A*, *-2B* and -*2C* orthologs, all on chromosome 3), likely due to the chromosomal rearrangements. The FSCN gene family has several teleost sequences located on the same chromosomes, for instance on zebrafish chromosome 3, medaka chromosome 8 and stickleback linkage group XI, and the FNG family has teleost duplicates in the *LFNG* cluster (Figure 4). However, the topology is not clear for the *LFNG* teleost duplicates. These rearrangements in the teleost lineage likely explain why the *SSTR2a, -3a* and *-5a* genes also are located in the same chromosomal regions in teleost genomes (Table 1), as will be discussed below.

A few gene families identified in the analysis of conserved synteny, namely ATP2A (Figure S22), CABP (Figure S24), GLPR (Figure S32) and RPH3A (Figure S42) (see Additional file 6), have individual paralogs on different chromosomes or genomic scaffolds, as described in Supplemental note 3 (see Additional file 1).

## Discussion

### Evolution of the SSTR family

**Figure 5:**
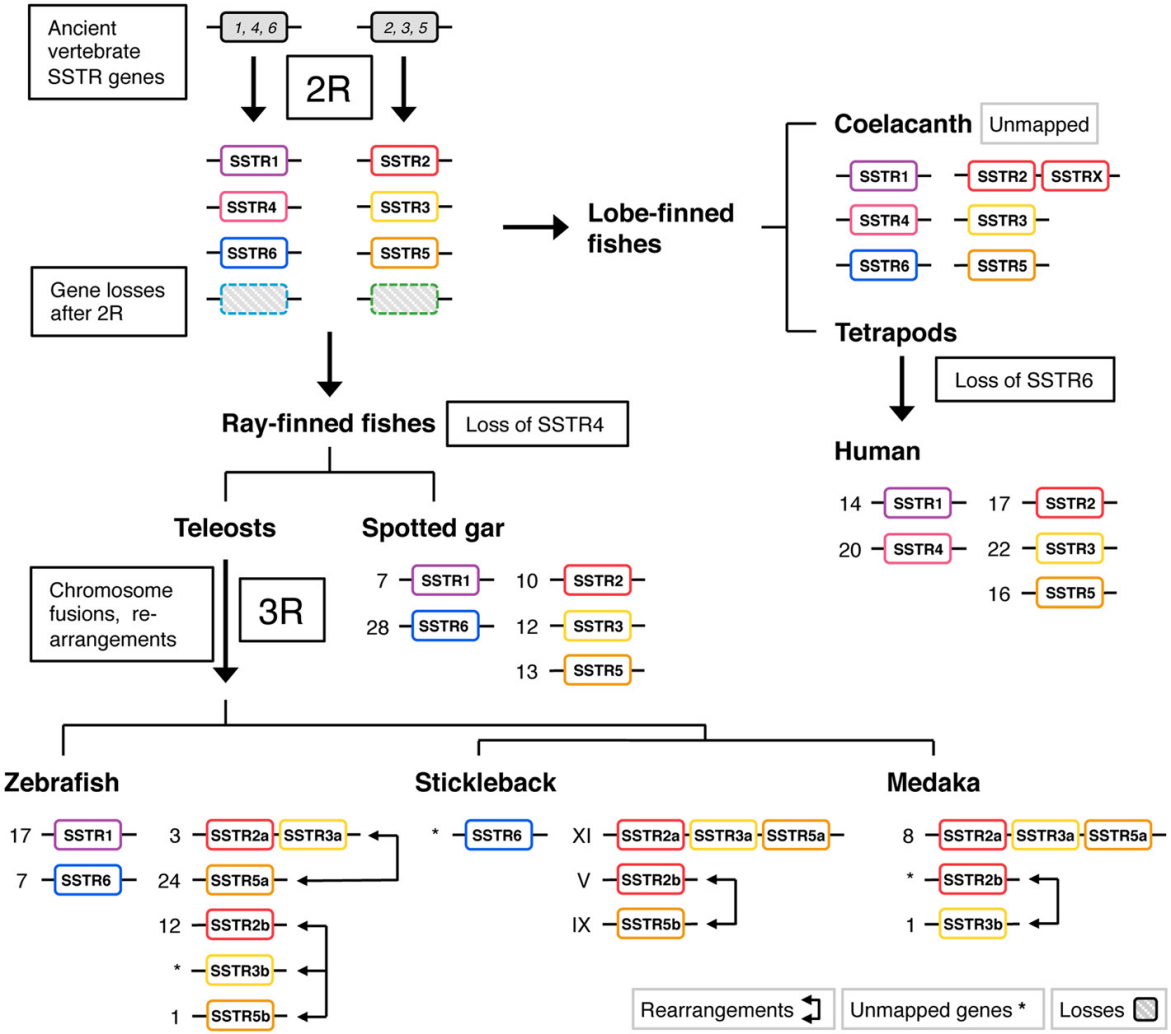
**Proposed somatostatin receptor evolutionary scheme.** Numbers denote chromosome or linkage group assignments of SSTR genes in mapped genomes. Some of the SSTR genes have not been mapped to chromosomes or linkage groups, which is indicated by asterisks. Evolutionary scheme: Two ancestral vertebrate SSTR genes located on two different chromosomes duplicated in 2R, generating the vertebrate SSTR gene repertoire of *SSTR1, -4* and -*6*, and *SSTR2, -3* and -*5* respectively. *SSTR6* was lost from the lobe-finned fish lineage some time after the divergence of the coelacanth, and *SSTR4* was lost from the ray-finned fish lineage some time before the divergence of the spotted gar. Following chromosome fusions, the ancestral teleost *SSTR2, -3* and -*5* genes duplicated in 3R, while only one gene for each of *SSTR1* or -*6* genes were conserved in some teleost lineages. Subsequent chromosome rearrangements in teleost evolution moved SSTR genes to different chromosomes. Data from neighboring genes families are consistent with these chromosome rearrangements. Not all SSTR subtype genes could be identified in some teleost genomes (Table 1). This could be either due to genuine gene losses, or perhaps due to the incomplete nature of these genome databases.

Our phylogenetic analyses of the SSTR gene family provide strong support for expansion and diversification in both the 2R and 3R events, giving rise to six different SSTR subtype genes early in vertebrate evolution and subsequently expanding the *SSTR2, -3* and -*5* branch in the teleost lineage. Our evolutionary scheme of the SSTR gene family expansion is presented in Figure 5. The sixth subtype, which we have called *SSTR6*, was previously unrecognized. We have identified it in the ray-finned fishes, including the spotted gar and the teleosts, as well as in the coelacanth, a member of the lobe-finned fish lineage. Thus, it was clearly present before the divergence of lobe-finned and ray-finned fishes. Its chromosomal position in the teleosts supports origin in 2R (*see below*). Conversely, the *SSTR4* gene was only identified in the lobe-finned fishes, including tetrapods and the coelacanth. These losses are likely the result of secondary and independent events: *SSTR6* from the lineage leading to tetrapods some time after the divergence of the coelacanth lineage, and *SSTR4* from the ray-finned fish before the divergence of the spotted gar and the lineage leading to teleosts (Figure 5). The topology of the *SSTR1, -4* and -*6* branch supports this scenario (Figure 1). All six SSTR subtypes that emerged early in vertebrate evolution are represented in the genome of the coelacanth. The additional seventh coelacanth sequence that we have called *SSTRX* is located on the same genomic scaffold with the same orientation as the *SSTR2* sequence (Table 1) and it clusters in the most basal position in the *SSTR2* cluster. This, together with its branch length in the tree, indicates that it is a lineage-specific duplicate of *SSTR2* with a higher evolutionary rate.

The somatostatin system has been reported to have arisen prior to the divergence of insects and vertebrates, i.e., before the protostome-deuterostome split. *Drosophila melanogaster* and other insects have a somatostatin-like 15-amino-acid peptide that has been named ASTC for allatostatin C [30]. Two ASTC receptors were identified in *D. melanogaster,* with closest relationship to human somatostatin and opioid receptors [29]. The receptors were named *Drostar1* and -*2* and seem to have arisen through a lineage-specific duplication in insects. We propose that two ancient SSTR genes were present before the emergence of vertebrates based on our comparative analyses. However, we were unable to identify any unambiguous SSTR family members in the genome databases of the amphioxus *Branchiostoma floridae*, and the tunicates *Ciona intestinalis* and *Ciona savignyi*. The latter are members of the urochordate lineage which constitutes the closest extant relatives of vertebrates [31,32]. A previous analysis of G-protein coupled receptor sequences in the Florida lancelet (*Branchiostoma floridae*) genome identified several lancelet-specific expansions of somatostatin-, galanin- and opioid receptor-like sequences, totaling 90 distinct sequences in this cluster [33]. Among these sequences, 18 cluster together with the human SSTR sequences, although the resolution of this phylogenetic analysis is very low. In any case, these lineage-specific duplications preclude the identification of true orthologs to the vertebrate somatostatin receptors, although there are several candidates. We have identified three putative somatostatin receptor sequences in the genome of the sea lamprey *Petromyzon marinus,* and their database identifiers are noted in Table S1 (see Additional file 7). However, due to the incomplete status of this genome assembly, and thus the lack of synteny data, we refrain from speculating about their orthology relationships.

Somatostatin receptors have been described for several teleost fish species in addition to the ones that we have studied. In each species usually one or a few sequences have been reported, except for goldfish, *Carassius auratus*, where eight sequences have been published [34-37]. Our additional tree presented in Figure S3 (see Additional file 2) confirms previous suggestions [23,28] that two of these correspond to *SSTR1* as a result of the goldfish-specific fourth tetraploidization (4R) that took place some 12–15 MYA [38,39]. Other goldfish sequences correspond to subtypes *SSTR2*, *SSTR3a* and *SSTR3b*. The three *SSTR5*-like sequences in goldfish were initially named *5a*, *5b*, and *5c*. The one named *5c* is orthologous to *5b* in our comparisons and the ones named *5a* and *5b* appear to be 4R duplicates of *5a* (see Additional file 2, Figure S3). The latter have accumulated as many as 66 amino acid differences in this short time period (resulting in 83% sequence identity), whereas the *SSTR1* 4R-generated duplicates differ at only 5 positions. In the orange-spotted grouper, *Epinephelus coioides*, four sequences have been reported [27] that we can now identify as *SSTR1*, *SSTR2b*, *SSTR3a*, and *SSTR5a* (see Additional file 2, Figure S3). The *SSTR3* sequence determined in the black ghost knifefish *Apteronotus albifrons*[40], an electric fish, is *SSTR3b*, and the two sequences from the cichlid *Astatotilapia burtoni*[41] are *SSTR2a* and *SSTR3a*. In the rainbow trout, *Oncorhynchus mykiss*, three sequences have been reported [42,43]. In our additional analysis the two sequences identified as *SSTR1a* and *-1b*[43,44] can be correctly identified as two copies of *SSTR6* likely resulting from the salmonid fourth tetraploidization (see Additional file 2, Figure S3).

### SSTR-bearing chromosome regions were duplicated in vertebrate whole genome duplications

**Figure 6:**
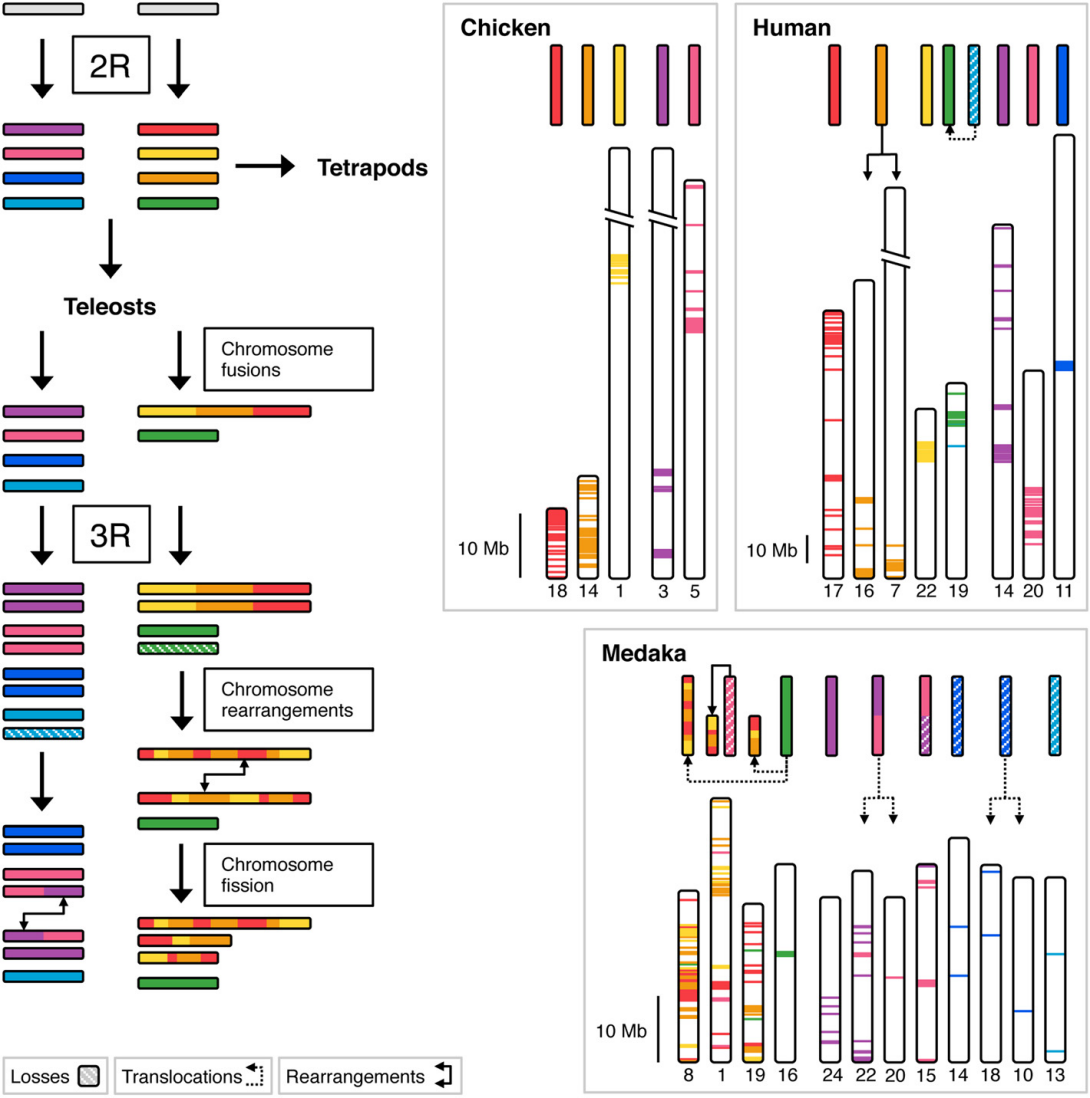
**Evolutionary scenario for the vertebrate SSTR gene-bearing chromosome regions.** Two ancient vertebrate chromosomes bearing one SSTR gene each duplicated in 2R, generating two vertebrate paralogons; one bearing *SSTR1, -4* and -*6* genes (purple, pink, blue and turquoise blocks) and one bearing *SSTR2, -3* and -*5* genes (red, yellow, orange and green blocks). After the divergence of lobe-finned fishes (including tetrapods) and ray-finned fishes (including teleosts), three of the 2R-generated blocks fused in the ray-finned fish lineage before 3R. Both paralogons duplicated in 3R, followed by rearrangements between paralogous chromosome blocks, obscuring the ancestral conserved synteny. One of the fused, duplicated and rearranged chromosome blocks split through a fission event. The paralogous chromosome regions have been reconstructed for the chicken, human and medaka genomes by mapping the identified paralogous gene families. The upper color blocks represent ancestral chromosome regions in each lineage. Dashed boxes represent losses of chromosome blocks. Chromosome rearrangements involving blocks of genes are represented by arrows, while smaller translocations of genes are represented by dashed arrows. The full datasets are presented in Tables S4 and S5 (see Additional files 3 and 4).

Two separate analyses of conserved synteny were carried out in order to test the hypothesis that each of the *SSTR1*, *-4*, *-6* and *SSTR2*, *-3*, *-5*-branches of the SSTR gene family was multiplied as a result of duplications of two distinct chromosome regions. In total we have compared the chromosomal locations of genes in 47 gene families located in SSTR-bearing chromosome regions. These positional data were combined with phylogenetic analyses of the gene families to infer the likely orthology and paralogy relationships within each family, as well as to determine the time window of the duplications and chromosome rearrangements. As a whole, these analyses show that the *SSTR1*, *-4* and -*6*-bearing chromosome regions on the one hand, and the *SSTR2*, *-3* and -*5*-regions on the other, belong to distinct paralogons that were formed by chromosome duplications during the same time period in early vertebrate evolution. Using relative dating in the phylogenetic analyses, as well as the species distribution of the genes, we can place the duplication events to the period after the divergence of invertebrate chordates and vertebrates, but before the divergence of lobe-finned fishes (including tetrapods) and ray-finned fishes (including teleosts). This means that the identified regions of paralogy likely resulted from duplications of ancestral chromosome regions in the same time window as the early vertebrate tetraploidizations. Thus, our analysis provides further support for 2R. Our analyses also indicate that these two paralogy regions duplicated further in the time-window of the teleost-specific whole genome duplication 3R, although for the SSTR gene family only duplicates of *SSTR2, -3* and -*5* were retained (Table 1). Based on the phylogenetic analyses of the SSTR family (Figure 1, Additional file 2), these duplicates have been named adding the letters *a* and *b* to the gene symbols. Our proposed evolutionary scenario for the evolution of the SSTR-bearing chromosome regions is presented in Figure 6.

The paralogous regions we have identified bearing *SSTR1, -4*, and *-6*-genes, and *SSTR2, -3* and -*5*-genes, and the time window for their origin, are consistent with previous large-scale genomic analyses. In the analysis of paralogous chromosome regions in the human genome compared to the *Branchiostoma floridae* genome [3] these regions (Figure 6) correspond to ancestral chordate linkage groups numbered 11 and 15 respectively, indicating an origin in 2R. A separate reconstruction of the vertebrate ancestral genome [4] also inferred that these regions originated from two separate ancestral chromosomes that quadrupled in 2R. In the latter analysis the *SSTR1, -4* and -*6*-bearing regions correspond to the ancestral linkage group called G and the *SSTR2, -3* and -*5*-bearing regions to ancestral linkage group called I. The analysis of the first medaka draft genome [5], as well as the aforementioned reconstruction of the ancestral vertebrate genome, support the conclusion that both paralogous regions duplicated further in 3R, but that there have been several major rearrangements that obscure the paralogy relationships. The medaka genome is an appropriate starting point for the discussion of chromosomal rearrangements in the teleost lineage since it seems to have preserved more of the ancestral teleost genome organization [5].

### Chromosomal rearrangements in teleost genomes

Initially, the locations of the *SSTR2a, -3a* and *-5a* duplicates in teleost genomes suggested that the expansion of the somatostatin receptor family might have partially occurred through other mechanisms than 2R. In the medaka and stickleback genomes all three paralogs are located within regions of approximately 11 Mb on chromosome 8 and 9 Mb on linkage group XI, respectively. In the zebrafish, *SSTR2a* and *-3a* are located approximately 33 Mb apart on chromosome 3 while *SSTR5a* is located on chromosome 24. In the green puffer *SSTR2a* and *-3a* are located approximately 5 Mb apart on chromosome 8 and additionally the *SSTR3b* and *-5b* genes are co-localized on chromosome 18 approximately 8 Mb apart (see Table 1 for locations). These arrangements would suggest that ancestral segmental duplications were involved. However, in all non-teleost genomes, notably that of the spotted gar, the *SSTR2, -3* and -*5* paralogs are located on different chromosomes or linkage groups (Table 1). To make sure that our analysis of conserved synteny did not favor the 2R scenario over the ancestral tandem duplication scenario, both the chicken and the stickleback genomes were used as starting points for the identification of neighboring families in the *SSTR2, -3* and -*5* paralogon. For the *SSTR1, -4* and -*6* paralogon we parted from the human and chicken genomes, since the locations of the SSTR genes did not indicate different expansion scenarios in tetrapods and teleosts. Based on the combined positional and phylogenetic data using tetrapods as well as teleosts we conclude that both of the SSTR-bearing paralogons have undergone a series of inter- and intra-chromosomal rearrangements in the teleost lineage that obscure the ancestral organization. To deduce these rearrangements we have compared lists of neighboring gene family members in the identified paralogous chromosome regions between the human, chicken, zebrafish, stickleback and medaka genomes. The results of this analysis are presented in Additional files 3 and 4 and our suggested scenario is summarized in Figure 6.

The analysis of conserved synteny for the *SSTR2, -3* and -5-paralogy regions shows that many of the gene families, not only SSTR, display the same paralog translocations between the homologous chromosome regions generated in 2R. Notable examples are the CYTH (Figure S27), FSCN (Figure S30), GGA (Figure S31), GRIN2 (Figure S33), KCNJ (Figure S34), KCTD (Figure S35), SOX (Figure S44) and TNRC (Figure S46) families (see Additional file 6): In all the analyzed genomes these families have two or three 2R-generated subtype genes located on the same chromosome regions with 3R-generated duplicates on other chromosomes (see Additional file 4). The GRIN2 PhyML tree is shown as an example in Figure 3 and the FSCN PhyML tree can be seen in Figure 4.

**Figure 7:**
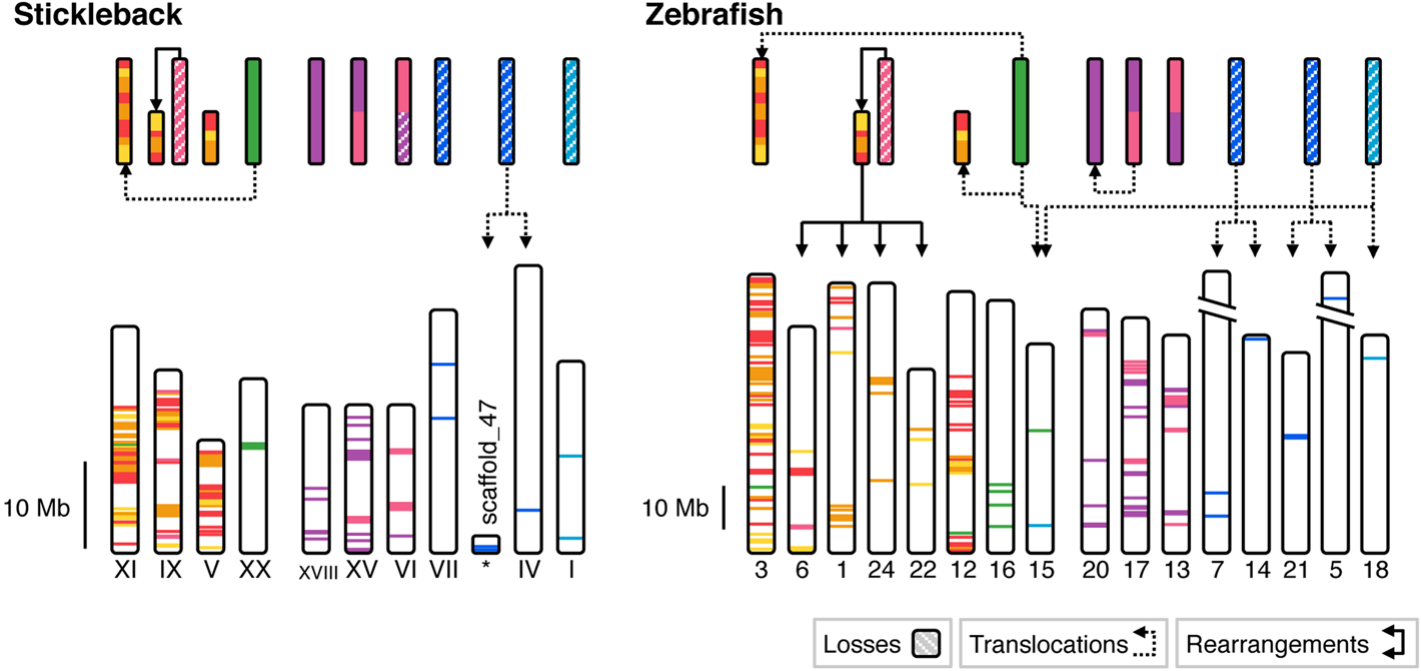
**Continued from Figure**6**.** Paralogous chromosome regions in the stickleback and zebrafish genomes. More rearrangements could be identified in the zebrafish genome than in the stickleback or medaka genomes.

This situation allows us to infer the scenario presented in Figure 6: Three of the four 2R-generated paralogous chromosome blocks were fused into the same chromosome in the ray-finned fish lineage sometime after the spotted gar had branched off approximately 350 MYA and before 3R in the teleost lineage (for time point estimates see *Amores et al. (2012)*[45]). After the 3R event and before the last common ancestor of the studied species, the now duplicated fused chromosome blocks exchanged paralogs and subsequently one of them was split by fission events. In all the analyzed teleost genomes we observe these fused and rearranged regions on at least three chromosomes (Figures 6 and 7). There seem to have been more fissions and rearrangements in the zebrafish lineage (Figure 7). It is likely that many of the rearrangements occurred as part of larger blocks and subsequently local rearrangements have jumbled the ancestral order. This scenario is corroborated by the orthology relationships inferred from the phylogenetic analyses of the neighboring families (see Additional file 6, Figures S21-S50). The fact that it is 2R-generated duplicates that have been co-located by the chromosome fusions, and not primarily 3R-generated duplicates, shows that the fusions occurred before 3R.

We could see similar chromosomal rearrangements in the paralogous regions bearing *SSTR1, -4* and -*6* genes, although not to the same extent. Due to the lower degree of SSTR gene retention after 2R and 3R in this paralogon, fewer neighboring families could be identified as belonging to the paralogy block. Nonetheless some gene families seem to have translocated duplicates between homologous chromosomes after 3R (Figure 6). The highest degree of such translocations can be seen in the zebrafish where the ABDH (Figure S4), FOXA (Figure S7), JAG (Figure S9), NIN (Figure S10), NKX2 (Figure S11), PAX (Figure S12), PYG (Figure S13), RALGAPA (Figure S14) and VSX (Figure S20) families have duplicates of 2R-generated subtypes located on the same chromosome (see Additional file 3).

There have been some indications of these translocations in previously published large-scale genomic analyses. For instance, in the analysis of the published medaka genome [5] the rearrangements after 3R between the *SSTR2, -3* and -*5*-paralogous regions on chromosomes 1, 8 and 19 are apparent. Our analyses allow us to resolve the events in greater detail: We conclude that these rearrangements in the teleost lineage were preceded by fusions of 2R-generated chromosome blocks before 3R, with subsequent paralog translocations and chromosome fissions after 3R (Figure 6). This fusion scenario is supported by a large-scale reconstruction of the ancestral vertebrate genome [4], where these reorganizations were concluded from comparative genomic analyses including the medaka. However, these analyses suggested that the fusions occurred before the divergence of lobe-finned and ray-finned fishes. Our conserved synteny analysis on the other hand shows that the tetrapod genomes have no signs of ancestral fusions in this paralogon (Figure 6, see Additional file 4). Together with the locations of the *SSTR2, -3* and -*5* genes on different linkage groups in the spotted gar genome (Table 1), our data instead point towards a time frame for the chromosomal fusions after the divergence of the gar lineage and before 3R in the teleost lineage. Both these large-scale analyses also support our scenario for the rearrangements between *SSTR1, -4* and -*6*-paralogous regions in the teleost lineage after 3R.

The recent mapping of the spotted gar genome [45] concluded that its genome organization is more similar to that of the human genome than to teleost genomes. We were able to predict sequences for all SSTR genes except *SSTR4* in the genome of the spotted gar, and located them to five different genomic linkage groups (Table 1). The cited analyses of conserved synteny between the spotted gar genome and the human, zebrafish and stickleback genomes are concurrent with our own, and demonstrate that the linkage groups we have identified as SSTR-bearing in the spotted gar share conserved synteny with SSTR-bearing chromosome regions in the other genomes (see supporting information in *Amores et al. (2011)*[45]).

It is to be expected that duplicated chromosomes, as well as duplicated chromosomal regions that display similarity, can undergo rearrangements such as translocation to the same chromosome. We were surprised to find that regions that arose as separate chromosomes in 2R, perhaps 500 MYA, have been fused in the ray-finned fish lineage and subsequently exchanged 2R-generated paralogs after the 3R event approximately 300 MYA. Any such rearrangements require extensive analyses in order to be disentangled. We had completed our comprehensive analyses arriving at the scenario shown in Figure 6 when the spotted gar genome became available and confirmed our suggested scenario. The teleost rearrangements described here may severely hamper efforts to use conservation of synteny for identification of orthologs between teleosts and other vertebrates. Fortunately, the spotted gar constitutes a very important out-group for comparison with chromosomal events involving or surrounding 3R and it will greatly facilitate such analyses [45].

### Implications for synteny analyses and orthology assignment

Our studies of the two vertebrate paralogons bearing SSTR genes have potentially far-reaching implications for comparative genomic studies such as analyses of conserved synteny. The identification of conserved synteny is essential for the correct assignment of orthology and paralogy relationships between genes, and therefore for the evolutionary studies of gene families [46].

We describe here how 2R-generated duplicated chromosome blocks fused in the ray-finned fish lineage, and how subsequently these fused blocks duplicated in 3R and exchanged paralogs between each other, likely in blocks, blurring much of the conserved synteny patterns generated by the whole genome duplications. There have also been intra-chromosomal rearrangements within these chromosomal blocks, as well as a fission event splitting one of the 3R-duplicated blocks. At this point it is worth noting that the scenarios describing the evolution of the *SSTR2, -3* and -*5*-bearing chromosome regions and the *SSTR1, -4* and -*6*-bearing regions differ, with the latter showing no sign of fusions after 2R and inter-chromosomal exchange of paralogs only after 3R (Figures 6 and 7).

These types of rearrangements of the genomic structure make it exceedingly complicated to sort out the evolution of genomic regions and to infer orthology and paralogy relationships within gene families. We show that it is possible to resolve these events if one considers both the positional data between several different genomes as well as the phylogenies of the gene families shared between the chromosome regions. These analyses also demonstrate the importance of having the appropriate out-groups to determine the (relative) time points of the events. We could confirm the likely ancestral paralogy relationships between the chromosome regions by comparing our findings against the genomes of the spotted gar and the coelacanth, which were released during the final stages of our analyses. The spotted gar genome, which has been assembled to linkage groups, proved essential to confirm the ancestral location of the SSTR genes on different chromosomes, and therefore to support both the duplication of the chromosome regions in 2R and the time window of the chromosome block fusions.

The chromosomal rearrangements that we have described here undoubtedly complicate the assignment of orthology based on synteny analyses. For the SSTR family we found that the assignment of SSTR genes to specific subtypes needs to take into consideration firstly that there is a sixth previously undescribed ancestral vertebrate subtype, *SSTR6*, which is more closely related to *SSTR1* and *SSTR*4; secondly, that teleost fishes may have additional paralogs resulting from the shared third whole genome duplication (3R); and thirdly that additional duplicates have been generated by independent fourth genome duplications in some lineages. The teleost *SSTR6* sequences that we have identified in this study were annotated as *SSTR1a* in the zebrafish genome database and *SSTR4* in the stickleback and fugu genome databases.

## Conclusions

By combining analyses of conserved synteny with phylogenetic data we can conclude that two vertebrate ancestral SSTR genes on different chromosomes diversified in the basal vertebrate whole genome duplications, 2R, one giving rise to *SSTR1, -4* and -*6* subtype genes, and one giving rise to *SSTR2, -3* and -*5* subtype genes. The *SSTR6* subtype was previously unrecognized, and could be identified in all teleost fish genomes, the spotted gar genome as well as the genome of the Comoran coelacanth. Conversely, *SSTR4* subtype genes could only be identified in the analyzed tetrapod genes as well as the coelacanth. Taken together these results indicate that six SSTR subtype genes were ancestral to both lobe-finned and ray-finned fishes, but that reciprocal losses have occurred. Subsequently *SSTR2, -3* and -*5* conserved duplicates from the teleost-specific whole genome duplication, 3R. Although there have been losses of SSTR subtype genes, the paralogous genome regions could be identified in both tetrapod and teleost genomes. The positional and phylogenetic data from the analysis of conserved synteny indicate that there have been significant rearrangements between paralogous chromosome regions in the teleost genomes, especially between *SSTR2, -3* and -*5*-bearing chromosome regions. These rearrangements would explain the co-localization of *SSTR2, -3* and -*5* genes in several teleost genomes. That these rearrangements occurred in the teleost lineage is corroborated by comparison with the spotted gar genome, representing a lineage that diverged before teleost evolution.

## Methods

### Identification of SSTR sequences in Ensembl genome databases

Amino acid sequences of SSTR family members were identified in the Ensembl genome browser (http://www.ensembl.org) using the automatic protein family prediction feature. All SSTR sequences and their locations have been verified against Ensembl release 67 (May 2012). In most analyzed genomes the identified “somatostatin receptor type” protein family included somatostatin receptors of *SSTR1*, *-2*, *-3*, *-4* and -*5*-type as well as neuropeptide B/W receptors of *NPBWR1* and -*2*-type. The NPBWRs share sequence similarity to both SSTRs and opioid receptors, however phylogenetic analysis as well as their chromosomal locations indicate that they constitute a separate family of GPCRs [9]. Hence only the SSTR sequences were considered in our analyses.

The SSTR sequences from the following Ensembl genome databases were collected and their database identifiers and locations noted: *Homo sapiens* (human), *Mus musculus* (mouse), *Canis familiaris* (dog), *Monodelphis domestica* (grey short-tailed opossum), *Gallus gallus* (chicken), *Anolis carolinensis* (Carolina anole lizard), *Silurana (Xenopus) tropicalis* (Western clawed frog), *Latimeria chalumnae* (Comoran coelacanth), *Danio rerio* (zebrafish), *Oryzias latipes* (medaka), *Gasterosteus aculeatus* (three-spined stickleback), *Tetraodon nigroviridis* (green spotted pufferfish), *Takifugu rubripes* (fugu), *Ciona intestinalis* (vase tunicate) and *Drosophila melanogaster* (fruit fly). Database identifiers, location data and annotation notes of all SSTR sequences, as well as genome assembly versions for each species, are listed in Additional file 7.

To account for possible failures in the automatic identification of SSTR protein family members TBLASTN searches were also carried out in the Ensembl databases as well as in the National Center for Biotechnology Information (NCBI) Reference Sequence and trace archive databases using the known human SSTR sequences as queries. *Branchiostoma floridae* (Florida lancelet, amphioxus) genomic scaffolds were sought by TBLASTN searches in the NCBI Reference Sequence database using the known human family member sequences as queries. Additionally, complementary searches for teleost fish sequences were performed in the NCBI reference sequence database using the identified zebrafish *SSTR1* and *SSTR6* sequences.

### Identification of SSTR sequences in the Lepisosteus oculatus genome

SSTR sequences were sought in the *Lepisosteus oculatus* (spotted gar) genome assembly *LepOcu1* (GenBank ID: GCA_000242695.1) available from NCBI (http://www.ncbi.nlm.nih.gov/genome/assembly/327908/). The sequences of the assembled linkage groups as well as unplaced scaffolds were downloaded and a local search database was set up. TBLASTN searches were carried out in this local database applying the BLAST+ 2.2.26 executable application available from ftp://ftp.ncbi.nlm.nih.gov/blast/executables/blast+/LATEST/ with the known human SSTR sequences as well as the identified coelacanth sequences as search queries. The near full-length BLAST hits were evaluated by reciprocal tblastn searches in the NCBI reference sequence database and those that matched identified SSTR sequences were included in preliminary neighbor joining (NJ) trees (see “*Phylogenetic analyses*” below) to assert their identities. The positions within the linkage groups of those BLAST hits that clustered confidently within the SSTR NJ tree were noted and the corresponding genomic sequences were inspected in order to predict the full length of the SSTR genes in the spotted gar (see “*Sequence alignments and editing of gene and protein sequences*” below).

### Identification and analysis of neighboring gene families/Conserved synteny analysis

Lists of gene predictions corresponding to the different SSTR-bearing chromosome blocks were downloaded using the BioMart function in the Ensembl database version 56. The chromosome blocks were defined as 15 Mb in each direction of the SSTR gene in question, although in many cases this definition encompassed the entire chromosome. These blocks were compared with each other in order to identify those gene families, as defined by Ensembl’s automatic protein family prediction, that are represented on several of the blocks across different species.

For the analysis of the *SSTR1*, *-4* and -*6*-bearing regions, the human and chicken chromosome blocks carrying the *SSTR1* and *SSTR4* genes were compared with each other. The gene families that were represented on both chromosomes in the human genome were selected for the analysis of conserved synteny and this list was complemented with those gene families that were represented on both chicken chromosomes as well as at least one of the human chromosomes. In this way we could account for any possible lineage-specific rearrangements in any of these genomes. The chromosome blocks in the human genome (assembly GRCh36) were between map positions 23 Mb and 53 Mb on chromosome 14 and between 8 Mb and 38 Mb on chromosome 20. The chromosome blocks in the chicken genome (assembly WASHUC2) were between map positions 24 Mb and 54 Mb on chromosome 5 and between 1 bp and 18 Mb on chromosome 3. These blocks represent the chromosome regions bearing *SSTR1* and *SSTR4* genes respectively in each species. Teleost genomes were not considered in this selection of neighboring gene families since there seems to have been a lineage-specific loss of *SSTR4* early in ray-finned fish evolution. Our preliminary phylogenetic analysis indicated that teleosts, spotted gar and coelacanth had another distinct SSTR gene instead, *SSTR6*, which we could take advantage of in the analysis of conserved synteny. This gene has not been assigned to a chromosome location except for in the zebrafish genome. We attempted including the chromosome regions of zebrafish *SSTR1* and -*6* in the selection of neighboring gene families, but this provided no additional ones.

For the analysis of the *SSTR2*, *-3* and -*5*-bearing regions, the chicken and stickleback chromosome blocks were both used. The gene families that were represented on all three chromosomes in each of these genomes were chosen for the analysis of conserved synteny. The chromosome blocks in the chicken genome (assembly WASHUC2) were between map positions 38 Mb and 69 Mb on chromosome 1, as well as the whole of chromosomes 14 (approximately 15.8 Mb) and 18 (approximately 10.9 Mb). The blocks in the stickleback genome (assembly BROADS1) correspond to the full linkage groups V (approximately 12.25 Mb), IX (approximately 20.24 Mb) and XI (approximately 16.20 Mb). Linkage groups V and IX carry the *SSTR2b* and *SSTR5b* genes respectively, and linkage group XI carries three SSTR genes: *SSTR2a*, *SSTR5a* and *SSTR3*. The stickleback genome was favored over other teleost genomes as all the SSTR genes predicted in this genome assembly have been mapped.

The predicted amino acid sequences of all the identified protein family members were downloaded for subsequent alignment and phylogenetic analysis, and the locations of the corresponding predicted genes were noted (see Additional files 8 and 9). Locations have been verified against Ensembl version 67 (May 2012) to ensure the information is up to date. To a large extent the same species were included in the phylogenetic analyses of the neighboring gene families as in the SSTR tree, with the following exceptions: coelacanth and spotted gar sequences were not considered and green spotted pufferfish and/or fugu sequences were only included when the preliminary phylogenetic analyses showed inconclusive teleost fish topologies. Additionally, sequences from the *Macropus eugenii* (tammar wallaby, assembly 1.0), *Taeniopygia guttata* (zebra finch, assembly 3.2.4), *Meleagris gallopavo* (turkey, assembly 2.01), *Ciona savignyii* (transparent tunicate, assembly 2.0) and *Branchiostoma floridae* (Florida lancelet, amphioxus, assembly 2.0) genome databases were used to complement missing gene predictions in the genome databases for grey short-tailed opossum, chicken and vase tunicate for some gene families. For those few gene families where no fruit fly, amphioxus or tunicate sequences could be identified, the *Caenorhabditis elegans* predicted protein family members were collected from the Ensembl database (assembly WBcel215). Database identifiers, location data and annotation notes of all neighboring family sequences are included in supplemental tables (see Additional files 8 and 9).

### Sequence alignments and editing of gene and protein sequences

The identified amino acid sequences were aligned using the ClustalWS sequence alignment program with standard settings (Gonnet weight matrix, gap opening penalty 10.0 and gap extension penalty 0.20) through the JABAWS 2 tool in Jalview 2.7 [47]. The alignments were manually inspected and edited in Jalview 2.7 in order to curate wrongly predicted sequences and adjust poorly aligned sequence stretches. Short, incomplete or highly diverging amino acid sequence predictions were curated manually by analyzing the corresponding genomic sequence (including full intron sequences and flanking regions) with respect to consensus for splice donor and acceptor sites and sequence homology to other family members. In this way erroneous automatic exon predictions and exons that had not been predicted could be ratified.

### Phylogenetic analyses

Phylogenetic trees were made using the Phylogenetic Maximum Likelihood (PhyML) method [48] supported by a non-parametric bootstrap analysis of 100 replicates and assuming the LG matrix of amino acid substitution by Le and Gascuel [49]. This method was applied using the web-application of the PhyML 3.0 algorithm available at http://www.atgc-montpellier.fr/phyml/ or the executable PhyML-aBayes (3.0.1 beta) program with the following settings: amino acid frequencies (equilibrium frequencies), proportion of invariable sites (with optimised *p-invar*) and gamma-shape parameters were estimated from the datasets; the number of substitution rate categories was set to 8; BIONJ was chosen to create the starting tree and the nearest neighbor interchange (NNI) tree improvement method was used to estimate the best topology; both tree topology and branch length optimization were chosen.

Initially, phylogenetic trees were made using the neighbor joining (NJ) method applied through ClustalX 2.0 [50] with standard settings and a non-parametric bootstrap analysis with 1000 replicates. These trees have been included for the neighboring gene families in Additional files 5 and 6 in order to complement the PhyML tree topologies and provide a reference for discussion in the cases where tree topologies were inconclusive (see Results). For both NJ and PhyML tree topologies, bootstrap values higher than 50% were considered supportive.

For the SSTR-family tree (Figure 1) more careful measures were taken in order to account for the larger amount of protein subtypes and animal taxa in this tree compared to the neighboring gene families. The Phylogenetic Maximum Likelihood analysis was repeated using both a non-parametric bootstrap analysis of 100 replicates, and an SH-like approximate likelihood ratio test (aLRT) [51], in both cases selecting both NNI and subtree pruning and regrafting (SPR) tree improvement methods rather than only NNI. Additionally, the amino acid substitution model for the phylogenetic analysis was chosen using ProtTest 3.0 [52] with the following settings: Likelihood scores were computed selecting the JTT, LG, DCMut, Dayhoff, WAG, Blosum62 and VT substitution model matrices, with no add-ons and a Fixed BioNJ JTT base tree. Based on this analysis the JTT model of amino acid substitution was chosen.

In most cases the identified fruit fly sequences were used as out-groups to root the trees, and where such a sequence could not be found the identified amphioxus or tunicate sequences were used as the out-group instead. The inclusion of amphioxus and/or tunicate in the phylogenetic analyses provides the relative dating for the time window of the 2R events. For two gene families *C. elegans* sequences had to be identified due to the lack of fruit fly sequences. For the SSTR-family tree (Figure 1) the human kisspeptin receptor (*KISS1R or GPR54*) sequence was chosen as an out-group in order to accurately show the branching point of the identified fruit fly SSTR-family genes. Kisspeptin receptors are GPCRs closely related to the somatostatin receptors [53] (see also Additional file 5; Figure S15 in *Nordström et al. (2008)*[33]), diverging before the protostome-deuterostome split, therefore providing a reasonable out-group for our phylogenetic analysis of the SSTR family.

### Description of additional files

The following additional data files are available with the online version of this paper. The spreadsheets in Additional files 7, 8 and 9 include comprehensive information about all sequences analyzed in this study, such as database identifiers, location data and annotation notes. Figures of all phylogenetic analyses used in the study are included in Additional files 2,5,6. The positional data underlying our evolutionary scenario is presented in Additional files 3 and 4. All final curated sequence alignments made for the phylogenetic analyses, as well as the original rooted phylogenetic tree files, have been provided as citable file sets with persistent identifiers - see references [54,55]. Detailed notes on the identification of SSTR sequences in the genome databases, as well as detailed descriptions of the neighboring family tree topologies, are included in Additional file 1.

## Competing interests

The authors declare that they have no competing interests.

## Authors’ contributions

DOD and GS participated in the study design, performed phylogenetic and chromosome analyses and co-wrote the article. CAB performed genome database searches, sequence analyses and alignments as well as phylogenetic and chromosome analyses. DL conceived and co-designed the study, participated in all analyses and co-wrote the article. All authors participated in the draft of the manuscript and have read and approved the final version.

## Supplementary Material

Additional file 1 **Supplemental notes 1–3.** Detailed descriptions of the results, including the identification of SSTR sequences in genome databases as well as the phylogenetic analyses of neighboring gene families. Click here for file

Additional file 2 **Figures S1–S3.** All phylogenetic analyses of the SSTR gene family. Click here for file

Additional file 3 **Table S4.** Positional data for the *SSTR1, -4* and -*6*-bearing chromosome regions. The members of the identified neighboring gene families in these chromosome regions are charted by species and chromosome/genomic scaffold. These charts show the identified paralogous chromosome regions in the human, chicken, medaka, stickleback and zebrafish genomes. Each species is included in a separate tab in the spreadsheet. Click here for file

Additional file 4 **Table S5.** Positional data for the *SSTR2, -3* and -*5*-bearing chromosome regions. The members of the identified neighboring gene families in these chromosome regions are charted by species and chromosome/genomic scaffold. These charts show the identified paralogous chromosome regions in the human, chicken, medaka, stickleback and zebrafish genomes. Each species is included in a separate tab in the spreadsheet. Click here for file

Additional file 5 **Figures S4–S20.** Phylogenetic trees of the *SSTR1, -4* and -*6*-neighboring gene families. Figures are numbered S4-S20 and include both neighbor joining and phylogenetic maximum likelihood trees of the gene families described in Table 2. Click here for file

Additional file 6 **Figures S21–S50.** Phylogenetic trees of the *SSTR2, -3* and -*5*-neighboring gene families. Figures are numbered S21-S50 and include both neighbor joining and phylogenetic maximum likelihood trees of the gene families described in Table 3. Click here for file

Additional file 7 **Table S1.** Database identifiers, location data and annotation notes of all SSTR sequences identified and included in this study. Click here for file

Additional file 8 **Table S2.** Database identifiers, location data and annotation notes of *SSTR1,-4* and -*6*-neighboring gene family sequences, including information for those gene families that were discarded from the analysis. Each gene family is included in a separate tab in the spreadsheet. Click here for file

Additional file 9 **Table S3.** Database identifiers, location data and annotation notes of *SSTR2,-3* and -*5*-neighboring gene family sequences, including information for those gene families that were discarded from the analysis. Each gene family is included in a separate tab in the spreadsheet. Click here for file

## References

[B1] Dehal P Boore JL Two rounds of whole genome duplication in the ancestral vertebrate PLoS Biol 2005 3 e31410.1371/journal.pbio.0030314 10.1371/journal.pbio.0030314 PMC1197285 16128622

[B2] Jaillon O Aury J-M Brunet F Petit J-L Stange-Thomann N Mauceli E Bouneau L Fischer C Ozouf-Costaz C Bernot A Nicaud S Jaffe D Fisher S Lutfalla G Dossat C Segurens B Dasilva C Salanoubat M Levy M Boudet N Castellano S Anthouard V Jubin C Castelli V Katinka M Vacherie B Biémont C Skalli Z Cattolico L Poulain J Genome duplication in the teleost fish Tetraodon nigroviridis reveals the early vertebrate proto-karyotype Nature 2004 431 946 57 10.1038/nature03025 15496914

[B3] Putnam NH Butts T Ferrier DEK Furlong RF Hellsten U Kawashima T Robinson-Rechavi M Shoguchi E Terry A Yu J-K Benito-Gutiérrez E Dubchak I Garcia-Fernàndez J Gibson-Brown JJ Grigoriev IV Horton AC de Jong PJ Jurka J Kapitonov VV Kohara Y Kuroki Y Lindquist E Lucas S Osoegawa K Pennacchio LA Salamov AA Satou Y Sauka-Spengler T Schmutz J Shin-I T The amphioxus genome and the evolution of the chordate karyotype Nature 2008 453 1064 71 10.1038/nature06967 18563158

[B4] Nakatani Y Takeda H Kohara Y Morishita S Reconstruction of the vertebrate ancestral genome reveals dynamic genome reorganization in early vertebrates Genome Res 2007 17 1254 65 10.1101/gr.6316407 17652425 PMC1950894

[B5] Kasahara M Naruse K Sasaki S Nakatani Y Qu W Ahsan B Yamada T Nagayasu Y Doi K Kasai Y Jindo T Kobayashi D Shimada A Toyoda A Kuroki Y Fujiyama A Sasaki T Shimizu A Asakawa S Shimizu N Hashimoto S-I Yang J Lee Y Matsushima K Sugano S Sakaizumi M Narita T Ohishi K Haga S Ohta F The medaka draft genome and insights into vertebrate genome evolution Nature 2007 447 714 9 10.1038/nature05846 17554307

[B6] Flicek P Amode MR Barrell D Beal K Brent S Carvalho-Silva D Clapham P Coates G Fairley S Fitzgerald S Gil L Gordon L Hendrix M Hourlier T Johnson N Kähäri AK Keefe D Keenan S Kinsella R Komorowska M Koscielny G Kulesha E Larsson P Longden I McLaren W Muffato M Overduin B Pignatelli M Pritchard B Riat HS Ensembl 2012 Nucleic Acids Res 2012 40 D84 90 10.1093/nar/gkr991 22086963 PMC3245178

[B7] Meyer A Van de Peer Y From 2R to 3R: evidence for a fish-specific genome duplication (FSGD) Bioessays 2005 27 937 45 10.1002/bies.20293 16108068

[B8] Sundström G Dreborg S Larhammar D Concomitant duplications of opioid peptide and receptor genes before the origin of jawed vertebrates PLoS One 2010 5 e1051210.1371/journal.pone.0010512 10.1371/journal.pone.0010512 PMC2865548 20463905

[B9] Dreborg S Sundström G Larsson TA Larhammar D Evolution of vertebrate opioid receptors Proc Natl Acad Sci U S A 2008 105 15487 9210 1073/pnas.0805590105 18832151 10.1073/pnas.0805590105 PMC2563095

[B10] Sundström G Larsson TA Brenner S Venkatesh B Larhammar D Evolution of the neuropeptide Y family: new genes by chromosome duplications in early vertebrates and in teleost fishes Gen Comp Endocrinol 2008 155 705 16 10.1016/j.ygcen.2007.08.016 17950734

[B11] Widmark J Sundström G Ocampo Daza D Larhammar D Differential evolution of voltage-gated sodium channels in tetrapods and teleost fishes Mol Biol Evol 2011 28 859 71 10.1093/molbev/msq257 20924084

[B12] Larsson TA Olsson F Sundström G Lundin L-G Brenner S Venkatesh B Larhammar D Early vertebrate chromosome duplications and the evolution of the neuropeptide Y receptor gene regions BMC Evol Biol 2008 8 184 10.1186/1471-2148-8-184 18578868 PMC2453138

[B13] Ocampo Daza D Sundström G Bergqvist CA Duan C Larhammar D Evolution of the insulin-like growth factor binding protein (IGFBP) family Endocrinology 2011 152 2278 89 10.1210/en.2011-0047 21505050

[B14] Dos Santos S Mazan S Venkatesh B Cohen-Tannoudji J Quérat B Emergence and evolution of the glycoprotein hormone and neurotrophin gene families in vertebrates BMC Evol Biol 2011 11 332 10.1186/1471-2148-11-332 22085792 PMC3280201

[B15] Braasch I Volff J-N Schartl M The endothelin system: evolution of vertebrate-specific ligand-receptor interactions by three rounds of genome duplication Mol Biol Evol 2009 26 783 99 10.1093/molbev/msp015 19174480

[B16] Brazeau P Vale W Burgus R Ling N Butcher M Rivier J Guillemin R Hypothalamic polypeptide that inhibits the secretion of immunoreactive pituitary growth hormone Science (New York, N.Y.) 1973 179 77 9 10.1126/science.179.4068.77 4682131

[B17] Viollet C Lepousez G Loudes C Videau C Simon A Epelbaum J Somatostatinergic systems in brain: networks and functions Mol Cell Endocrinol 2008 286 75 87 10.1016/j.mce.2007.09.007 17997029

[B18] de Lecea L Ruiz-Lozano P Danielson PE Peelle-Kirley J Foye PE Frankel WN Sutcliffe JG Cloning, mRNA expression, and chromosomal mapping of mouse and human preprocortistatin Genomics 1997 42 499 506 10.1006/geno.1997.4763 9205124

[B19] Liu Y Lu D Zhang Y Li S Liu X Lin H The evolution of somatostatin in vertebrates Gene 2010 463 21 8 10.1016/j.gene.2010.04.016 20472043

[B20] Tostivint H Lihrmann I Vaudry H New insight into the molecular evolution of the somatostatin family Mol Cell Endocrinol 2008 286 5 17 10.1016/j.mce.2008.02.029 18406049

[B21] Olias G Viollet C Kusserow H Epelbaum J Meyerhof W Regulation and function of somatostatin receptors J Neurochem 2004 89 1057 91 10.1111/j.1471-4159.2004.02402.x 15147500

[B22] Meyerhof W The elucidation of somatostatin receptor functions: a current view Rev Physiol Biochem Pharmacol 1998 133 55 108 9600011 10.1007/BFb0000613

[B23] Nelson LE Sheridan MA a: Regulation of somatostatins and their receptors in fish Gen Comp Endocrinol 2005 142 117 33 10.1016/j.ygcen.2004.12.002 15862556

[B24] Bossis I Porter TE Identification of the somatostatin receptor subtypes involved in regulation of growth hormone secretion in chickens Mol Cell Endocrinol 2001 182 203 13 10.1016/S0303-7207(01)00561-5 11514055

[B25] Geris KL de Groef B Rohrer SP Geelissen S Kühn ER Darras VM Identification of somatostatin receptors controlling growth hormone and thyrotropin secretion in the chicken using receptor subtype-specific agonists J Endocrinol 2003 177 279 86 10.1677/joe.0.1770279 12740016

[B26] Moaeen-ud-Din M Yang LG Evolutionary history of the somatostatin and somatostatin receptors J Genet 2009 88 41 53 10.1007/s12041-009-0006-1 19417543

[B27] Haiyan D Wensheng L Haoran L Comparative analyses of sequence structure, evolution, and expression of four somatostatin receptors in orange-spotted grouper (Epinephelus coioides) Mol Cell Endocrinol 2010 323 125 36 10.1016/j.mce.2010.03.016 20347929

[B28] Kittilson JD Slagter BJ Martin LE Sheridan MA a: Isolation, characterization, and distribution of somatostatin receptor subtype 2 (SSTR 2) mRNA in rainbow trout (Oncorhynchus mykiss), and regulation of its expression by glucose Comp Biochem Physiol A Mol Integr Physiol 2011 160 237 44 10.1016/j.cbpa.2011.06.009 21693197

[B29] Kreienkamp H-J Larusson HJ Witte I Roeder T Birgul N Honck H-H Harder S Ellinghausen G Buck F Richter D Functional annotation of two orphan G-protein-coupled receptors, Drostar1 and -2, from Drosophila melanogaster and their ligands by reverse pharmacology J Biol Chem 2002 277 39937 43 10.1074/jbc.M206931200 12167655

[B30] Veenstra JA Allatostatin C and its paralog allatostatin double C: the arthropod somatostatins Insect Biochem Mol Biol 2009 39 161 70 10.1016/j.ibmb.2008.10.014 19063967

[B31] Holland LZ Albalat R Azumi K Benito-Gutiérrez E Blow MJ Bronner-Fraser M Brunet F Butts T Candiani S Dishaw LJ Ferrier DEK Garcia-Fernàndez J Gibson-Brown JJ Gissi C Godzik A Hallböök F Hirose D Hosomichi K Ikuta T Inoko H Kasahara M Kasamatsu J Kawashima T Kimura A Kobayashi M Kozmik Z Kubokawa K Laudet V Litman GW McHardy AC The amphioxus genome illuminates vertebrate origins and cephalochordate biology Genome Res 2008 18 1100 11 10.1101/gr.073676.107 18562680 PMC2493399

[B32] Delsuc F Tsagkogeorga G Lartillot N Philippe H Additional molecular support for the new chordate phylogeny Genesis 2008 46 592 604 10.1002/dvg.20450 19003928

[B33] Nordström KJV Fredriksson R Schiöth HB The amphioxus (Branchiostoma floridae) genome contains a highly diversified set of G protein-coupled receptors BMC Evol Biol 2008 8 9 10.1186/1471-2148-8-9 18199322 PMC2246102

[B34] Lin X Janovick JA Brothers S Conn PM Peter RE Molecular cloning and expression of two type one somatostatin receptors in goldfish brain Endocrinology 1999 140 5211 9 10.1210/en.140.11.5211 10537151

[B35] Lin X Janovick JA Cardenas R Conn PM Peter RE Molecular cloning and expression of a type-two somatostatin receptor in goldfish brain and pituitary Mol Cell Endocrinol 2000 166 75 87 10.1016/S0303-7207(00)00278-1 10996426

[B36] Lin X Nunn C Hoyer D Rivier J Peter RE Identification and characterization of a type five-like somatostatin receptor in goldfish pituitary Mol Cell Endocrinol 2002 189 105 16 10.1016/S0303-7207(01)00745-6 12039069

[B37] Lin X Peter RE Somatostatin-like receptors in goldfish: cloning of four new receptors Peptides 2003 24 53 63 10.1016/S0196-9781(02)00276-0 12576085

[B38] Larhammar D Risinger C Molecular genetic aspects of tetraploidy in the common carp Cyprinus carpio Mol Phylogenet Evol 1994 3 59 68 10.1006/mpev.1994.1007 8025730

[B39] David L Blum S Feldman MW Lavi U Hillel J Recent duplication of the common carp (Cyprinus carpio L.) genome as revealed by analyses of microsatellite loci Mol Biol Evol 2003 20 1425 34 10.1093/molbev/msg173 12832638

[B40] Zupanc GKH Siehler S Jones EMC Seuwen K Furuta H Hoyer D Yano H Molecular cloning and pharmacological characterization of a somatostatin receptor subtype in the gymnotiform fish Apteronotus albifrons Gen Comp Endocrinol 1999 115 333 45 10.1006/gcen.1999.7316 10480984

[B41] Trainor BC Hofmann HA Somatostatin regulates aggressive behavior in an African cichlid fish Endocrinology 2006 147 5119 25 10.1210/en.2006-0511 16887916

[B42] Slagter BJ Sheridan MA Differential expression of two somatostatin receptor subtype 1 mRNAs in rainbow trout (Oncorhynchus mykiss) J Mol Endocrinol 2004 32 165 77 10.1677/jme.0.0320165 14766000

[B43] Slagter BJ Kittilson JD Sheridan MA Somatostatin receptor subtype 1 and subtype 2 mRNA expression is regulated by nutritional state in rainbow trout (Oncorhynchus mykiss) Gen Comp Endocrinol 2004 139 236 44 10.1016/j.ygcen.2004.09.008 15560870

[B44] Hagemeister AL Kittilson JD Bergan HE Sheridan MA a: Rainbow trout somatostatin receptor subtypes SSTR1A, SSTR1B, and SSTR2 differentially activate the extracellular signal-regulated kinase and phosphatidylinositol 3-kinase signaling pathways in transfected cells J Mol Endocrinol 2010 45 317 27 10.1677/JME-10-0046 20732992

[B45] Amores A Catchen J Ferrara A Fontenot Q Postlethwait JH Genome evolution and meiotic maps by massively parallel DNA sequencing: spotted gar, an outgroup for the teleost genome duplication Genetics 2011 188 799 808 10.1534/genetics.111.127324 21828280 PMC3176089

[B46] Catchen JM Conery JS Postlethwait JH Automated identification of conserved synteny after whole-genome duplication Genome Res 2009 19 1497 505 10.1101/gr.090480.108 19465509 PMC2720179

[B47] Waterhouse AM Procter JB Martin DMA Clamp M Barton GJ Jalview Version 2 - a multiple sequence alignment editor and analysis workbench Bioinformatics 2009 25 1189 91 10.1093/bioinformatics/btp033 19151095 PMC2672624

[B48] Guindon S Dufayard J-F Lefort V Anisimova M Hordijk W Gascuel O New algorithms and methods to estimate maximum-likelihood phylogenies: assessing the performance of PhyML 3.0 Syst Biol 2010 59 307 21 10.1093/sysbio/syq010 20525638

[B49] Le SQ Gascuel O An improved general amino acid replacement matrix Mol Biol Evol 2008 25 1307 20 10.1093/molbev/msn067 18367465

[B50] Larkin MA Blackshields G Brown NP Chenna R McGettigan PA McWilliam H Valentin F Wallace IM Wilm A Lopez R Thompson JD Gibson TJ Higgins DG Clustal W and Clustal X version 2.0 Bioinformatics 2007 23 2947 8 10.1093/bioinformatics/btm404 17846036

[B51] Anisimova M Gascuel O Approximate likelihood-ratio test for branches: A fast, accurate, and powerful alternative Syst Biol 2006 55 539 52 10.1080/10635150600755453 16785212

[B52] Abascal F Zardoya R Posada D ProtTest: selection of best-fit models of protein evolution Bioinformatics 2005 21 2104 5 10.1093/bioinformatics/bti263 15647292

[B53] Fredriksson R Lagerström MC Lundin L-G Schiöth HB The G-protein-coupled receptors in the human genome form five main families. Phylogenetic analysis, paralogon groups, and fingerprints Mol Pharmacol 2003 63 1256 72 10.1124/mol.63.6.1256 12761335

[B54] Ocampo Daza D Sundström G Bergqvist CA Larhammar D Phylogenetic analyses of the somatostatin receptor gene family Figshare 2012 http://dx.doi.org/10.6084/m9.figshare.94213

[B55] Ocampo Daza D Sundström G Bergqvist CA Larhammar D Phylogenetic analyses of syntenic gene families in SSTR gene-bearing chromosome regions Figshare 2012 http://dx.doi.org/10.6084/m9.figshare.94266

