# Supplementary material for: The evolution of vertebrate somatostatin receptors and their gene regions involves extensive chromosomal rearrangements
Source: BMC Evol Biol. 2012 Nov 29;12:231. doi: 10.1186/1471-2148-12-231 (PMC3560075; doi:10.1186/1471-2148-12-231)
Supplement: Additional file 1 — Supplemental notes 1–3. Detailed descriptions of the results, including the identification of SSTR sequences in genome databases as well as the phylogenetic analyses of neighboring gene families. [file 1471-2148-12-231-S1.pdf]

The evolution of vertebrate somatostatin receptors and their gene regions involves extensive chromosomal rearrangements

Daniel **Ocampo Daza***[1], Görel **Sundström**[1, 2], Christina A. **Bergqvist**[1] and Dan **Larhammar**[1]

[1] Department of Neuroscience, Uppsala University, Box 593, SE-75124 Uppsala, Sweden
[2] Present address: Department of Medical Biochemistry and Microbiology, Uppsala University, Box 582, SE-75123 Uppsala, Sweden
* Corresponding author
Telephone: +46-18-4714173
Fax: +46-18-511540

SUPPLEMENTAL NOTES

Supplemental note 1 - Identification of somatostatin receptor sequences in genome databases

Somatostatin receptor amino acid sequences were collected from the genome databases described in *Methods*. Many of the automatic predictions in the Ensembl database had to be revised manually in order to correct erroneous or incomplete exon predictions. The protein prediction error rates and types of error found in the Ensembl genome databases have been discussed by *Prosdocimi et al (2012)* [1]. A comprehensive list of the identified sequences, including chromosome locations, database identifiers and details on the revision of the sequence predictions, is provided in Additional file 3 (Table S4).

All five previously identified *SSTR* genes in mammals could be identified in the human, mouse, grey short-tailed opossum, chicken and Western clawed frog genomes. We could not identify *SSTR4* sequences in the genomes of the dog or anole lizard, nor in the National Center for Biotechnology Information (NCBI) Reference Sequence database for these species (Table 1). Rather than suggesting that the *SSTR4* genes have been lost from these species' genomes, their absence could be due to errors in the sequencing or assembly of these genomes.

SSTR2, -3 and *-5* sequences could be identified in all analyzed teleost genomes, as well as teleost-specific duplicates of *SSTR2*, *-3* and *-5* in many of them. The duplicates have been named *a* and *b* based on the phylogenetic analyses described below. There seem to have been differential losses of duplicates in some teleost genomes (see Table 1) as only *SSTR2* sequences were found in duplicate in all species. Alternatively, the differential absence of duplicates could reflect the incomplete status of some of the genome databases. An additional independent gain of an *SSTR3* gene seems to have occurred in the green spotted pufferfish genome and we have named this sequence *SSTR3c*. An *SSTR1* sequence could only be identified in the zebrafish, and no *SSTR4* sequences could be identified in any of the analyzed teleost genomes. The *SSTR6* sequences in zebrafish and stickleback have previously been wrongly annotated as *SSTR4* or *SSTR1* in the Ensembl genome browser. In the NCBI Gene database, the zebrafish *SSTR6* sequence is described as an SSTR1-like sequence (Gene ID: 557645).

Seven different *SSTR* sequences could be identified located on six different genomic scaffolds in the coelacanth genome database. In addition to the five previously known *SSTR* subtype sequences, an *SSTR6* sequence as well as a divergent *SSTR* sequence were found (see Table 1). The latter is located on the same genomic scaffold approximately 22 Kb downstream of the identified *SSTR2* gene prediction and in the same orientation. We have called this sequence *SSTRX* pending further analysis. It is encoded by an uninterrupted reading frame representing one exon with a predicted start codon and a stop codon followed by a predicted poly(A) sequence.

In the spotted gar genome *SSTR1, -2, -3* and *-5* sequences were identified, as well as a *SSTR6* sequence but no *SSTR4.* This mirrors the repertoire in the teleost fish genomes, but without duplicates of *SSTR2, -3* and *-5*.

Supplemental note 2 – Topologies of the SSTR1, -4 and -6-neighboring gene families

In total, our analysis identified 21 gene families showing a pattern of conserved synteny. Out of these, four were excluded upon preliminary phylogenetic analysis since their multitude of members made phylogenetic analyses unreliable, or because their topologies could not be resolved. The gene families included in our conserved synteny analysis of the *SSTR1, -4* and *-6* regions are presented in Table 2 and their phylogenetic trees are included in Additional file 5. Detailed information, including database identifiers and chromosomal locations of the identified gene family members, are included in Additional file 8 (Table S2). This includes information for those gene families that were excluded from the final analyses.

For eight of the 17 families - JAG, NIN, PYG, RALGAPA, RIN, SEC23, SLC24A and SPTLC - both the NJ trees and the PhyML trees display topologies that support an expansion in 2R (see Additional file 5, Figure S9, S10, S13, S14, S15, S16, S17 and S19 respectively). The topologies of most these families show an early vertebrate divergence of two to four well supported clusters (family members) including both tetrapod and teleost sequences. For the NIN family (see Additional file 5, Figure S10) no tunicate or amphioxus sequence could be identified to provide a more specific relative dating. For the SLC24A family both NJ and PhyML topologies support an expansion early in vertebrate evolution, although one of the subtype clusters consists of only teleost sequences and no fruit fly sequence could be identified. Instead, this family is rooted with an identified amphioxus sequence (see Additional file 5, Fig S17).

Of the remaining nine gene families, seven are in accordance with expansions in 2R, although there are some inconsistencies between the NJ and PhyML trees. The ABHD12, FLRT and ISM family NJ trees show well-supported subtype clusters including both tetrapod and teleost sequences diverging early in vertebrate evolution. However, the PhyML trees for these families show that one of the clusters in each family is unresolved (see Additional file 5, Figure S4, S6 and S8 respectively). Out of these, only the ABHD12 trees are rooted with identified fruit fly sequences (Table 2). Similarly the FOXA, NKX2, PAX and SNX NJ trees are consistent with an expansion in the time window of 2R, with well supported clusters including both tetrapod and teleost sequences; but in the PhyML trees the clustering of the tunicate sequences make the relative dating of the topologies unclear (see Additional file 5, Figure S7, S11, S12 and S18 respectively). Additionally both NKX2 and PAX trees show an unresolved teleost cluster each due to low bootstrap support in the PhyML analyses.

The FLRT, JAG, PYG, RIN and SLC24A gene families also show topologies that support the duplication of family members in the teleost-specific whole genome duplication 3R, using at least one of the phylogenetic methods. The ABHD, FOXA, NKX2 and SPTLC gene families also seem to have teleost duplicates, however the topologies of the teleost branches in both the NJ and PhyML trees are unclear.

Only two gene families, CFL and VSX, are inconclusive with regard to expansion in 2R (see Additional file 5, Figure S5 and S20): While both phylogenetic methods produce three main well-supported tetrapod branches, the overall topologies of the CFL trees remain largely unclear, with several unresolved teleost branches. The VSX gene family analyses are inconclusive using both phylogenetic methods, likely due to a combination of factors: This gene family consists of homeobox genes with a relatively short coding sequence and high degree of sequence conservation, and the identified amphioxus sequence is a fragment covering less that half of the alignment.

Supplemental note 3 – Topologies of the SSTR2, -3 and -5-neighboring gene families

Our analysis identified 43 gene families showing a pattern of conserved synteny. Out of these, 12 were excluded upon preliminary analysis using the same criteria as for the analysis of the *SSTR1, -4* and *-6* regions. Yet another identified gene family, the urotensin II receptor family (UTS2R), is currently being analyzed in closer detail in a separate study (work in progress) and is not included here due to numerous gene losses. The gene families included in our conserved synteny analysis of the *SSTR2, -3* and *-5* regions are presented in Table 3 and their phylogenetic trees are included in Additional file 6. As with the previous analysis of conserved synteny, detailed information can be found in Additional file 9 (Table S3).

For 23 of the 30 analyzed neighboring families both the NJ trees and the PhyML trees display topologies indicating that they arose by duplications of a single ancestral gene in the time window of 2R (see Additional file 6). Four additional families, ADAP, FAM20, RPH3A and TOM1, have some unresolved or contradictory branches in the NJ trees, but their PhyML trees support an expansion in 2R (see Additional file 6, Figure S21, S28, S42 and S47 respectively). Only three families, CABP, GGA and KCNJ, show unclear PhyML topologies. The topology of the PhyML tree for the CABP family has several branches with low bootstrap support (< 50%) and several of the clusters that are well supported in the NJ tree are unresolved in the PhyML tree (see Additional file 6, Figure S24). In the KCNJ PhyML tree the same is true for one of the subtype branches (see Additional file 6, Figure S34). In the PhyML tree for the GGA family the identified tunicate and amphioxus sequences branch together basal to one of the vertebrate subtype clusters, rather that basal to all the vertebrate sequences, which makes the relative dating of the expansion of this gene family inconclusive (see Additional file 6, Figure S31).

Out of the families that support or are consistent with duplications in 2R, the majority have topologies that show an early vertebrate divergence of two to four well supported clusters that include both tetrapod and teleost sequences. Some of them, however, have additional clusters or clusters where not all taxa are represented. The ATP2A family has one additional branch consisting of a zebrafish and a stickleback sequence (see Additional file 6, Figure S22) and the GLPR family has one additional subtype cluster consisting of both tetrapod and teleost sequences (see Additional file 6, Figure S32). These families likely went through additional duplication events; in the case of the GLPR family our relative dating suggest that these duplications took place early in vertebrate evolution. Our phylogenetic analyses of the GLPR family are consistent with a recent phylogenetic analysis [2], although the presented tree in that report did not include an invertebrate root. For both families these additional branches are well-supported in both the NJ and PhyML trees.

In the C1QTNF, FAM20 and RADIL families one of the subtype clusters lacks tetrapod sequences (see Additional file 6, Figure S23, S28 and S39 respectively). This probably represents paralogs that were generated in 2R but were subsequently lost in the tetrapod lineage. In the METRN and TEX2 families one of the subtype clusters shows losses of both tetrapod and teleost sequences. Nevertheless, both families are well-supported in the topologies of both the NJ and PhyML trees (see Additional file 6, Figure S36 and S45). For one family, SDK, no tunicate or amphioxus sequence could be identified to provide a more detailed relative dating (see Additional file 6, Figure S43).

Out of the 30 families that were analyzed, 24 also show teleost-specific expansions in at least one of the subtype clusters. For most of these families, the teleost-specific duplicate clusters are resolved and well-supported; although for the CYTH, FNG, FSCN, GGA, KCNJ, RADIL, SOX and TNRC6 families, individual teleost duplicate clusters are not clearly resolved due to the lack of sequences in some teleost genomes or due to high sequence

identity between the duplicates (see Additional file 6, Figure S27, S29, S30, S31, S39, S44 and S46 respectively).

This dataset also suggests that the ATP2A, CABP, GLPR and RPH3A gene families expanded as part of a different but partially overlapping paralogon. These families have members on some of the *SSTR2, -3* and *-5*-bearing chromosome regions, but we could also identify members on a seemingly separate set of chromosomal regions in all investigated genomes (see Additional file 4, Table S5). This is most clearly seen in the chicken genome where these families are the only ones we identified that have members on chromosomes 3, 15 and 19. Note that the ATP2A, CABP and GLPR families have unclear topologies, as described above. The analysis of the *Branchiostoma floridae* genome mentioned in the introduction [3] suggests that these families belong in a separate, but partially overlapping, paralogon. Therefore, we choose not to draw conclusions about these additional chromosomal regions.

ADDITIONAL REFERENCES

1. Prosdocimi F, Linard B, Pontarotti P, Poch O, Thompson JD: **Controversies in modern evolutionary biology: the imperative for error detection and quality control.** *BMC genomics* 2012, **13**:510.1186/1471-2164-13-5.

2. Irwin DM, Prentice KJ: **Incretin hormones and the expanding families of glucagon-like sequences and their receptors.** *Diabetes, obesity & metabolism* 2011, **13 Suppl 1**:69–8110.1111/j.1463-1326.2011.01444.x.

3. Putnam NH, Butts T, Ferrier DEK, Furlong RF, Hellsten U, Kawashima T, Robinson-Rechavi M, Shoguchi E, Terry A, Yu J-K, Benito-Gutiérrez E, Dubchak I, Garcia-Fernàndez J, Gibson-Brown JJ, Grigoriev IV, Horton AC, de Jong PJ, Jurka J, Kapitonov VV, Kohara Y, Kuroki Y, Lindquist E, Lucas S, Osoegawa K, Pennacchio LA, Salamov AA, Satou Y, Sauka-Spengler T, Schmutz J, Shin-I T, et al.: **The amphioxus genome and the evolution of the chordate karyotype.** *Nature* 2008, **453**:1064–7110.1038/nature06967.
