# Supplementary material for: The evolution of vertebrate somatostatin receptors and their gene regions involves extensive chromosomal rearrangements
Source: BMC Evol Biol. 2012 Nov 29;12:231. doi: 10.1186/1471-2148-12-231 (PMC3560075; doi:10.1186/1471-2148-12-231)
Supplement: Additional file 2 — Figures S1–S3. All phylogenetic analyses of the SSTR gene family. [file 1471-2148-12-231-S2.pdf]

The evolution of vertebrate somatostatin receptors and their gene regions involves extensive chromosomal rearrangements

Daniel **Ocampo Daza***[1], Görel **Sundström**[1, 2], Christina A. **Bergqvist**[1] and Dan **Larhammar**[1]

[1] Department of Neuroscience, Uppsala University, Box 593, SE-75124 Uppsala, Sweden
[2] Present address: Department of Medical Biochemistry and Microbiology, Uppsala University, Box 582, SE-75123 Uppsala, Sweden
* Corresponding author
Telephone: +46-18-4714173
Fax: +46-18-511540

SUPPLEMENTAL FIGURES 1-3

Figure S1: Bootstrapped phylogenetic maximum likelihood tree of the SSTR gene family. The topology of the tree is supported by a non-parametric bootstrap analysis with 100 replicates (see Methods). Branch support is shown at the nodes. The tree is rooted with the human kisspeptin receptor 1 sequence (not shown). Comprehensive information about the sequences is available in Additional file 7, Table S1.

Figure S2: aLRT-supported phylogenetic maximum likelihood tree of the SSTR gene family. The topology of the tree is supported by an SH-like approximate likelihood ratio test (see Methods). Branch support is shown at the nodes. The tree is rooted with the human kisspeptin receptor 1 sequence (not shown). Comprehensive information about the sequences is available in Additional file 7, Table S1.

Figure S3: Identification of additional known teleost SSTR sequences. Additional teleost sequences from goldfish, grass carp, orange-spotted grouper, black ghost knifefish and rainbow trout (see Discussion) were added to the sequence alignment described in our results. The NCBI accession IDs of these sequences can be seen in the sequence names (in colors) and in Additional file 7, Table S1. The tree was made using the neighbor joining method supported by 100 bootstrap replicates (see Methods). Branch support is shown at the nodes. The tree supports the hypothesis that both the goldfish and rainbow trout have retained duplicates genes from the respective 4R whole genome duplications that occurred in their individual lineages. The goldfish has 4R duplicates of *SSTR1* (GenBank:AAF08613.1 and GenBank:AAF08614.1) and *SSTR5a* (GenBank:AAN76495.1 and GenBank:AAM18805.1), and the rainbow trout of *SSTR6* (GenBank:NP_001118006.1 and GenBank:NP_001118115.1, misidentified as *SSTR1a* and *–b* in the literature) and *SSTR2a* (GenBank:ADH59744.1 and NP_001182111.1).

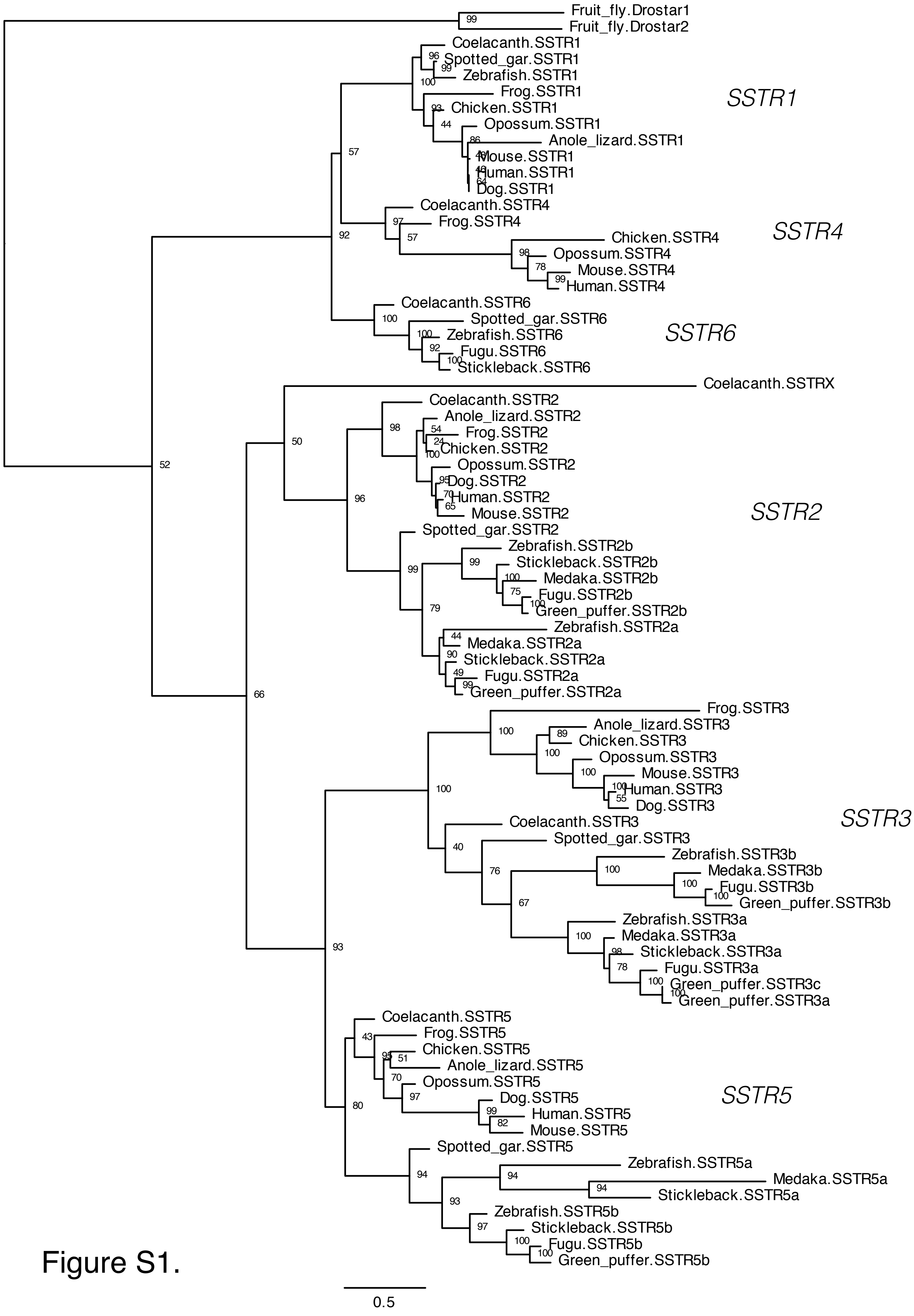
Fruit_fly.Drostar1
Fruit_fly.Drostar2
Coelacanth.SSTR1
Spotted_gar.SSTR1
Zebrafish.SSTR1
Frog.SSTR1
Chicken.SSTR1
Opossum.SSTR1
Anole_lizard.SSTR1
Mouse.SSTR1
Human.SSTR1
Dog.SSTR1
SSTR1
Coelacanth.SSTR4
Frog.SSTR4
Chicken.SSTR4
Opossum.SSTR4
Mouse.SSTR4
Human.SSTR4
SSTR4
Coelacanth.SSTR6
Spotted_gar.SSTR6
Zebrafish.SSTR6
Fugu.SSTR6
Stickleback.SSTR6
SSTR6
Coelacanth.SSTRX
Coelacanth.SSTR2
Anole_lizard.SSTR2
Frog.SSTR2
Chicken.SSTR2
Opossum.SSTR2
Dog.SSTR2
Human.SSTR2
Mouse.SSTR2
SSTR2
Spotted_gar.SSTR2
Zebrafish.SSTR2b
Stickleback.SSTR2b
Medaka.SSTR2b
Fugu.SSTR2b
Green_puffer.SSTR2b
Zebrafish.SSTR2a
Medaka.SSTR2a
Stickleback.SSTR2a
Fugu.SSTR2a
Green_puffer.SSTR2a
Frog.SSTR3
Anole_lizard.SSTR3
Chicken.SSTR3
Opossum.SSTR3
Mouse.SSTR3
Human.SSTR3
Dog.SSTR3
Coelacanth.SSTR3
SSTR3
Spotted_gar.SSTR3
Zebrafish.SSTR3b
Medaka.SSTR3b
Fugu.SSTR3b
Green_puffer.SSTR3b
Zebrafish.SSTR3a
Medaka.SSTR3a
Stickleback.SSTR3a
Fugu.SSTR3a
Green_puffer.SSTR3c
Green_puffer.SSTR3a
Coelacanth.SSTR5
Frog.SSTR5
Chicken.SSTR5
Anole_lizard.SSTR5
Opossum.SSTR5
SSTR5
Dog.SSTR5
Human.SSTR5
Mouse.SSTR5
Spotted_gar.SSTR5
Zebrafish.SSTR5a
Medaka.SSTR5a
Stickleback.SSTR5a
Zebrafish.SSTR5b
Stickleback.SSTR5b
Fugu.SSTR5b
Green_puffer.SSTR5b
0.5

Figure S1.

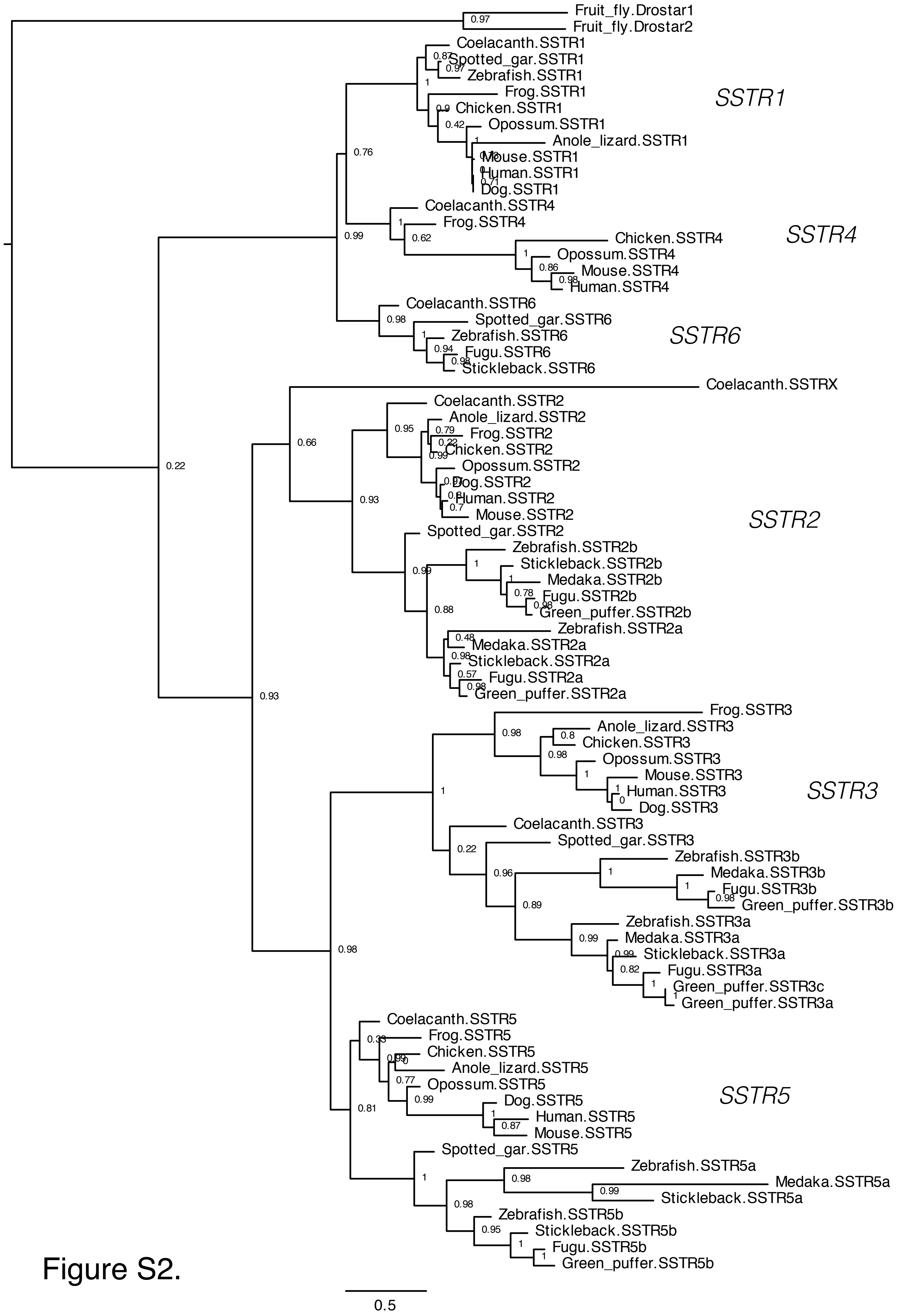
Fruit_fly.Drostar1
Fruit_fly.Drostar2
Coelacanth.SSTR1
Spotted_gar.SSTR1
Zebrafish.SSTR1
Frog.SSTR1
Chicken.SSTR1
Opossum.SSTR1
Anole_lizard.SSTR1
Mouse.SSTR1
Human.SSTR1
Dog.SSTR1
SSTR1
Coelacanth.SSTR4
Frog.SSTR4
Chicken.SSTR4
Opossum.SSTR4
Mouse.SSTR4
Human.SSTR4
SSTR4
Coelacanth.SSTR6
Spotted_gar.SSTR6
Zebrafish.SSTR6
Fugu.SSTR6
Stickleback.SSTR6
SSTR6
Coelacanth.SSTRX
Coelacanth.SSTR2
Anole_lizard.SSTR2
Frog.SSTR2
Chicken.SSTR2
Opossum.SSTR2
Dog.SSTR2
Human.SSTR2
Mouse.SSTR2
SSTR2
Spotted_gar.SSTR2
Zebrafish.SSTR2b
Stickleback.SSTR2b
Medaka.SSTR2b
Fugu.SSTR2b
Green_puffer.SSTR2b
Zebrafish.SSTR2a
Medaka.SSTR2a
Stickleback.SSTR2a
Fugu.SSTR2a
Green_puffer.SSTR2a
Frog.SSTR3
Anole_lizard.SSTR3
Chicken.SSTR3
Opossum.SSTR3
Mouse.SSTR3
Human.SSTR3
Dog.SSTR3
SSTR3
Coelacanth.SSTR3
Spotted_gar.SSTR3
Zebrafish.SSTR3b
Medaka.SSTR3b
Fugu.SSTR3b
Green_puffer.SSTR3b
Zebrafish.SSTR3a
Medaka.SSTR3a
Stickleback.SSTR3a
Fugu.SSTR3a
Green_puffer.SSTR3c
Green_puffer.SSTR3a
Coelacanth.SSTR5
Frog.SSTR5
Chicken.SSTR5
Anole_lizard.SSTR5
Opossum.SSTR5
Dog.SSTR5
Human.SSTR5
Mouse.SSTR5
SSTR5
Spotted_gar.SSTR5
Zebrafish.SSTR5a
Medaka.SSTR5a
Stickleback.SSTR5a
Zebrafish.SSTR5b
Stickleback.SSTR5b
Fugu.SSTR5b
Green_puffer.SSTR5b
0.5

Figure S2.

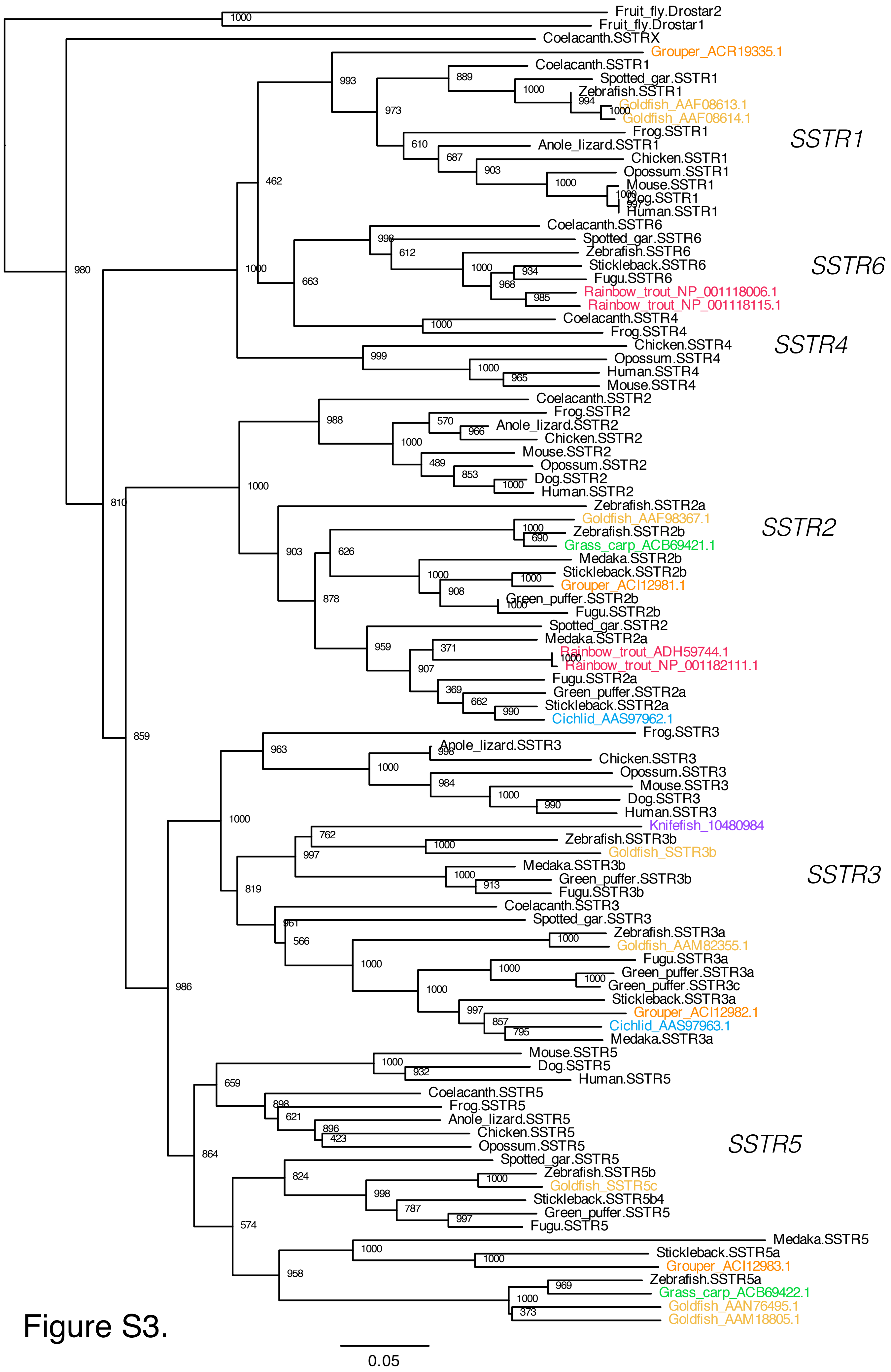
Fruit_fly.Drostar2
Fruit_fly.Drostar1
Coelacanth.SSTRX
Grouper_ACR19335.1
Coelacanth.SSTR1
Spotted_gar.SSTR1
Zebrafish.SSTR1
Goldfish_AAF08613.1
Goldfish_AAF08614.1
Frog.SSTR1
Anole_lizard.SSTR1
Chicken.SSTR1
Opossum.SSTR1
Mouse.SSTR1
Dog.SSTR1
Human.SSTR1
SSTR1
Coelacanth.SSTR6
Spotted_gar.SSTR6
Zebrafish.SSTR6
Stickleback.SSTR6
Fugu.SSTR6
Rainbow_trout_NP_001118006.1
Rainbow_trout_NP_001118115.1
SSTR6
Coelacanth.SSTR4
Frog.SSTR4
Chicken.SSTR4
Opossum.SSTR4
Human.SSTR4
Mouse.SSTR4
SSTR4
Coelacanth.SSTR2
Frog.SSTR2
Anole_lizard.SSTR2
Chicken.SSTR2
Mouse.SSTR2
Opossum.SSTR2
Dog.SSTR2
Human.SSTR2
Zebrafish.SSTR2a
Goldfish_AAF98367.1
Zebrafish.SSTR2b
Grass_carp_ACB69421.1
Medaka.SSTR2b
Stickleback.SSTR2b
Grouper_ACI12981.1
Green_puffer.SSTR2b
Fugu.SSTR2b
Spotted_gar.SSTR2
Medaka.SSTR2a
Rainbow_trout_ADH59744.1
Rainbow_trout_NP_001182111.1
Fugu.SSTR2a
Green_puffer.SSTR2a
Stickleback.SSTR2a
Cichlid_AAS97962.1
SSTR2
Frog.SSTR3
Anole_lizard.SSTR3
Chicken.SSTR3
Opossum.SSTR3
Mouse.SSTR3
Dog.SSTR3
Human.SSTR3
Knifefish_10480984
Zebrafish.SSTR3b
Goldfish_SSTR3b
Medaka.SSTR3b
Green_puffer.SSTR3b
Fugu.SSTR3b
Coelacanth.SSTR3
Spotted_gar.SSTR3
Zebrafish.SSTR3a
Goldfish_AAM82355.1
Fugu.SSTR3a
Green_puffer.SSTR3a
Green_puffer.SSTR3c
Stickleback.SSTR3a
Grouper_ACI12982.1
Cichlid_AAS97963.1
Medaka.SSTR3a
SSTR3
Mouse.SSTR5
Dog.SSTR5
Human.SSTR5
Coelacanth.SSTR5
Frog.SSTR5
Anole_lizard.SSTR5
Chicken.SSTR5
Opossum.SSTR5
Spotted_gar.SSTR5
Zebrafish.SSTR5b
Goldfish_SSTR5c
Stickleback.SSTR5b4
Green_puffer.SSTR5
Fugu.SSTR5
Medaka.SSTR5
Stickleback.SSTR5a
Grouper_ACI12983.1
Zebrafish.SSTR5a
Grass_carp_ACB69422.1
Goldfish_AAN76495.1
Goldfish_AAM18805.1
SSTR5
0.05

Figure S3.
