# Supplementary material for: The evolution of vertebrate somatostatin receptors and their gene regions involves extensive chromosomal rearrangements
Source: BMC Evol Biol. 2012 Nov 29;12:231. doi: 10.1186/1471-2148-12-231 (PMC3560075; doi:10.1186/1471-2148-12-231)
Supplement: Additional file 5 — Figures S4–S20. Phylogenetic trees of the SSTR1, -4 and -6-neighboring gene families. Figures are numbered S4-S20 and include both neighbor joining and phylogenetic maximum likelihood trees of the gene families described in Table 2. [file 1471-2148-12-231-S5.pdf]

The evolution of vertebrate somatostatin receptors and their gene regions involves extensive chromosomal rearrangements

Daniel **Ocampo Daza***[1], Görel **Sundström**[1, 2], Christina A. **Bergqvist**[1] and Dan **Larhammar**[1]

[1] Department of Neuroscience, Uppsala University, Box 593, SE-75124 Uppsala, Sweden
[2] Present address: Department of Medical Biochemistry and Microbiology, Uppsala University, Box 582, SE-75123 Uppsala, Sweden
* Corresponding author
Telephone: +46-18-4714173
Fax: +46-18-511540

SUPPLEMENTAL FIGURES 4-20

Figures S4-20: Bootstrapped Neighbor Joining (NJ) and Phylogenetic Maximum Likelihood (PhyML) trees of the *SSTR1, -4* and *-6*-neighboring gene families. The topologies are supported by non-parametric bootstrap analyses with 1000 (NJ) or 100 (PhyML) replicates (see Methods). Branch support is shown at the nodes. The trees are rooted with the identified fruit fly sequences, unless specified in Table 2. Species abbreviations are applied as follows: *Homo sapiens* (Hsa), *Mus musculus* (Mmu), *Canis familiaris* (Cfa), *Monodelphis domestica* (Mdo), *Macropus eugenii* (Meu), *Gallus gallus* (Gga), *Meleagris gallopavo* (Mga), *Taeniopygia guttata* (Tgu), *Anolis carolinensis* (Aca), *Silurana (Xenopus) tropicalis* (Xtr), *Danio rerio* (Dre), *Oryzias latipes* (Ola), *Gasterosteus aculeatus* (Gac), *Tetraodon nigroviridis* (Tni), *Takifugu rubripes* (Tru), *Ciona intestinalis* (Cin), *Ciona savignyi* (Csa), *Branchiostoma floridae* (Bfl), *Caenorhabditis elegans* (Cel) and *Drosophila melanogaster* (Dme). See Methods – "*Identification and analysis of neighboring gene families/Conserved synteny analysis*" - for the choice of species representation. HGNC symbols are used for all human sequences. Other species' sequences are identified by chromosomal/genomic scaffold assignment. Lowercase a, b and c are used to distinguish sequences located on the same chromosomes. Comprehensive information about each sequence is available in Additional file 8, Table S2.

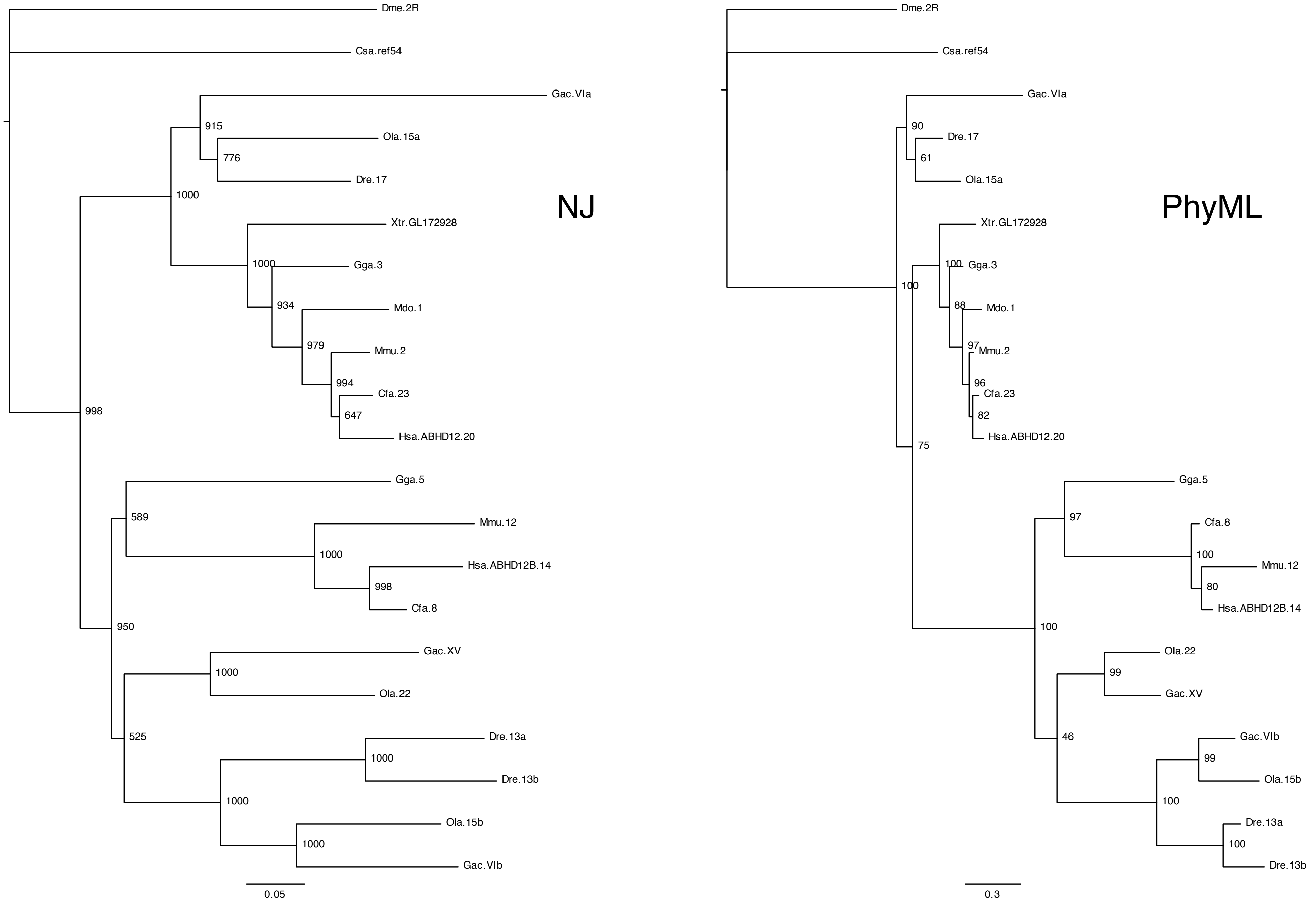

Figure S4. ABHD12

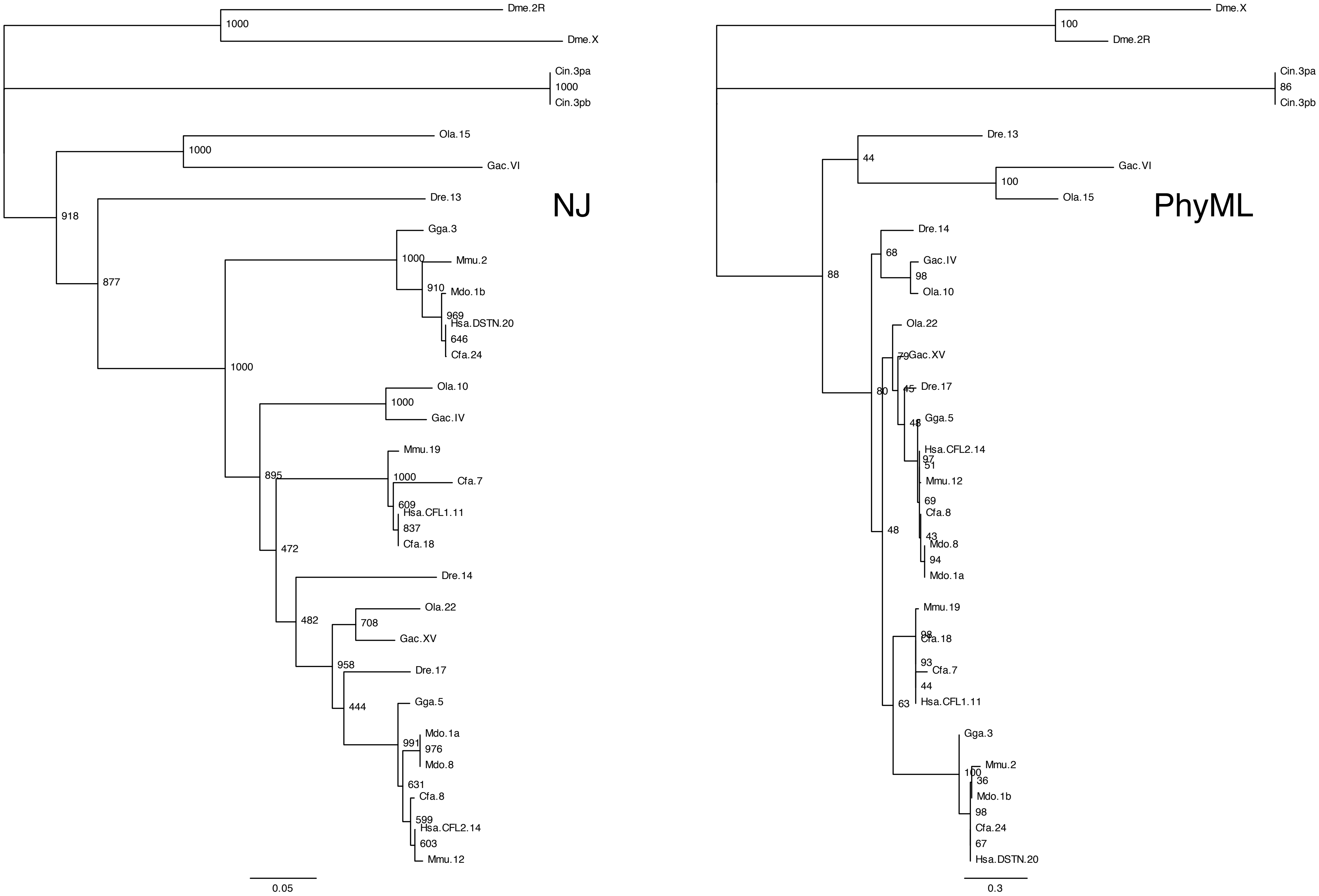

Figure S5. CFL

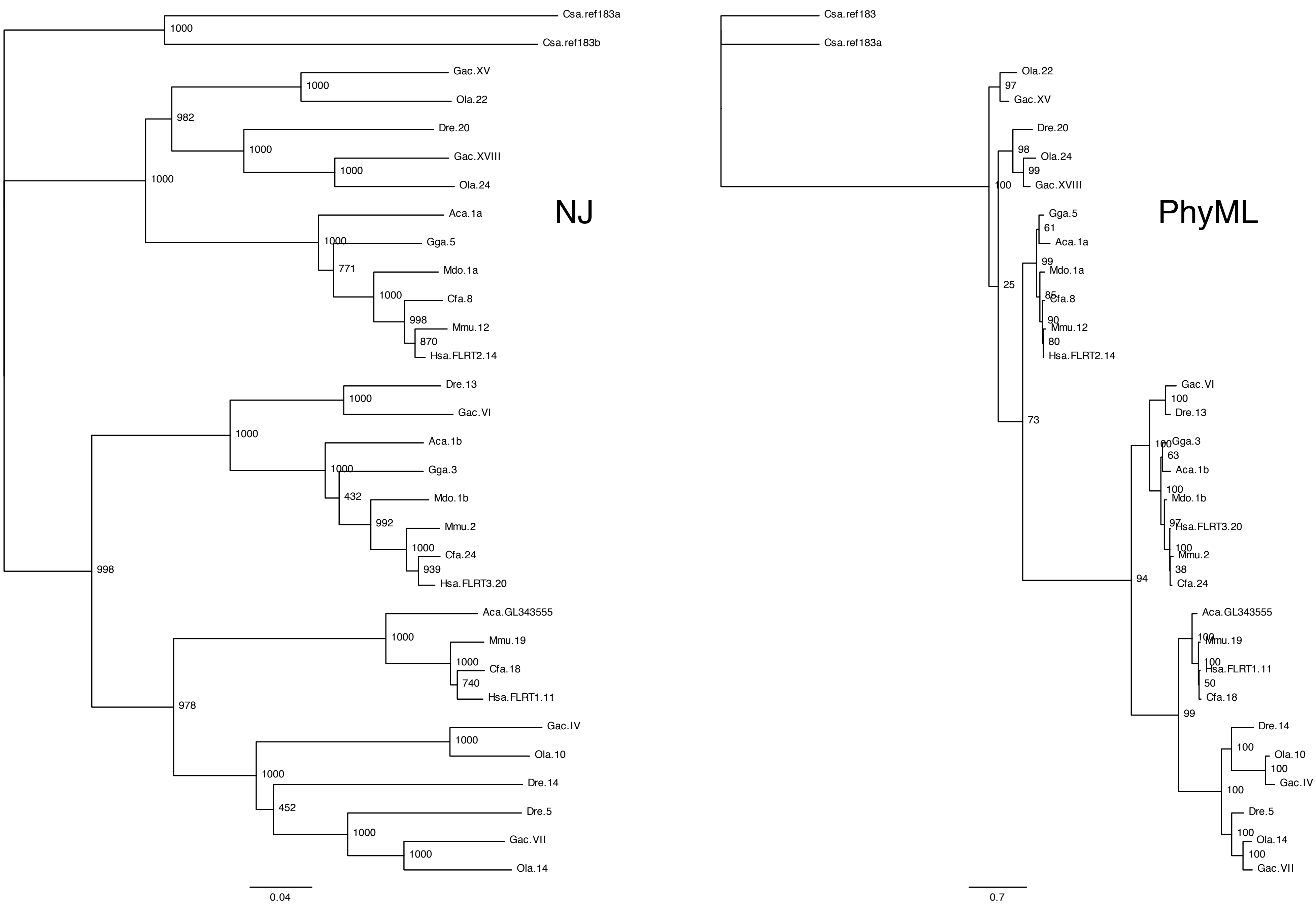

Figure S6. FLRT

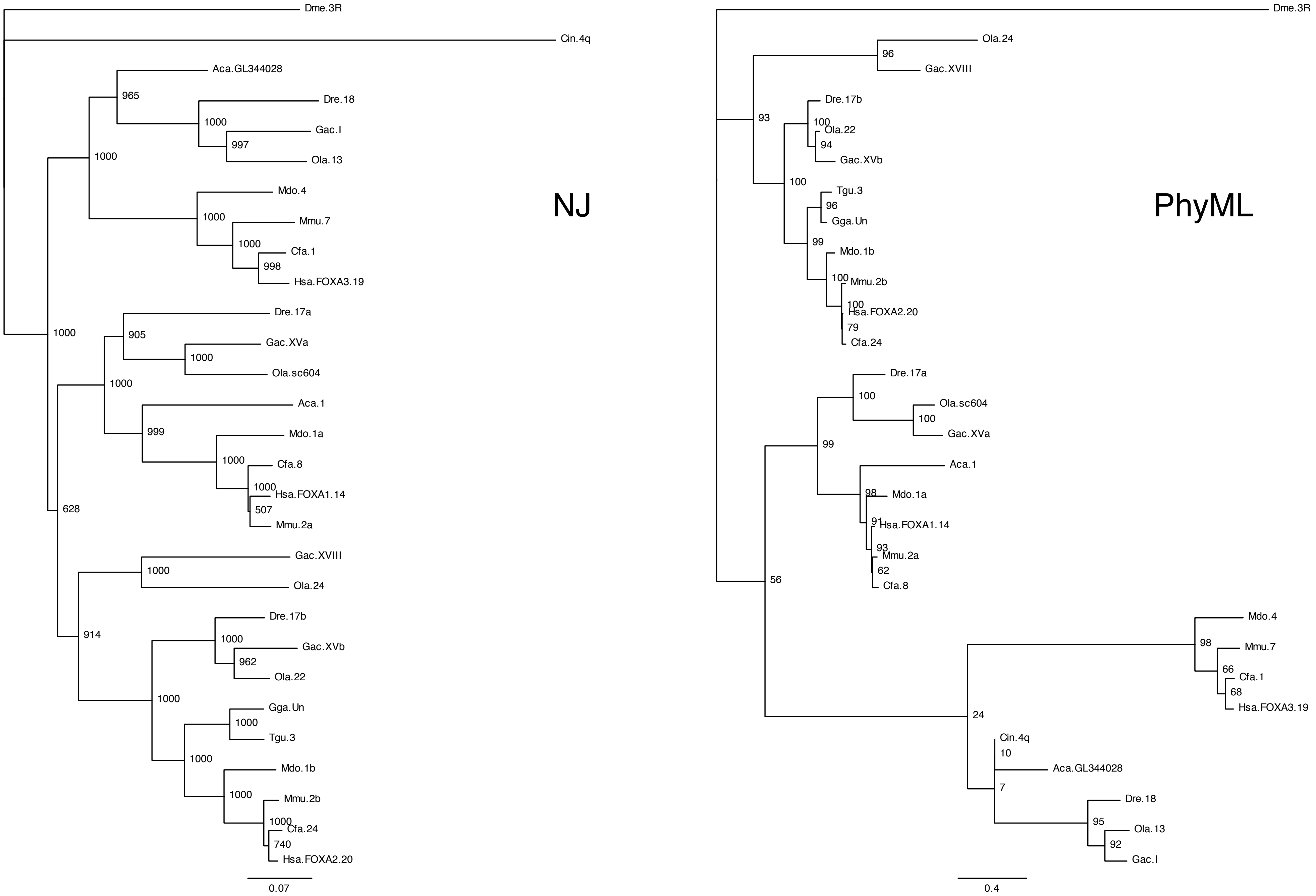

Figure S7. FOXA

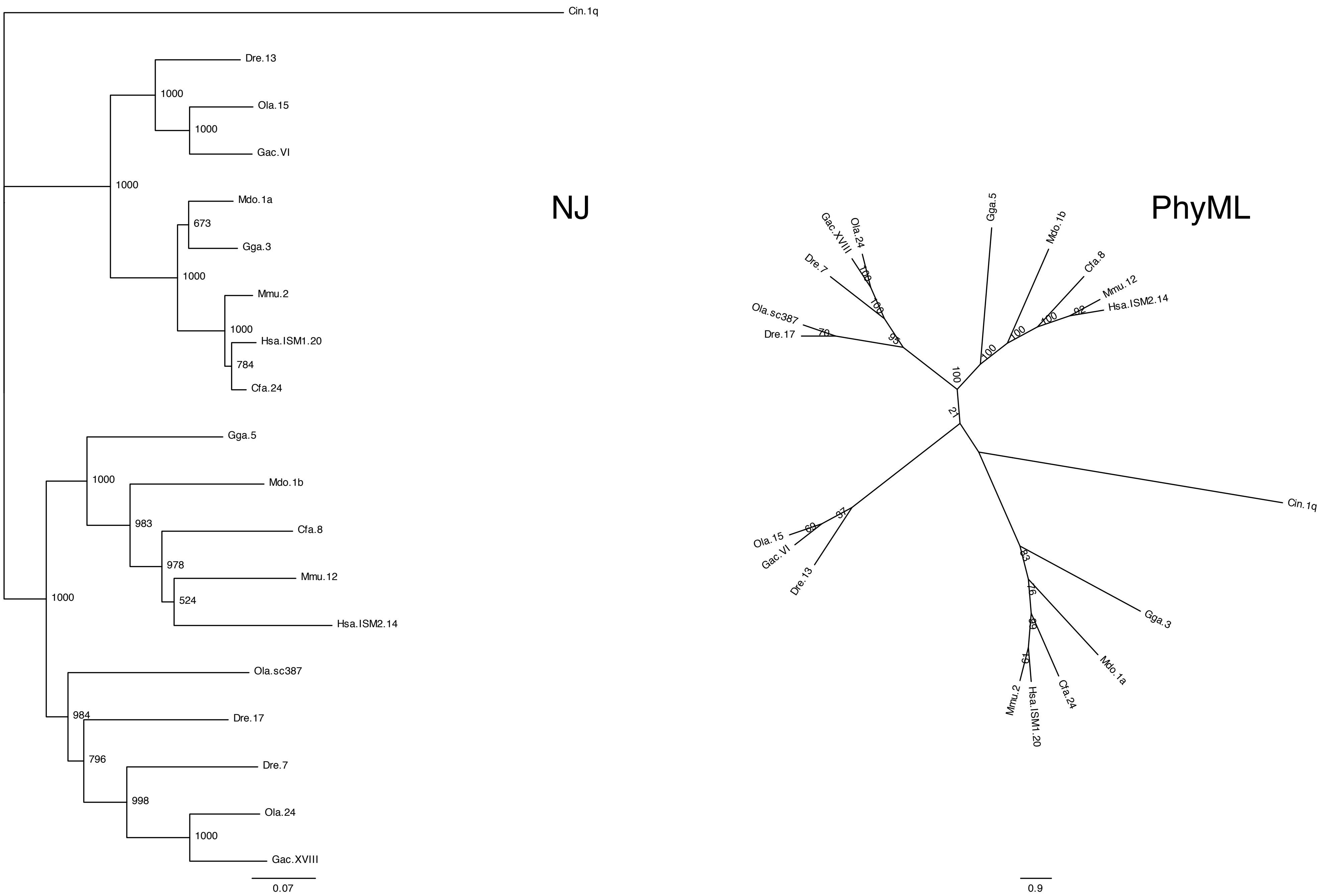

Figure S8. ISM

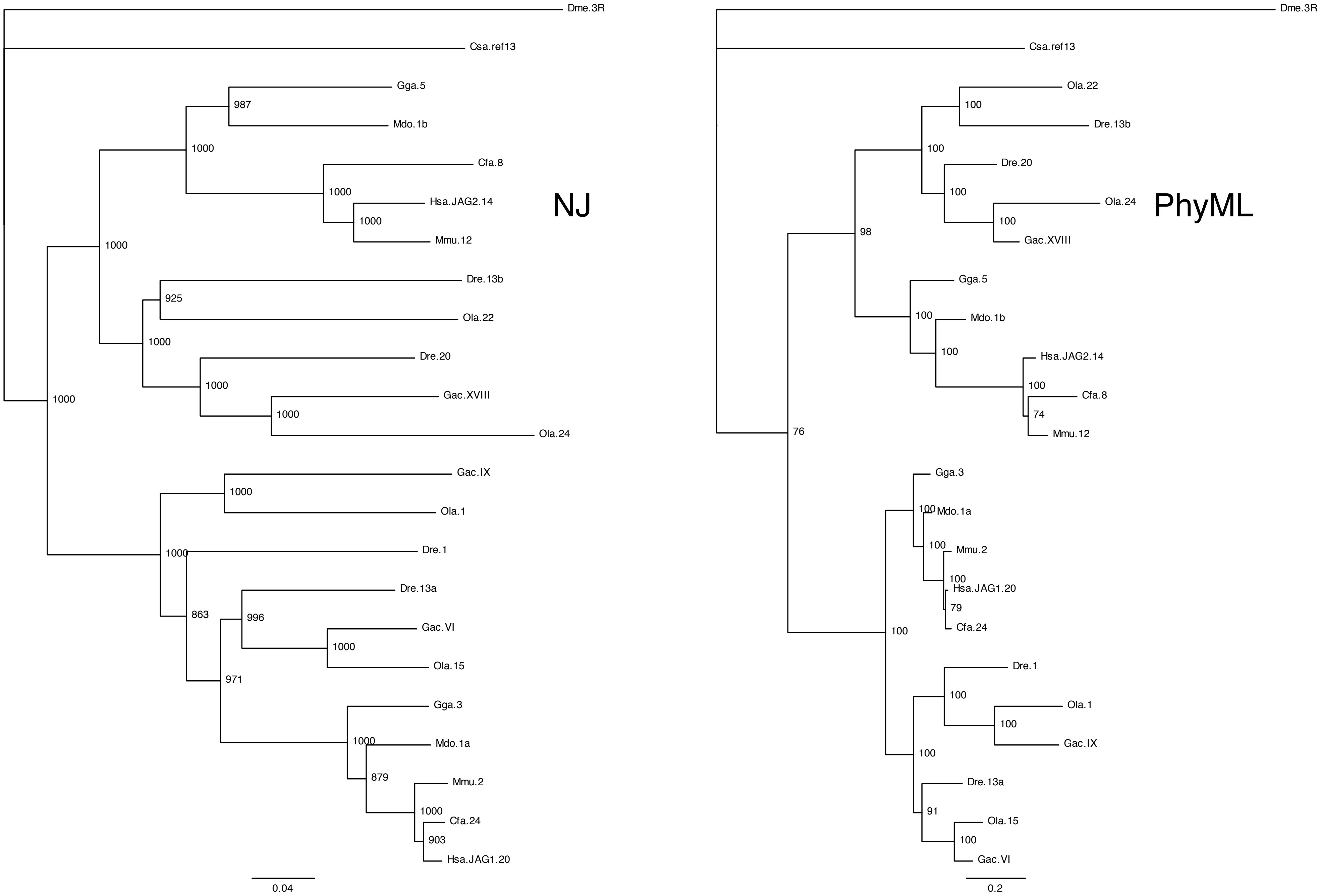

Figure S9. JAG

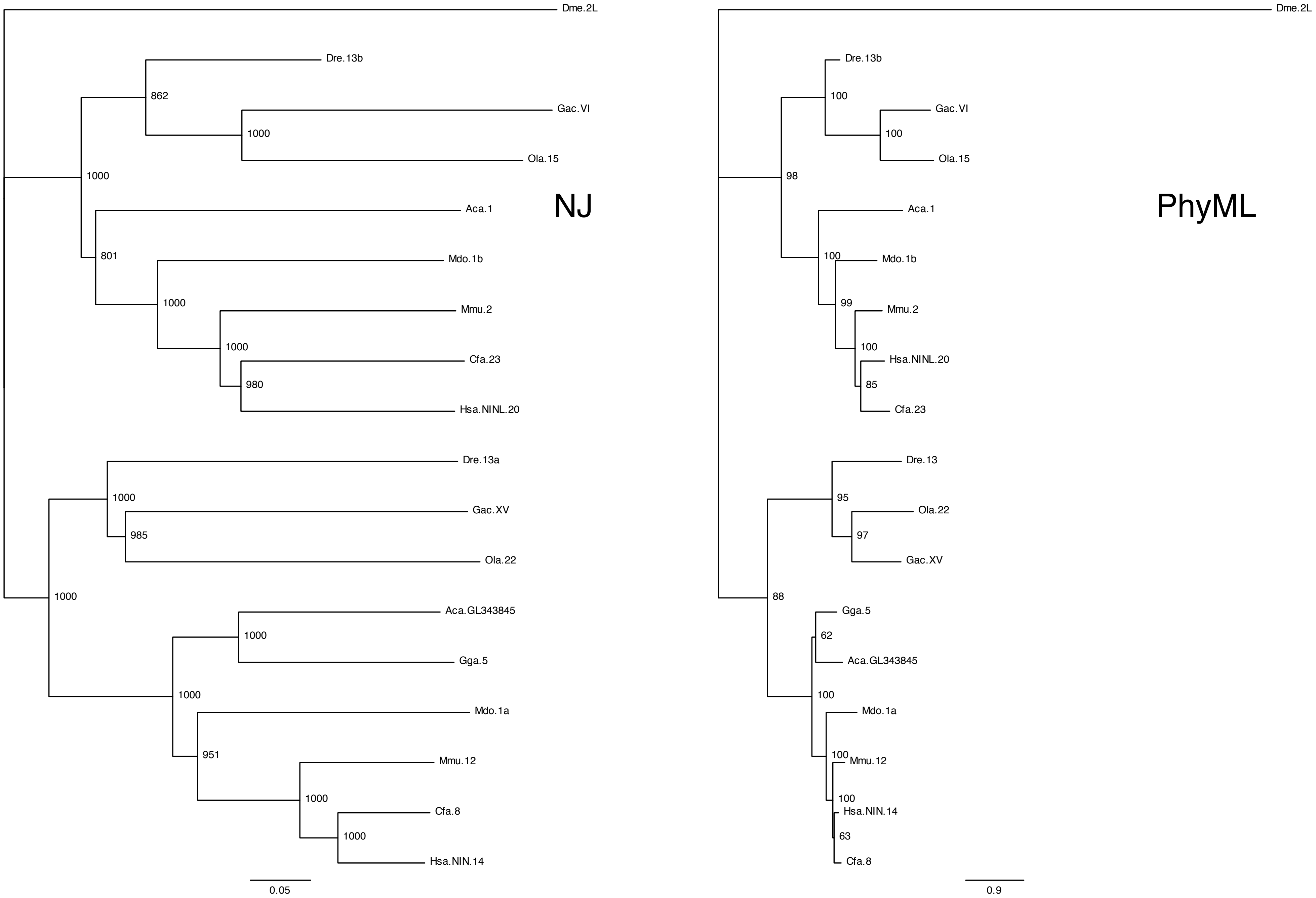

Figure S10. NIN

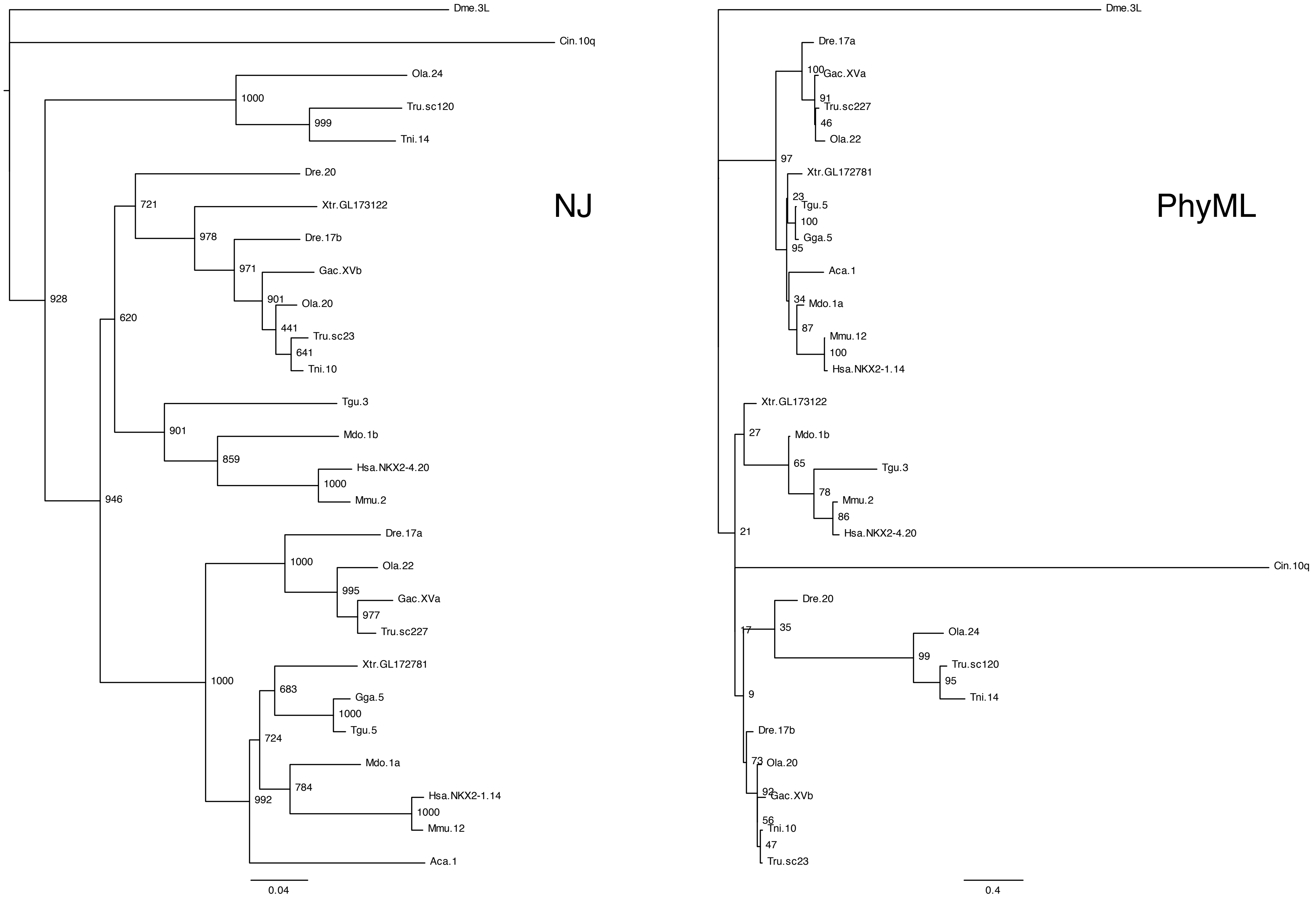

Figure S11. NKX2

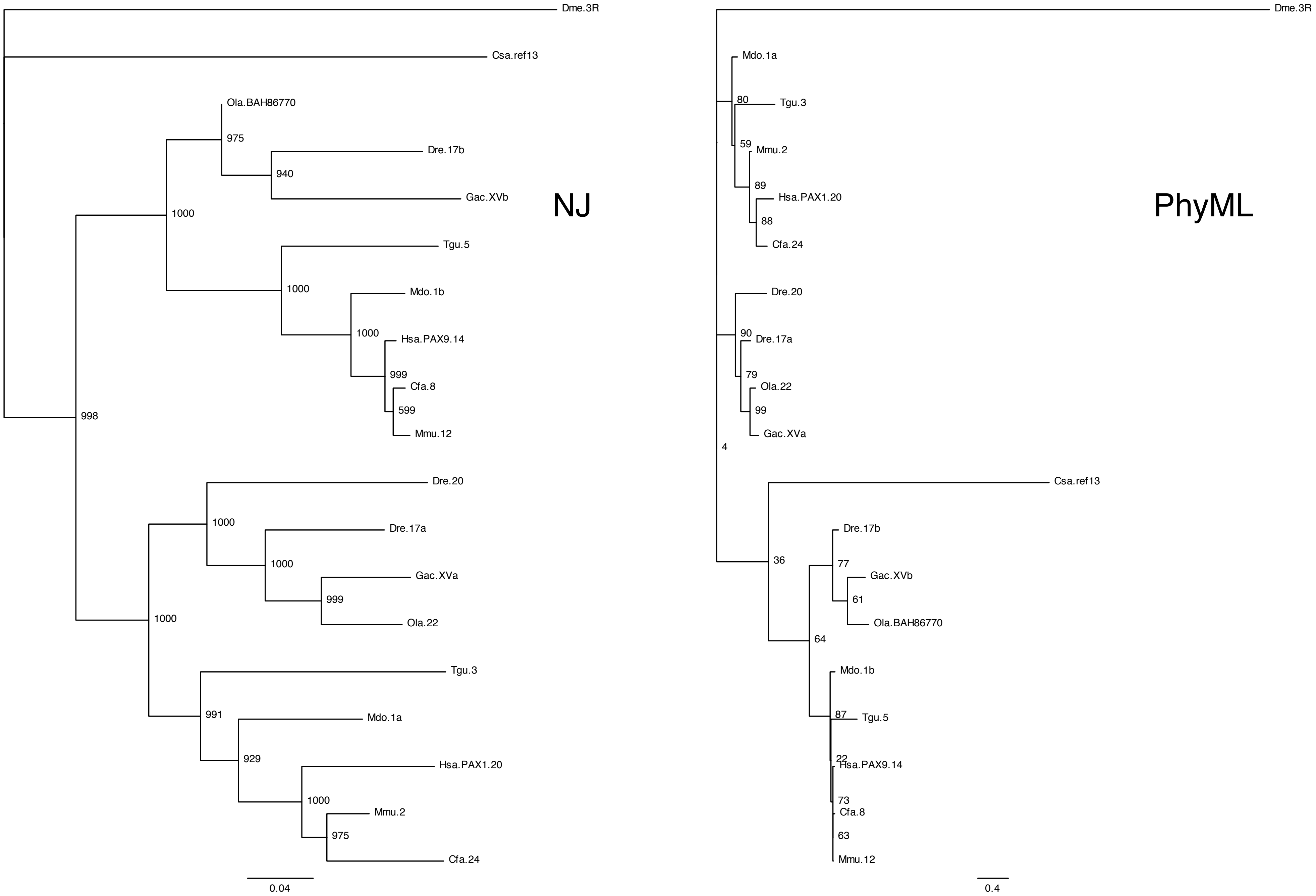

Figure S12. PAX

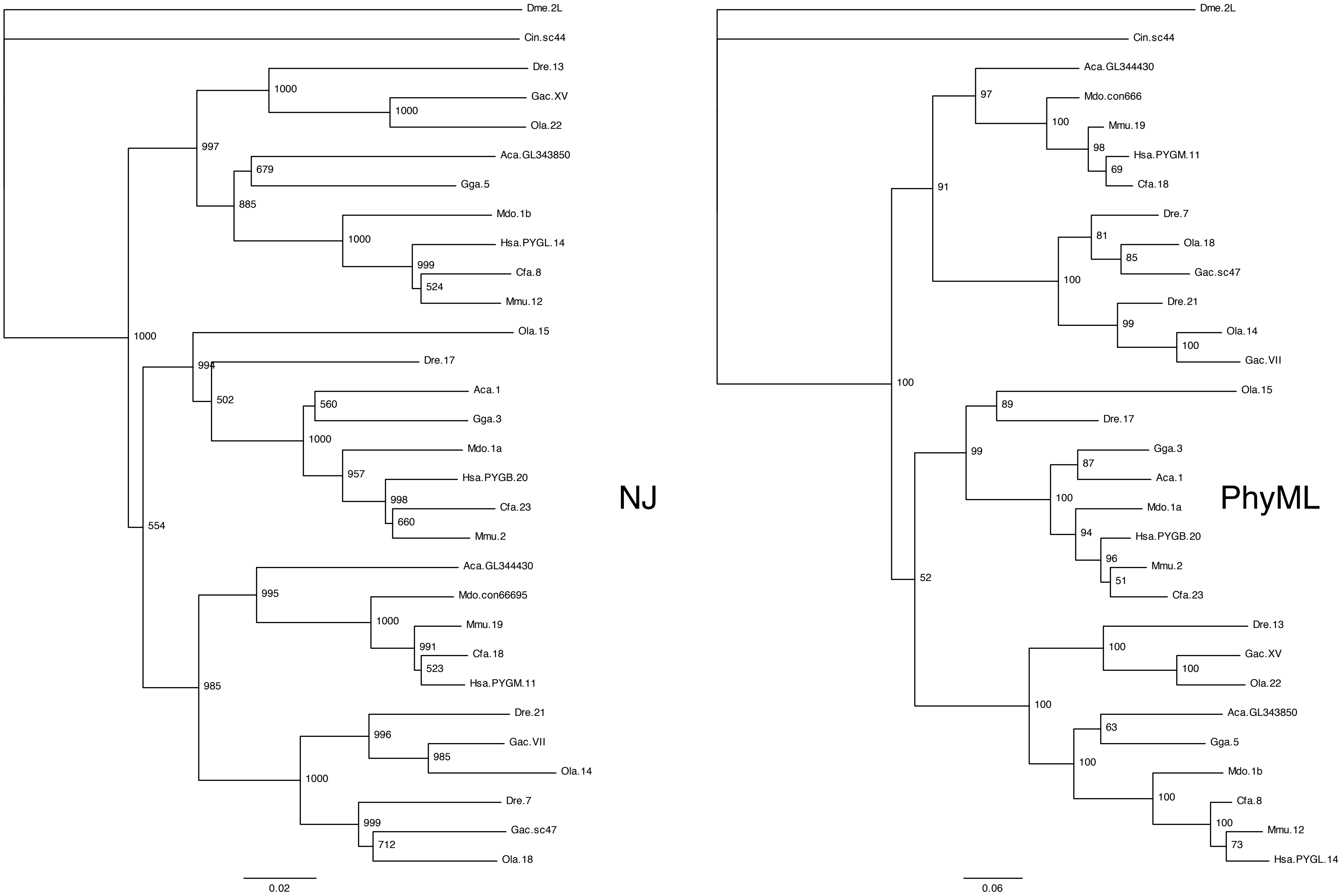

Figure S13. PYG

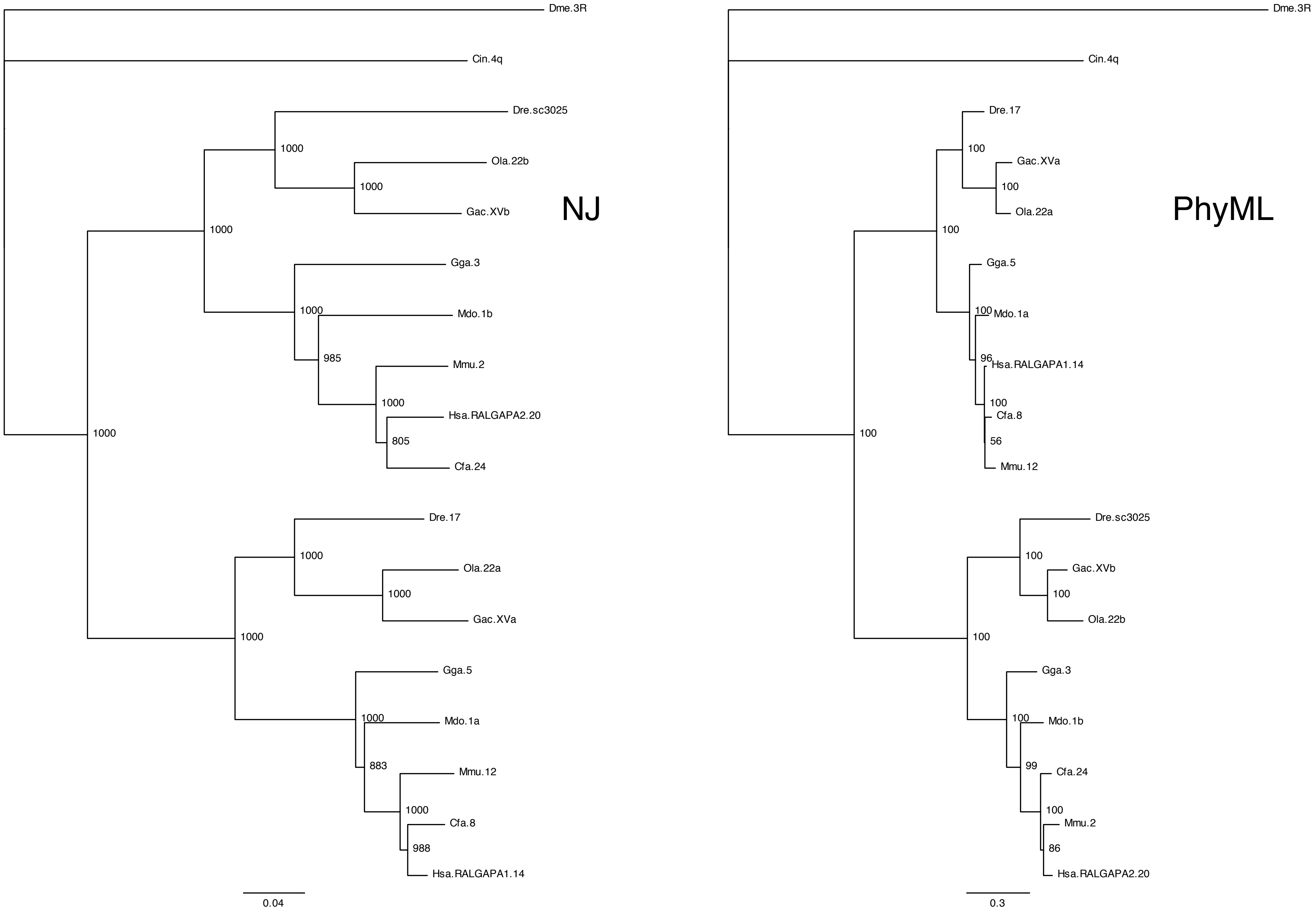

Figure S14. RALGAPA

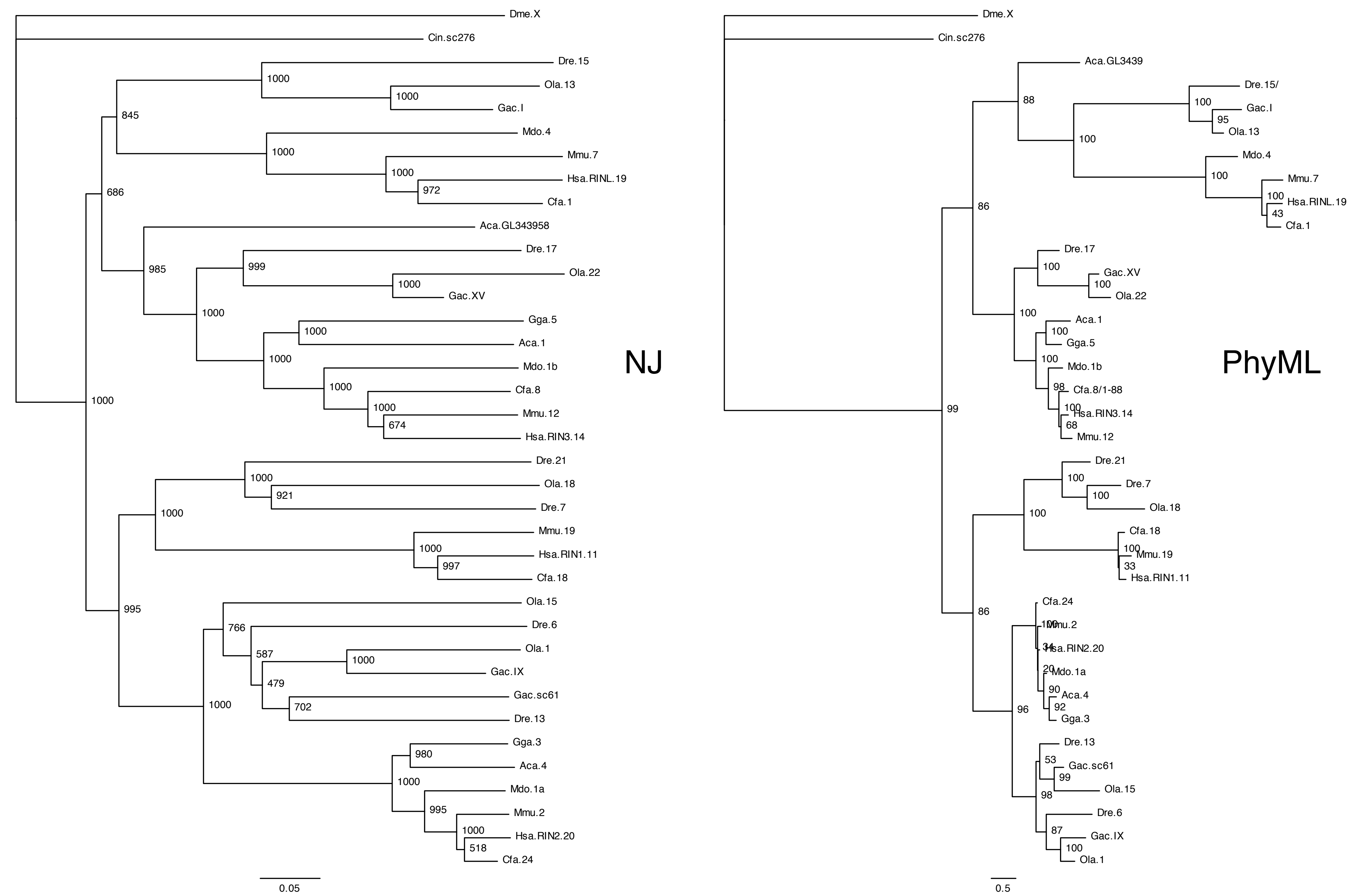

Figure S15. RIN

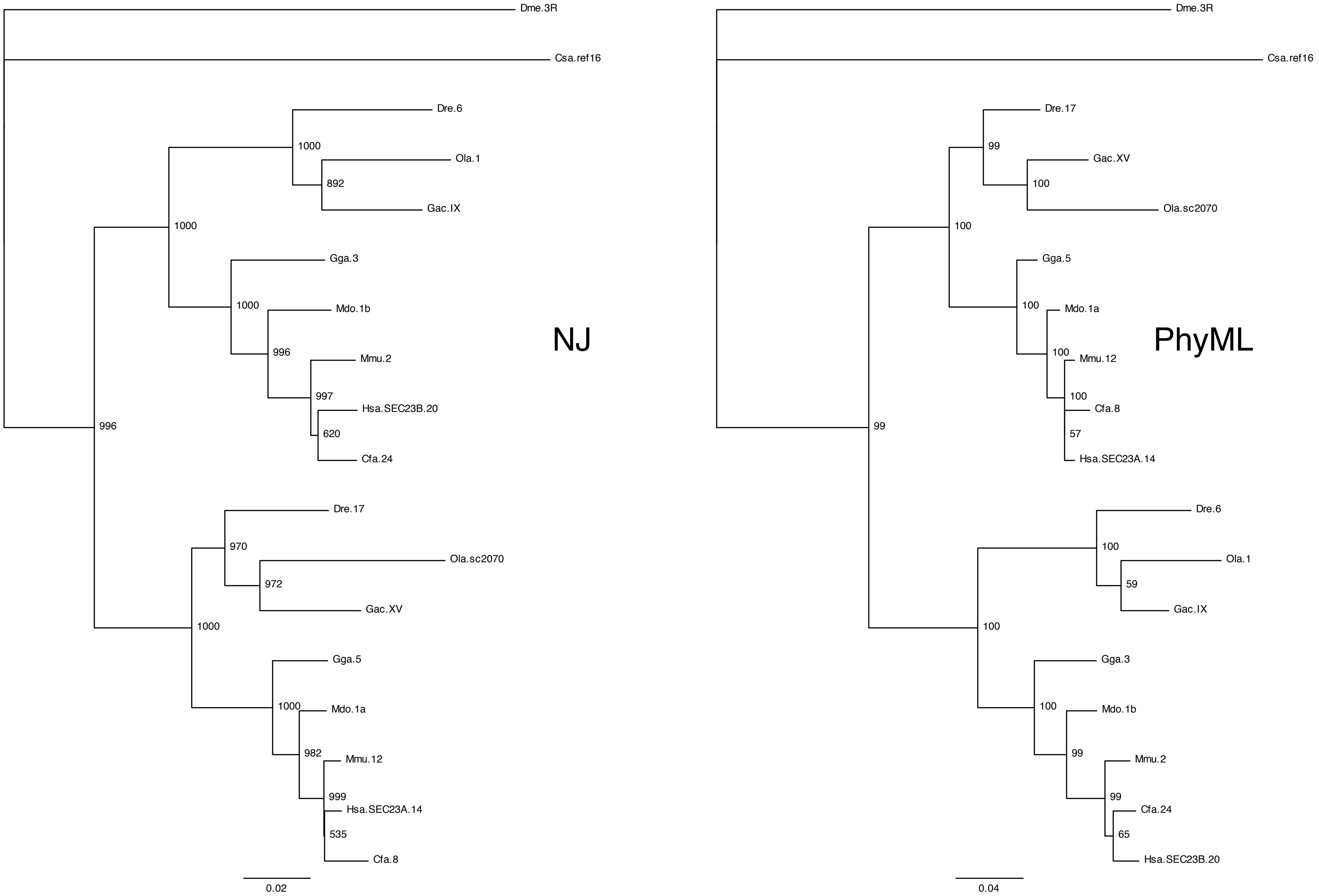

Figure S16. SEC23

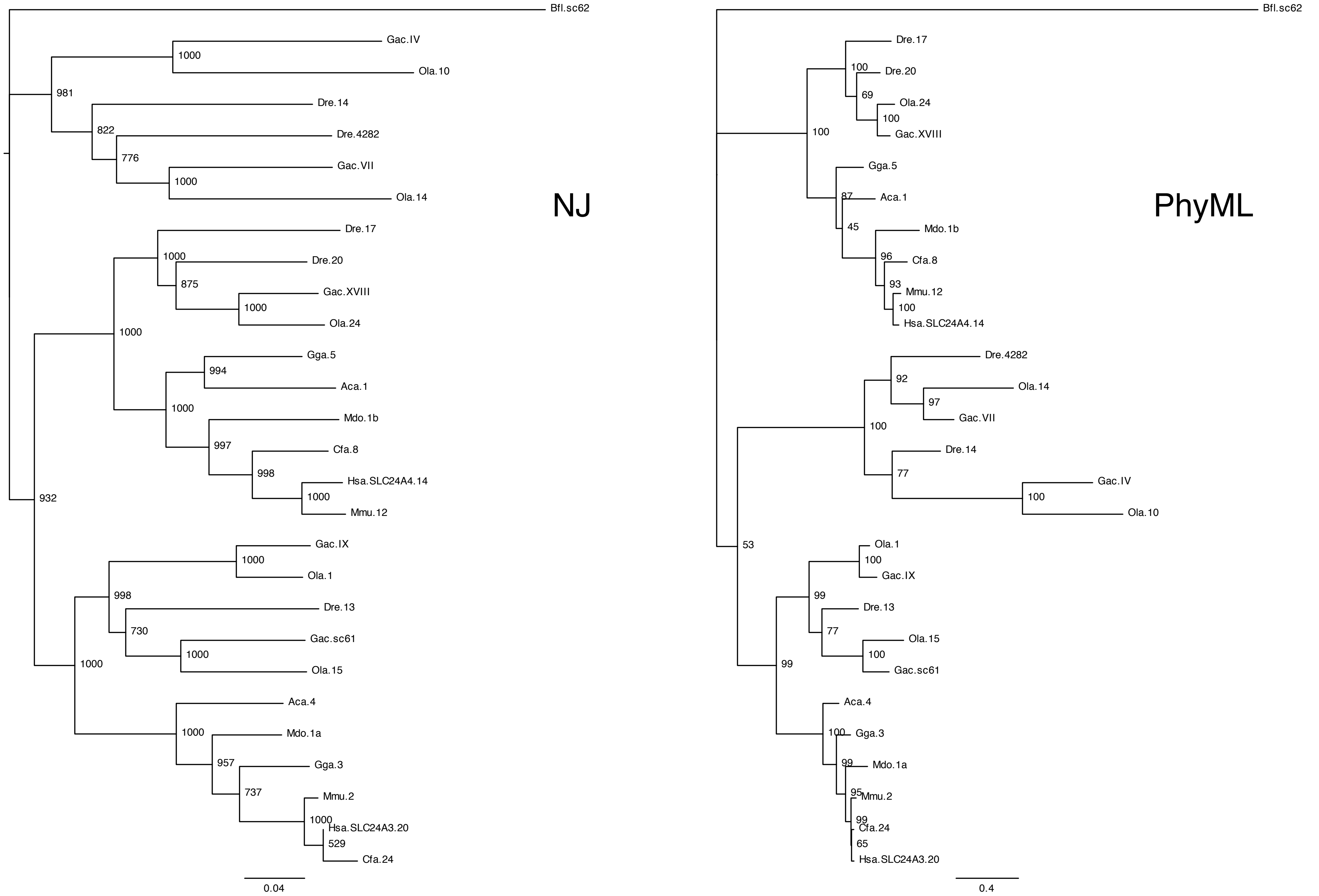

Figure S17. SLC24A

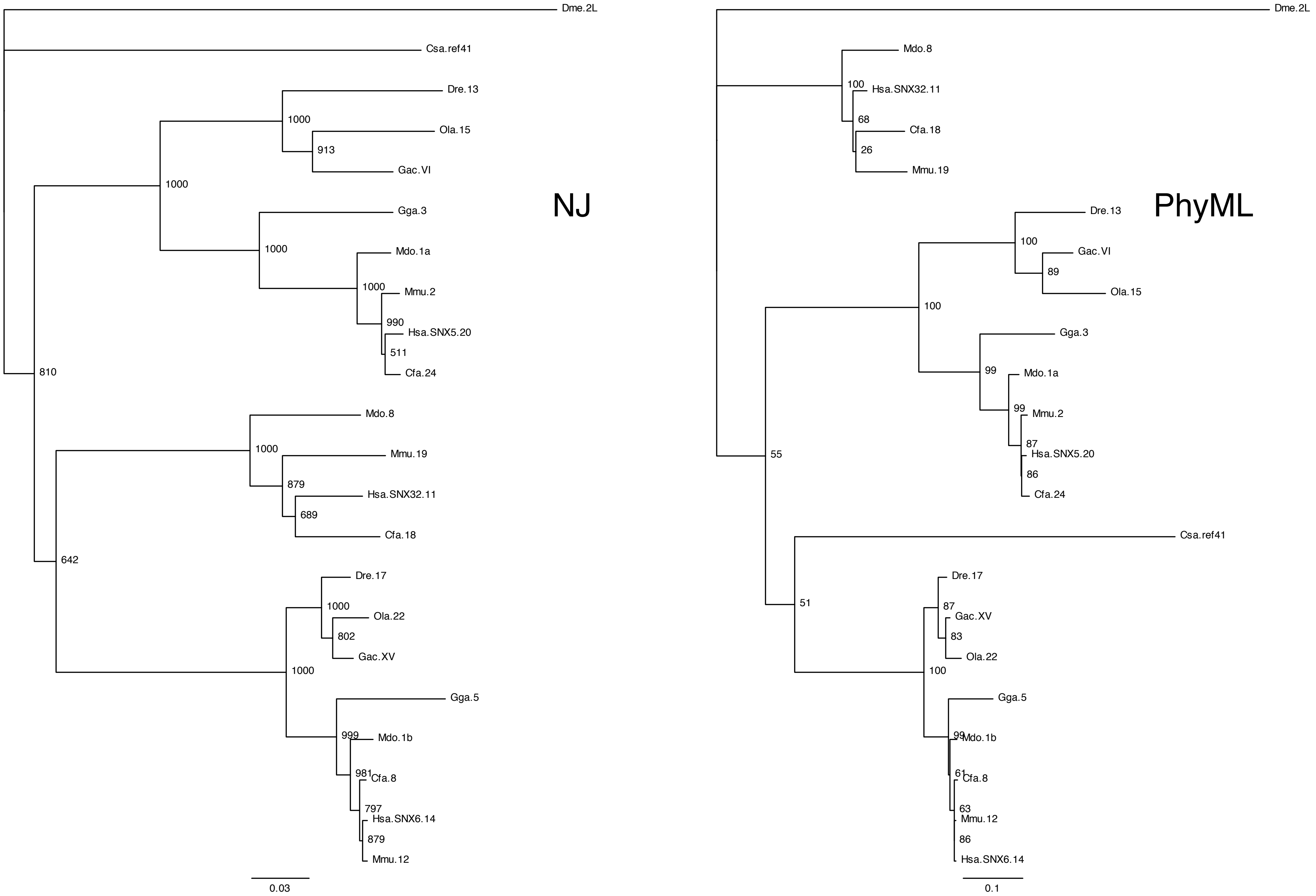

Figure S18. SNX

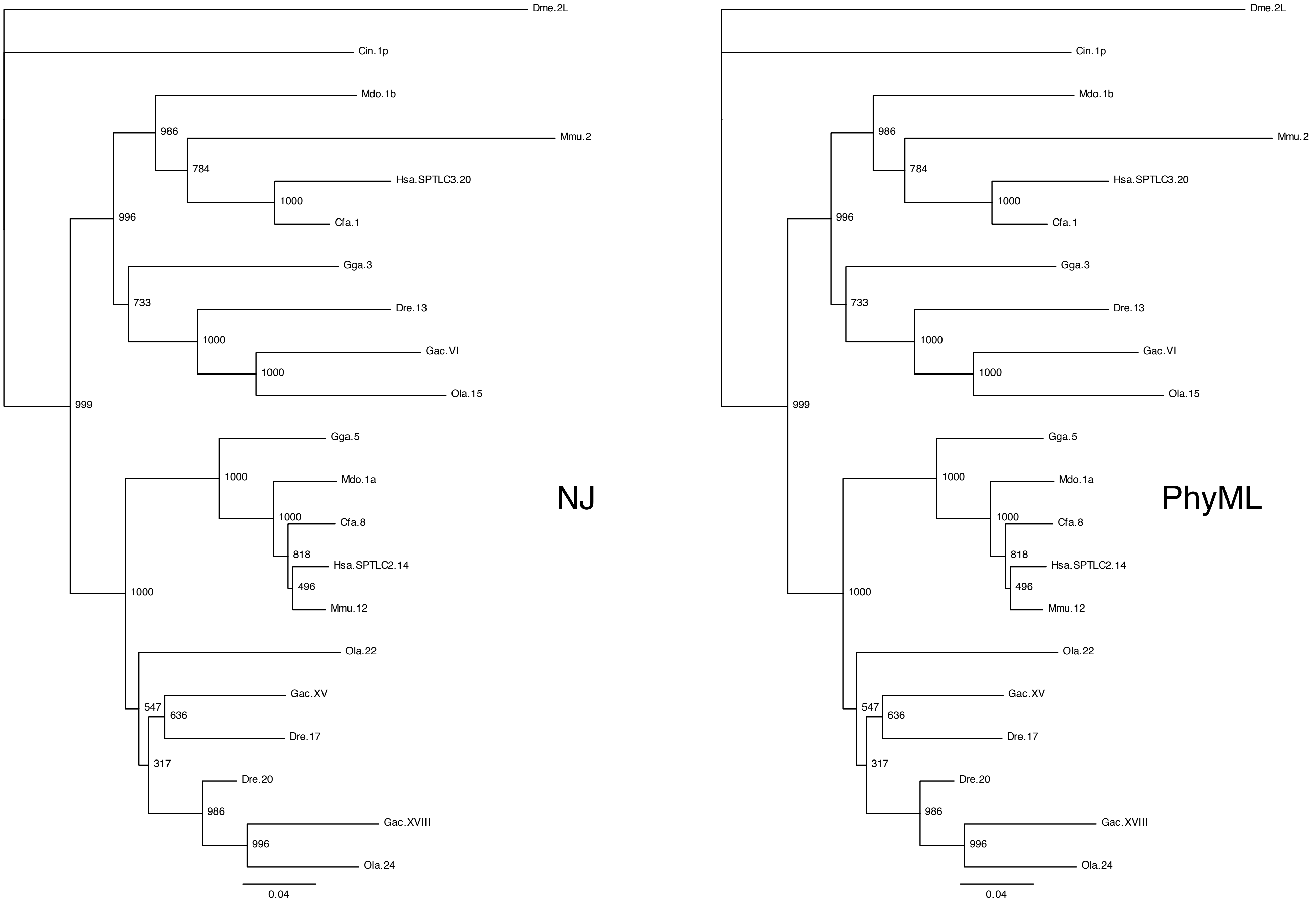

Figure S19. SPTLC

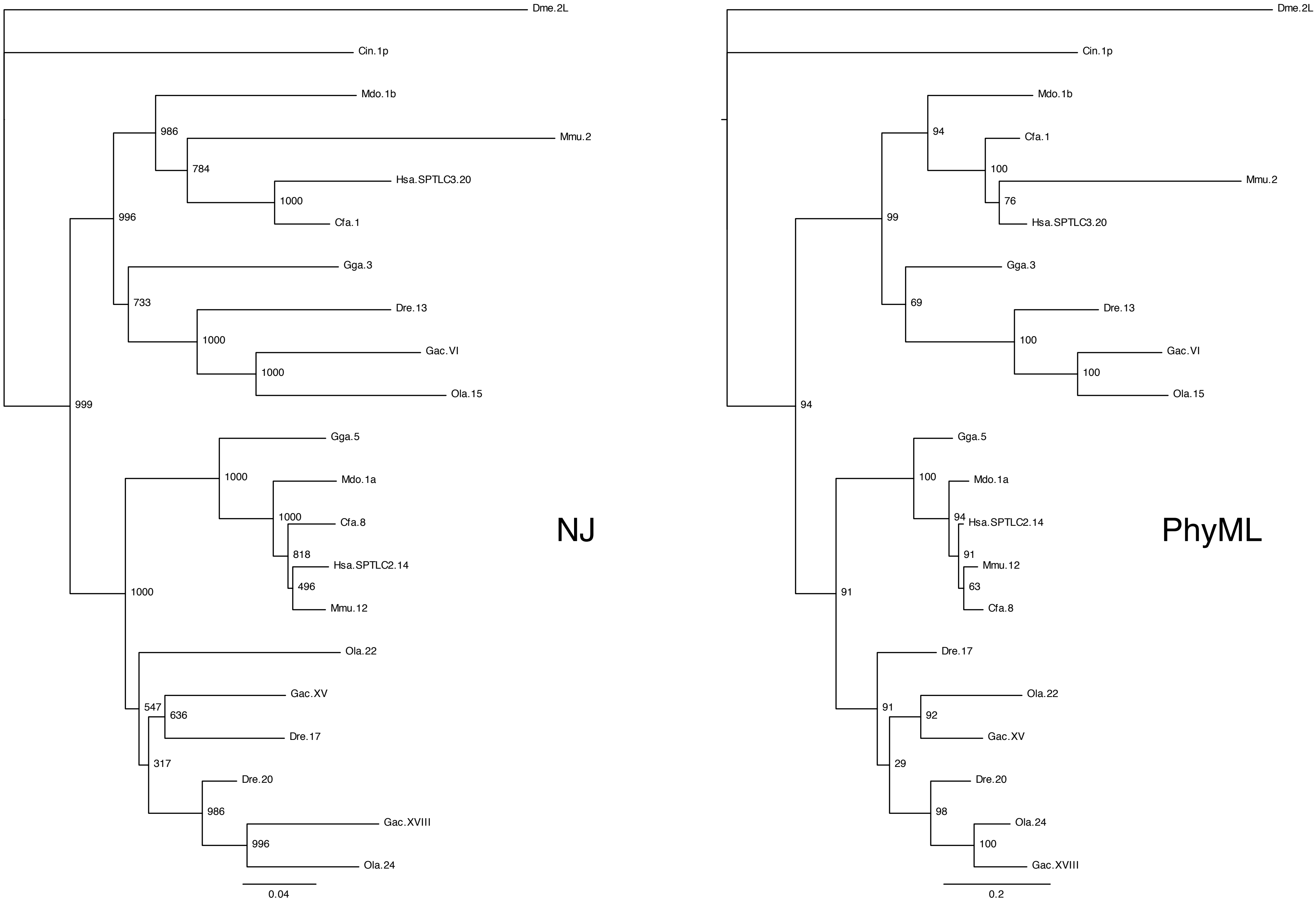

Figure S20. VSX
