# Supplementary material for: The evolution of vertebrate somatostatin receptors and their gene regions involves extensive chromosomal rearrangements
Source: BMC Evol Biol. 2012 Nov 29;12:231. doi: 10.1186/1471-2148-12-231 (PMC3560075; doi:10.1186/1471-2148-12-231)
Supplement: Additional file 6 — Figures S21–S50. Phylogenetic trees of the SSTR2, -3 and -5-neighboring gene families. Figures are numbered S21-S50 and include both neighbor joining and phylogenetic maximum likelihood trees of the gene families described in Table 3. [file 1471-2148-12-231-S6.pdf]

The evolution of vertebrate somatostatin receptors and their gene regions involves extensive chromosomal rearrangements

Daniel **Ocampo Daza***[1], Görel **Sundström**[1, 2], Christina A. **Bergqvist**[1] and Dan **Larhammar**[1]

[1] Department of Neuroscience, Uppsala University, Box 593, SE-75124 Uppsala, Sweden
[2] Present address: Department of Medical Biochemistry and Microbiology, Uppsala University, Box 582, SE-75123 Uppsala, Sweden
* Corresponding author
Telephone: +46-18-4714173
Fax: +46-18-511540

SUPPLEMENTAL FIGURES 21-50

Figures S21-50: Bootstrapped Neighbor Joining (NJ) and Phylogenetic Maximum Likelihood (PhyML) trees of the *SSTR2, -3* and *-5*-neighboring gene families. The topologies are supported by non-parametric bootstrap analyses with 1000 (NJ) or 100 (PhyML) replicates (see Methods). Branch support is shown at the nodes. The trees are rooted with the identified fruit fly sequences, unless specified in Table 3. Species abbreviations are applied as follows: *Homo sapiens* (Hsa), *Mus musculus* (Mmu), *Canis familiaris* (Cfa), *Monodelphis domestica* (Mdo), *Macropus eugenii* (Meu), *Gallus gallus* (Gga), *Meleagris gallopavo* (Mga), *Taeniopygia guttata* (Tgu), *Anolis carolinensis* (Aca), *Silurana (Xenopus) tropicalis* (Xtr), *Danio rerio* (Dre), *Oryzias latipes* (Ola), *Gasterosteus aculeatus* (Gac), *Tetraodon nigroviridis* (Tni), *Takifugu rubripes* (Tru), *Ciona intestinalis* (Cin), *Ciona savignyi* (Csa), *Branchiostoma floridae* (Bfl), *Caenorhabditis elegans* (Cel) and *Drosophila melanogaster* (Dme). See Methods – "*Identification and analysis of neighboring gene families/Conserved synteny analysis"* - for the choice of species representation. HGNC symbols are used for all human sequences. Other species' sequences are identified by chromosomal/genomic scaffold assignment. Lowercase a, b and c are used to distinguish sequences located on the same chromosomes. Comprehensive information about each sequence is available in Additional file 9, Table S3.

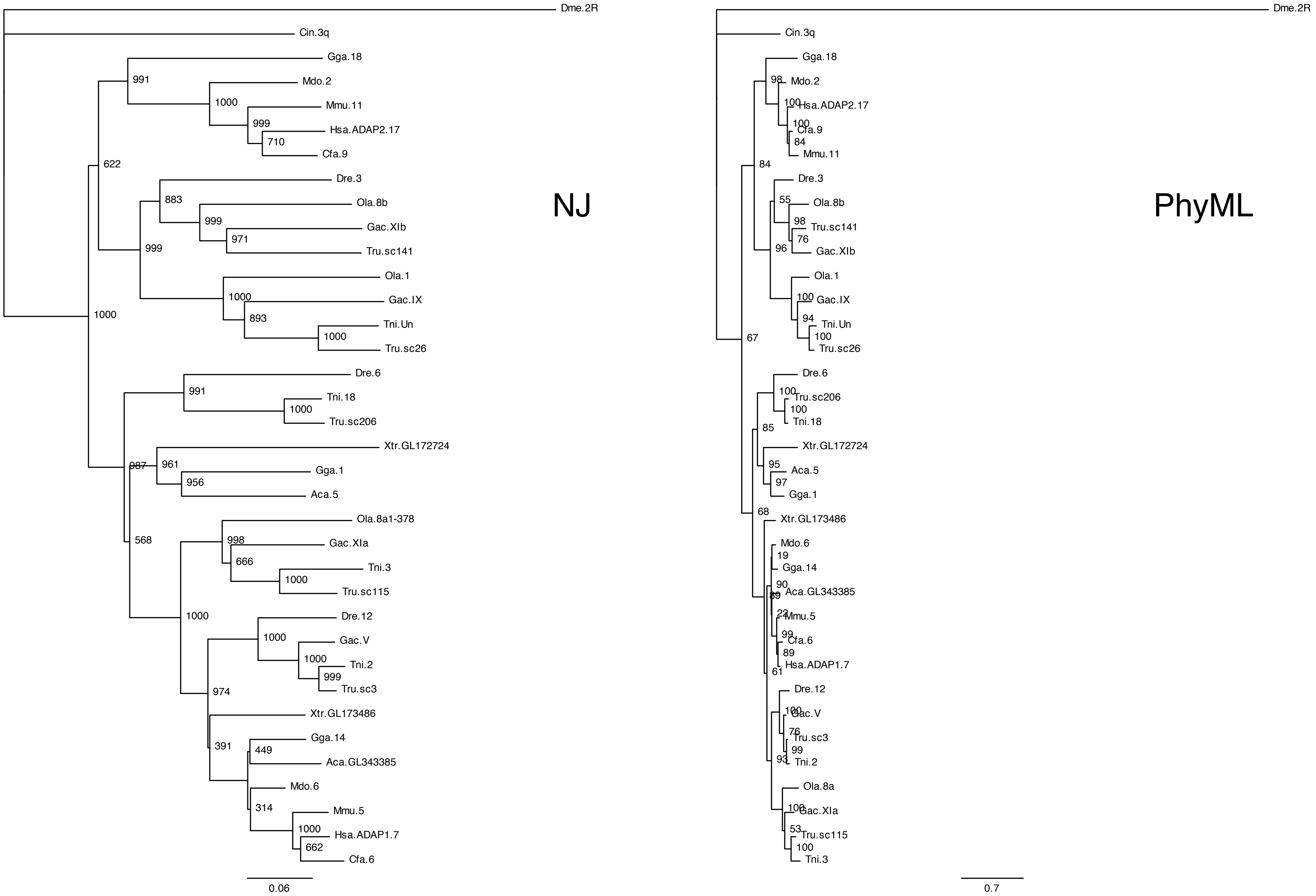

Figure S21. ADAP

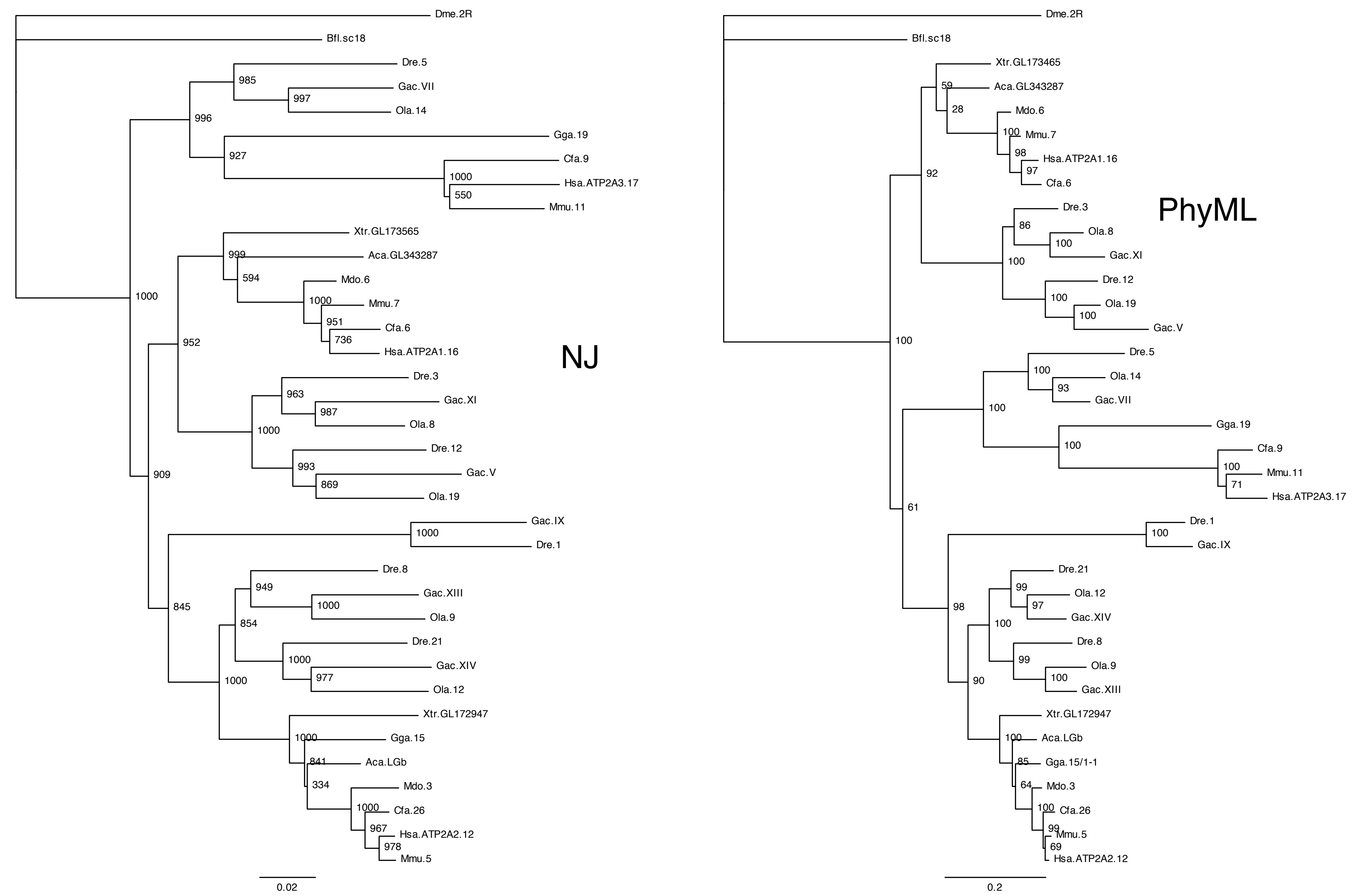

Figure S22. ATP2A

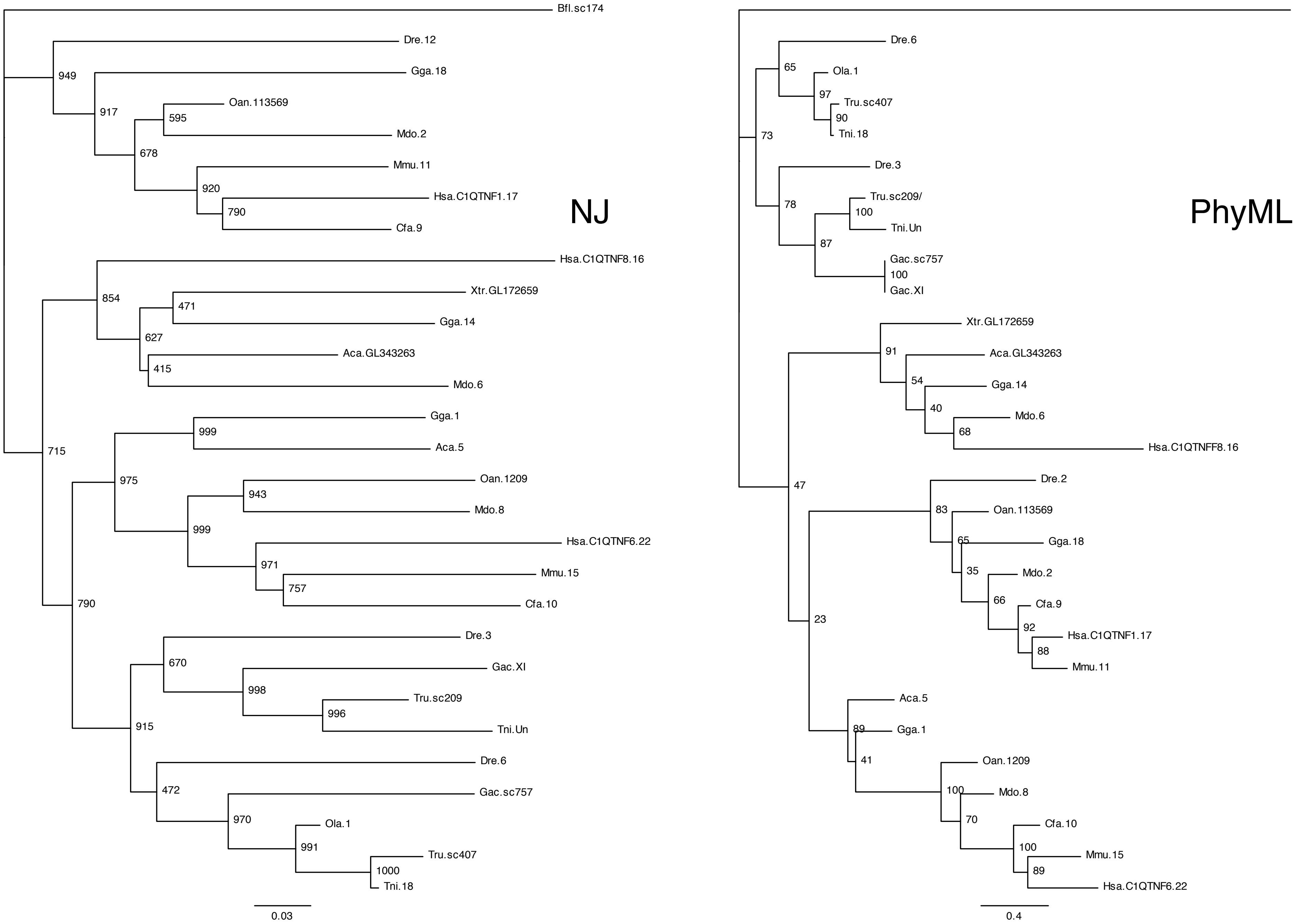

Figure S23. C1QTNF

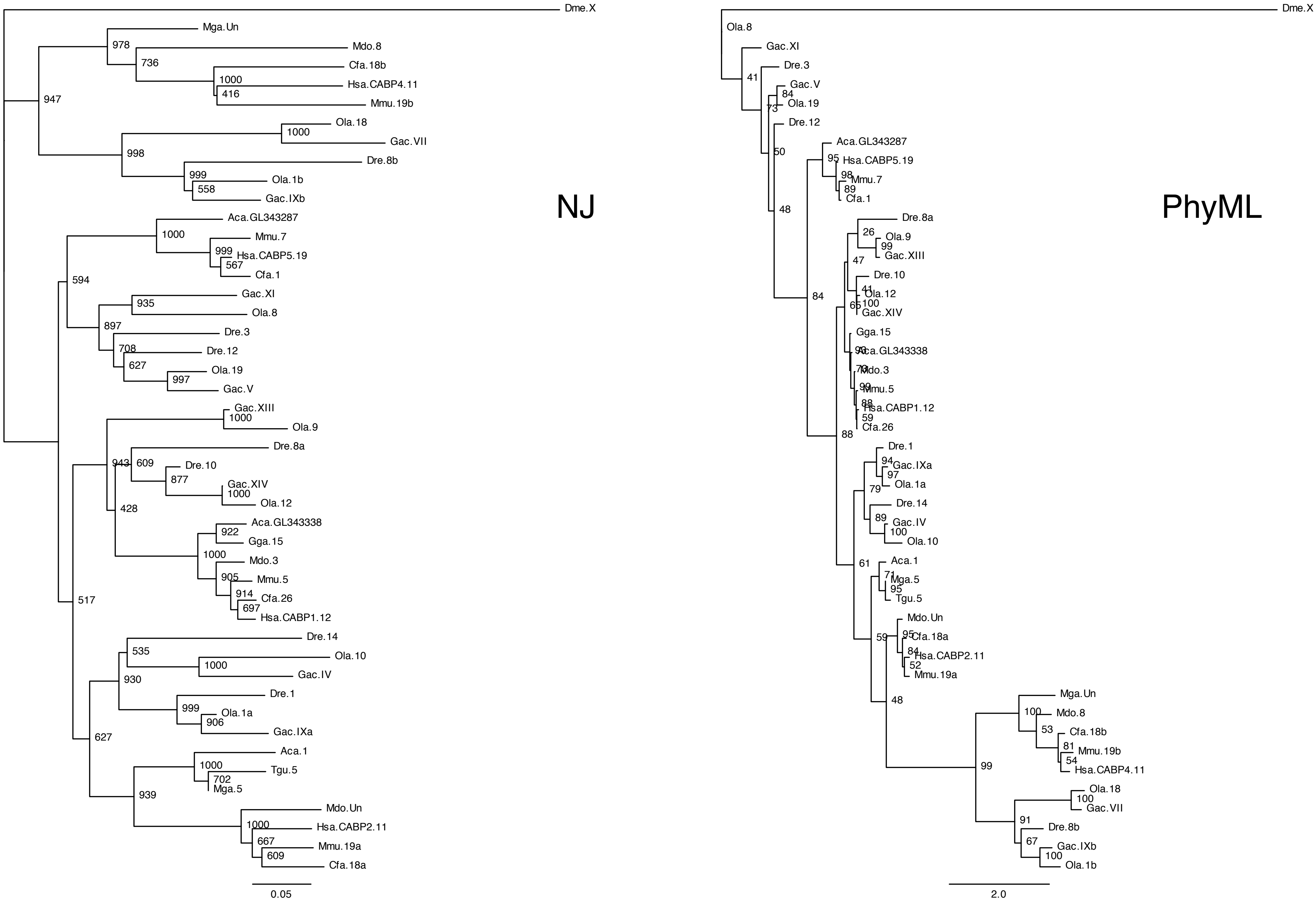

Figure S24. CABP

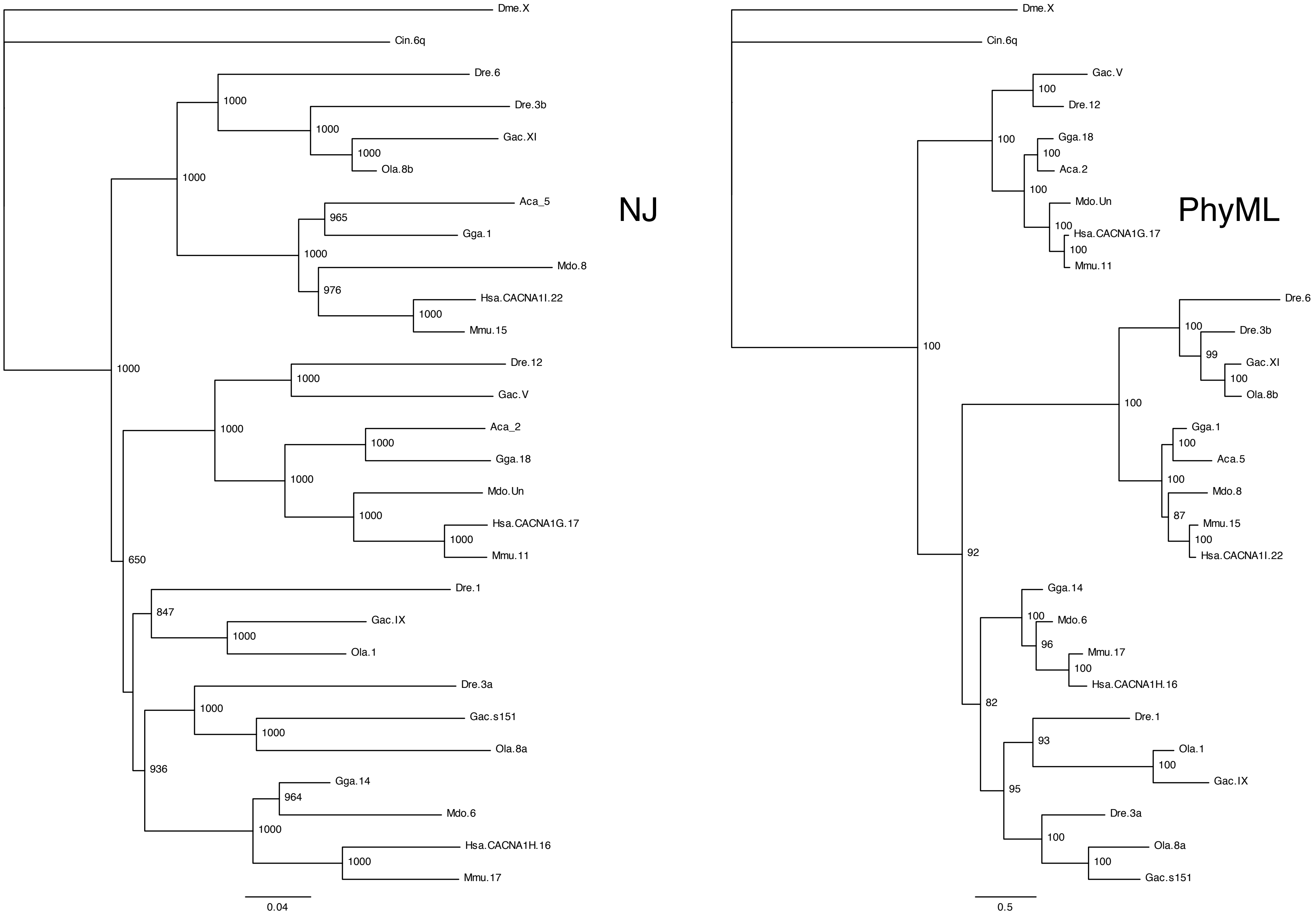

Figure S25. CACNA1

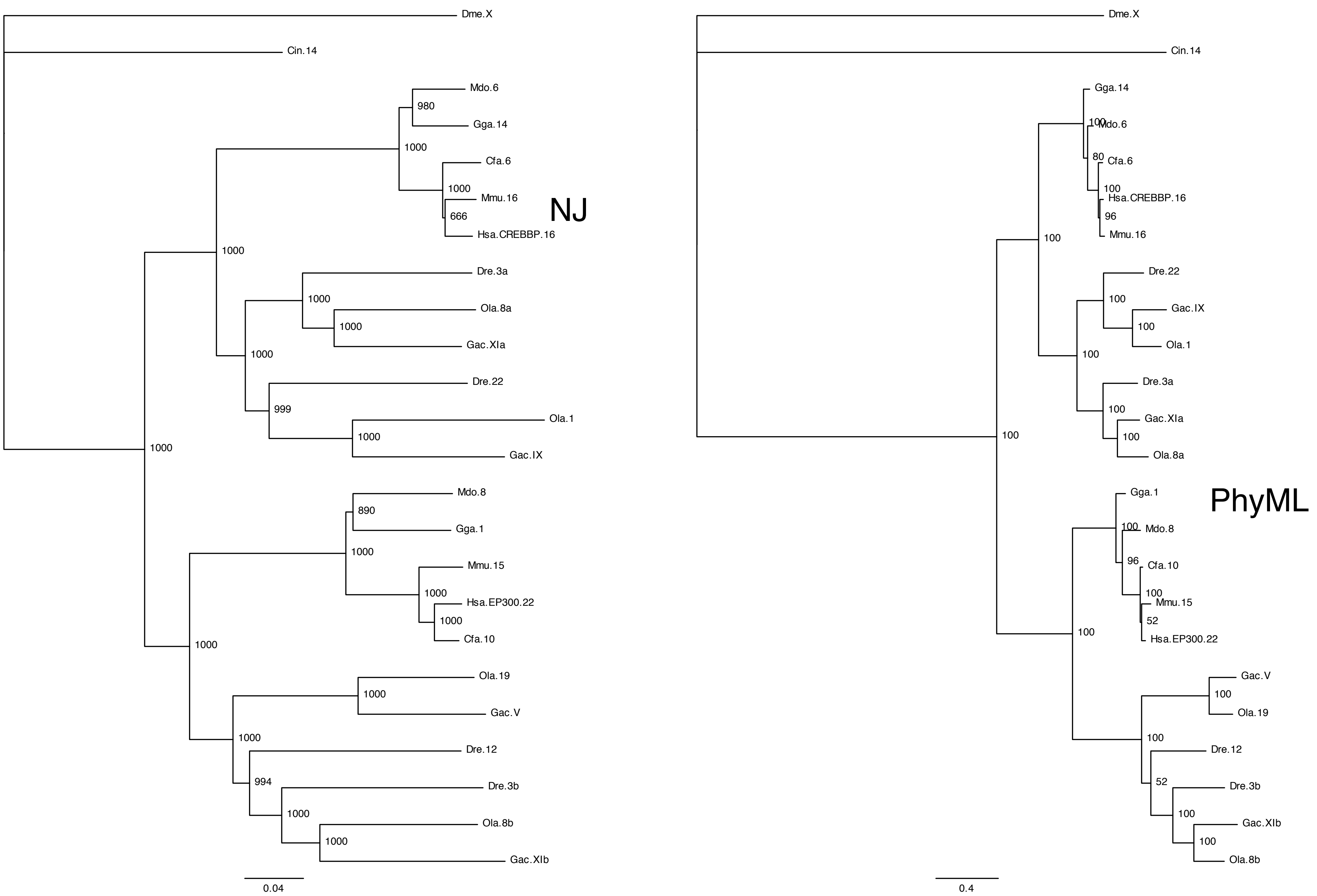

Figure S26. CREBBP

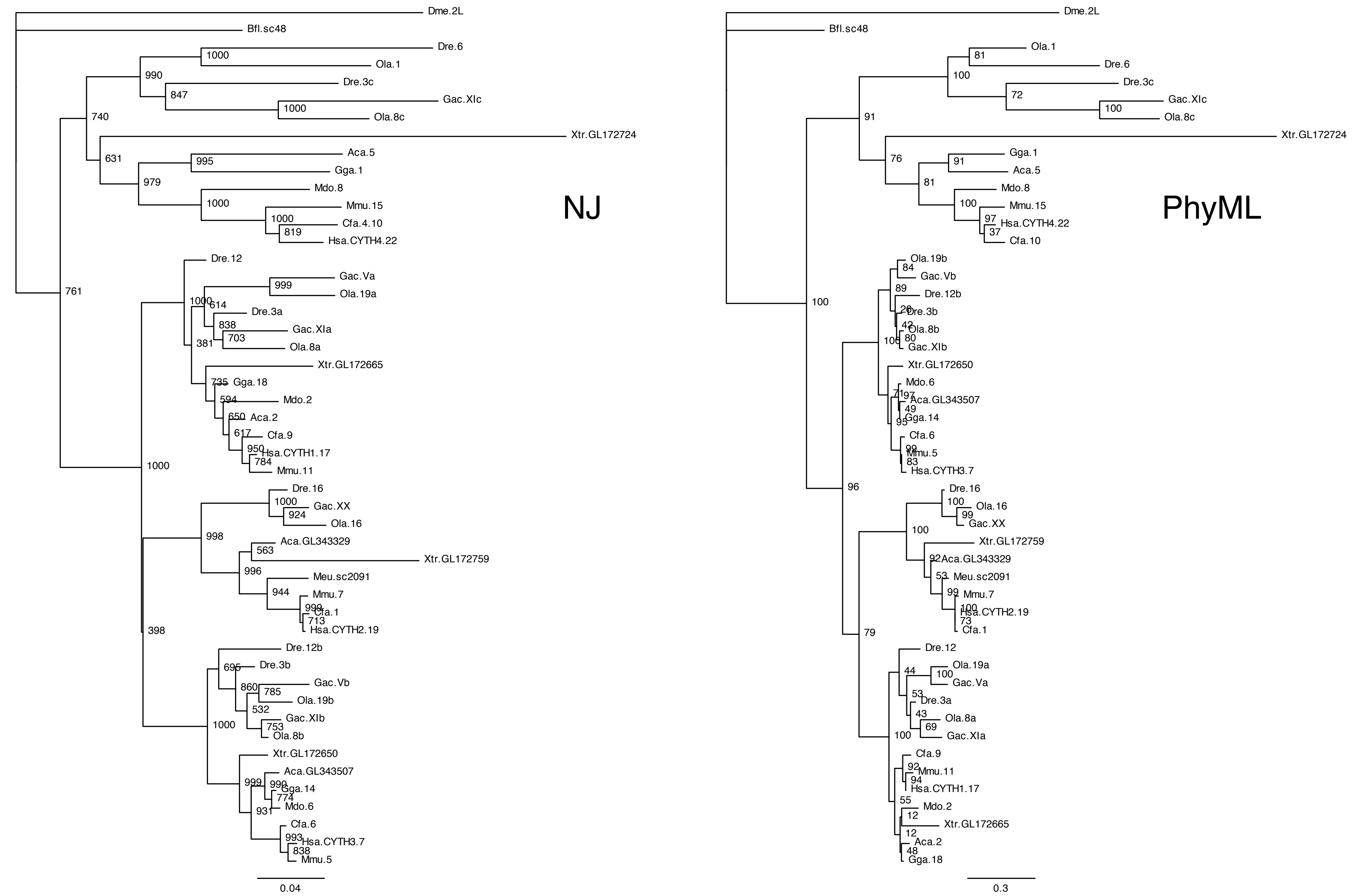

Figure S27. CYTH

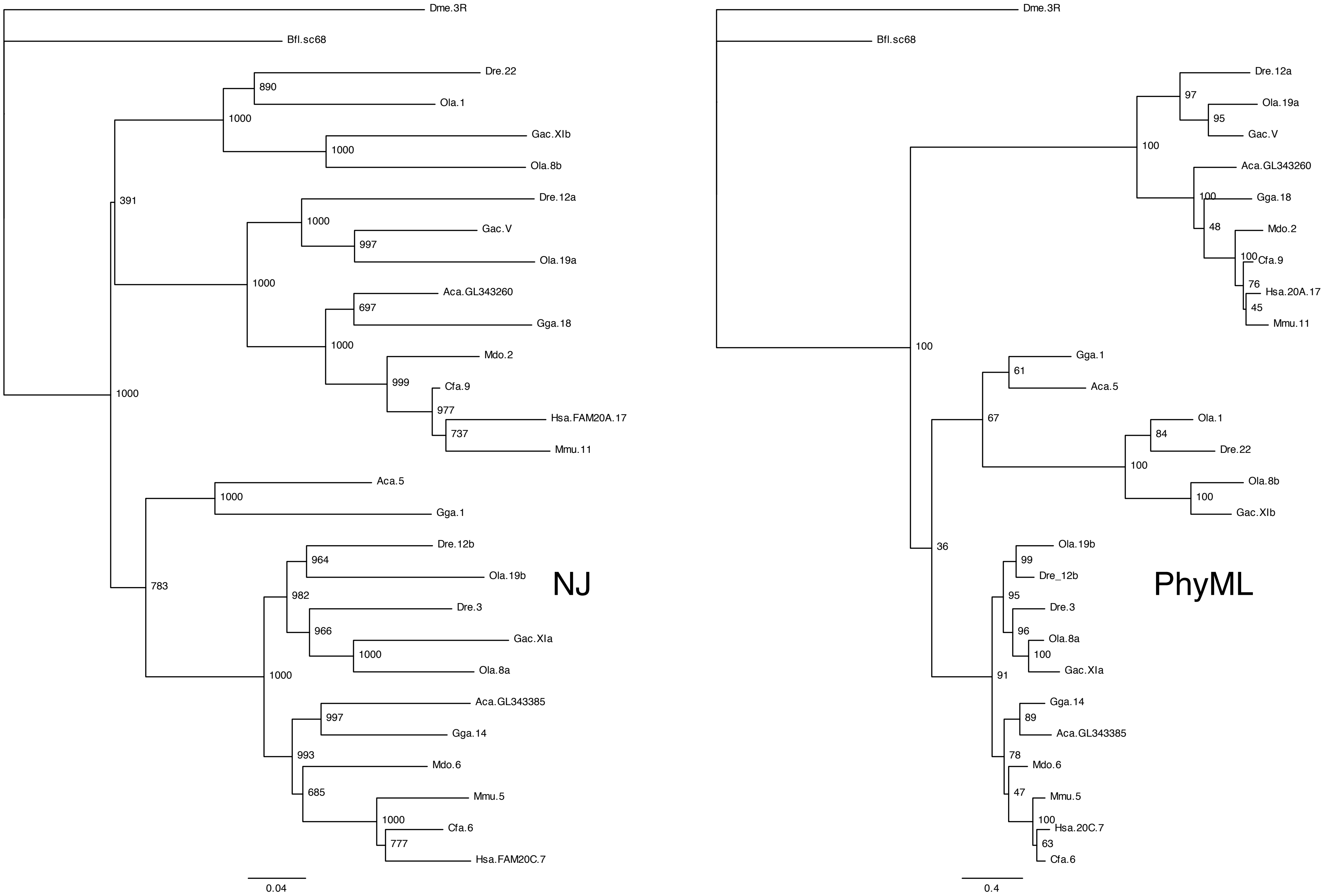

Figure S28. FAM20

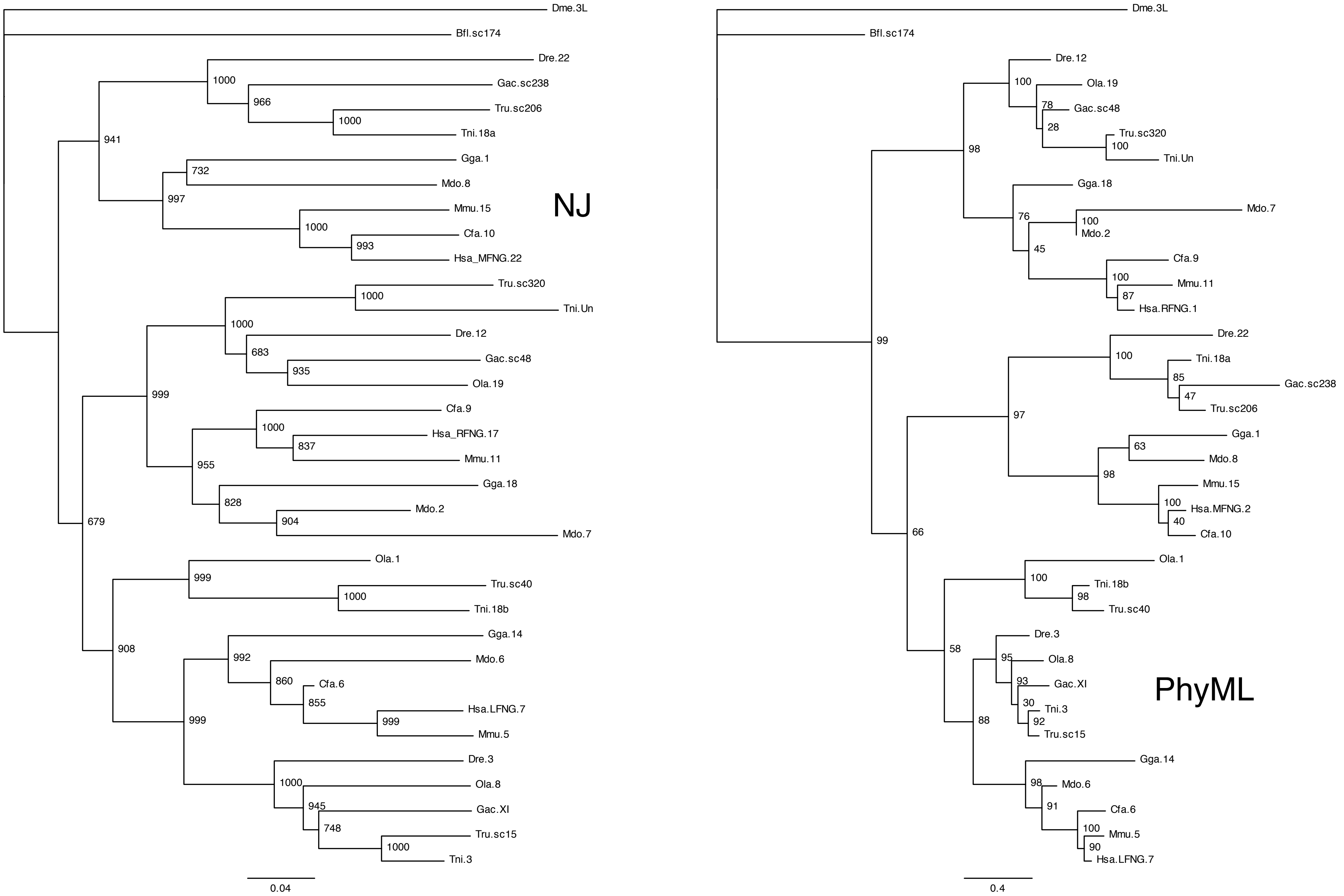

Figure S29. FNG

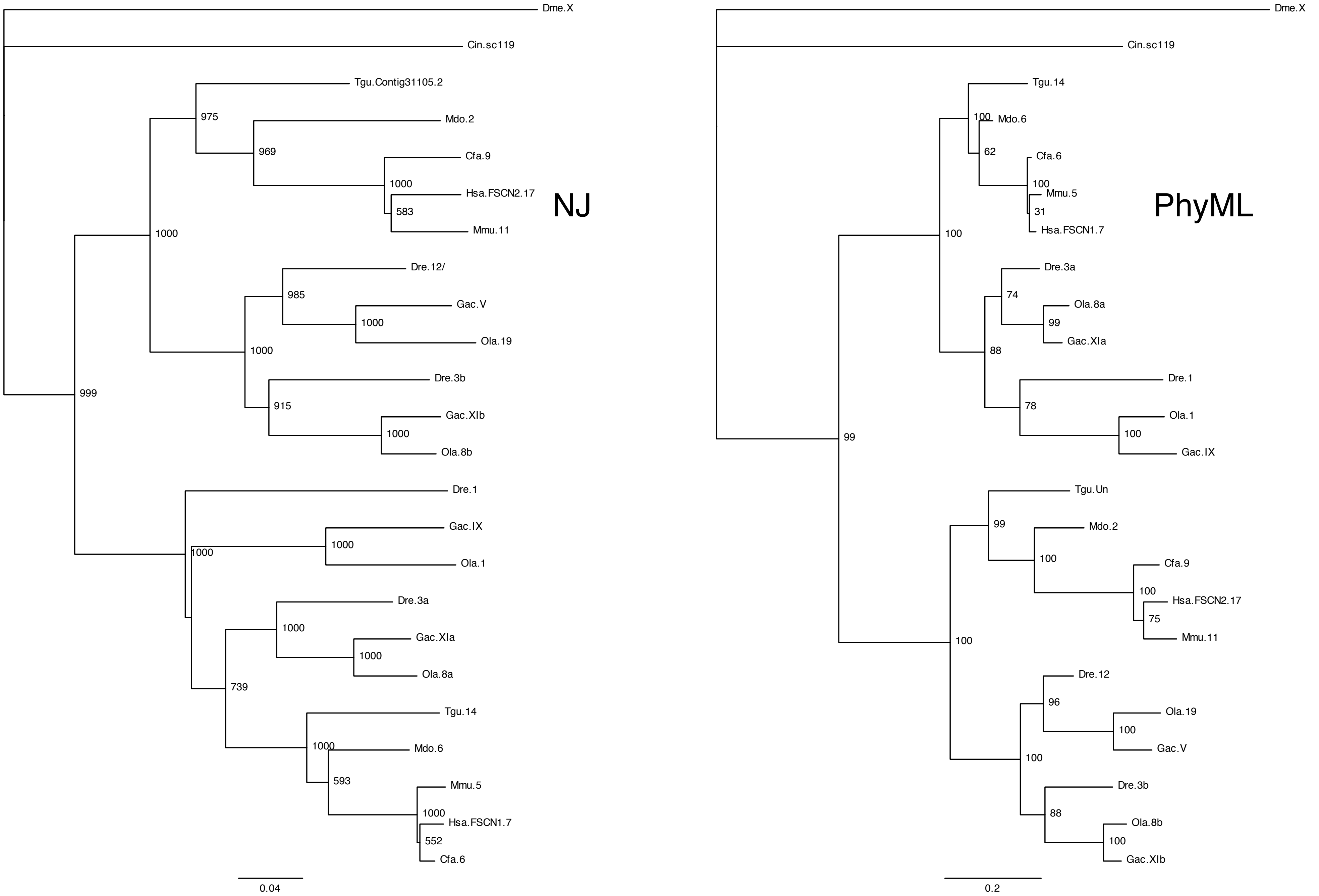

Figure S30. FSCN

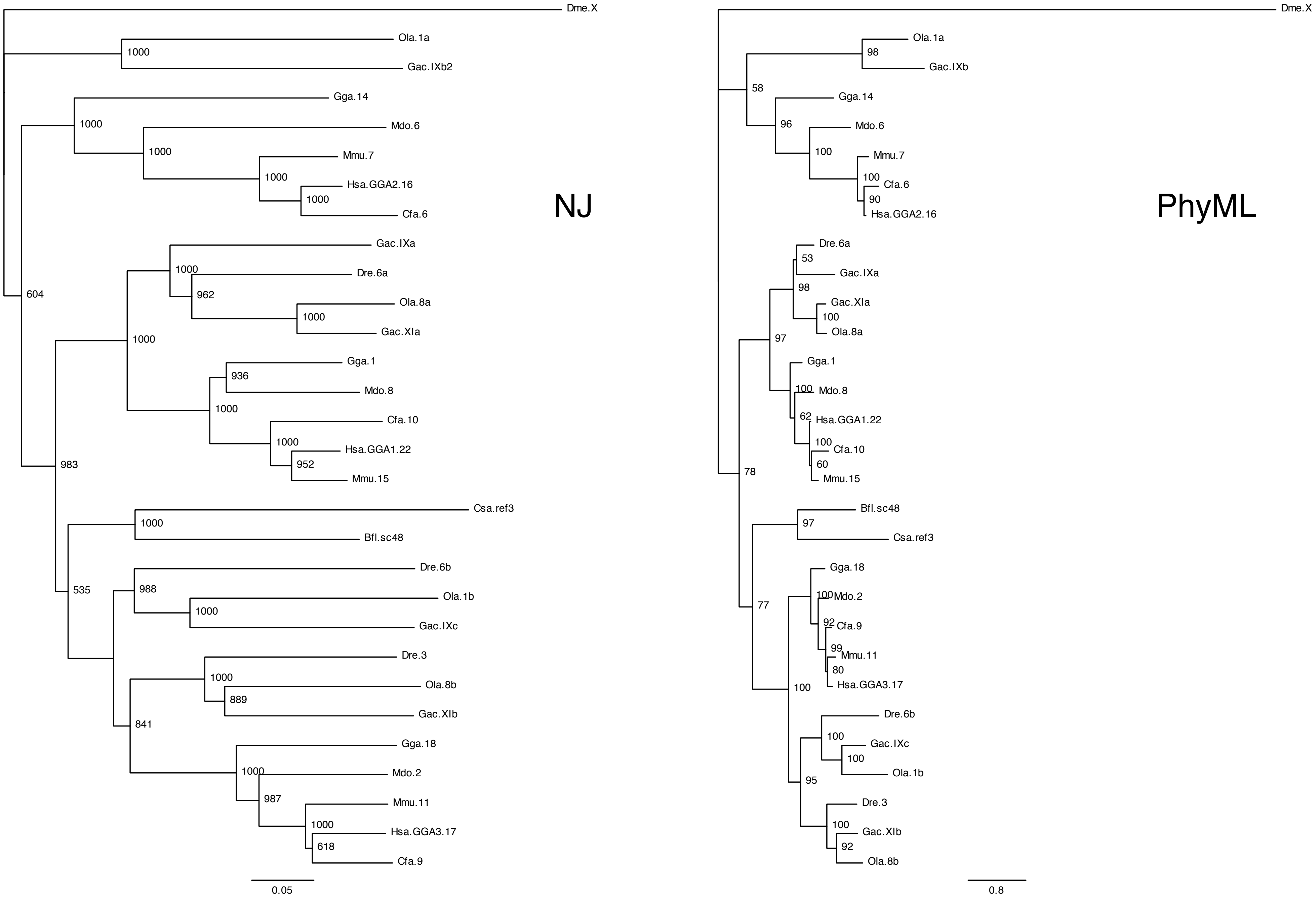

Figure S31. GGA

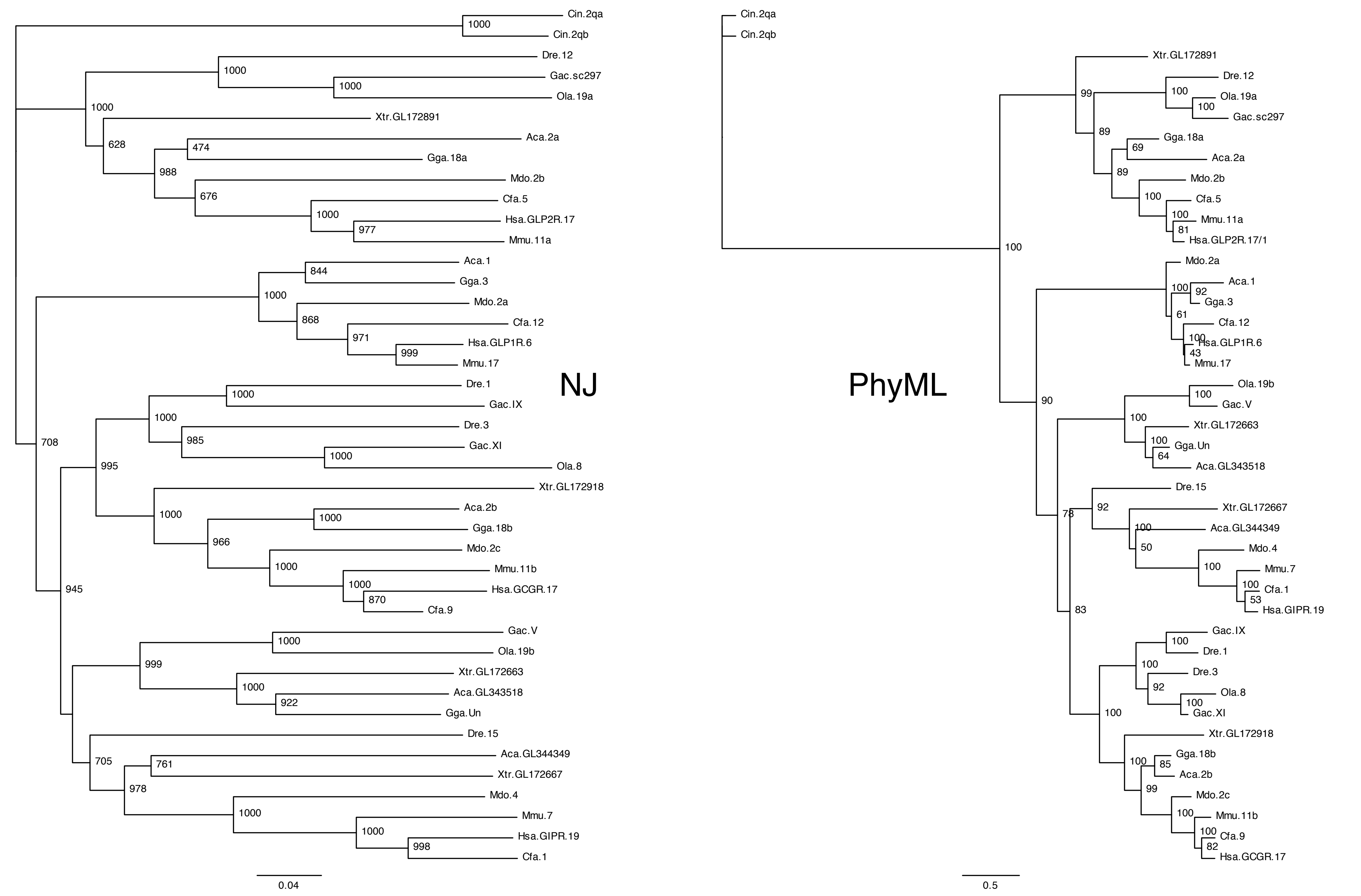

Figure S32. GLPR

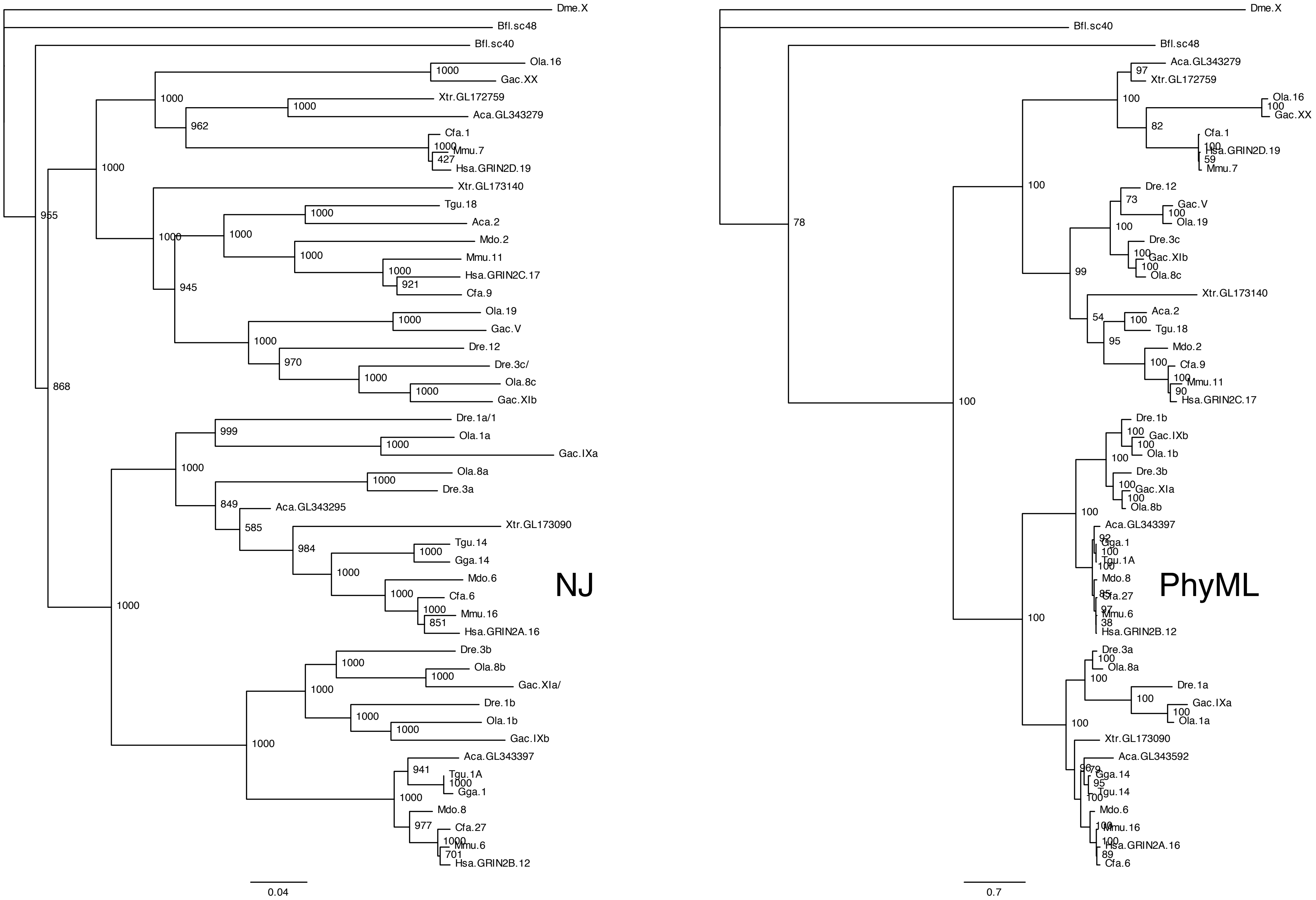

Figure S33. GRIN2

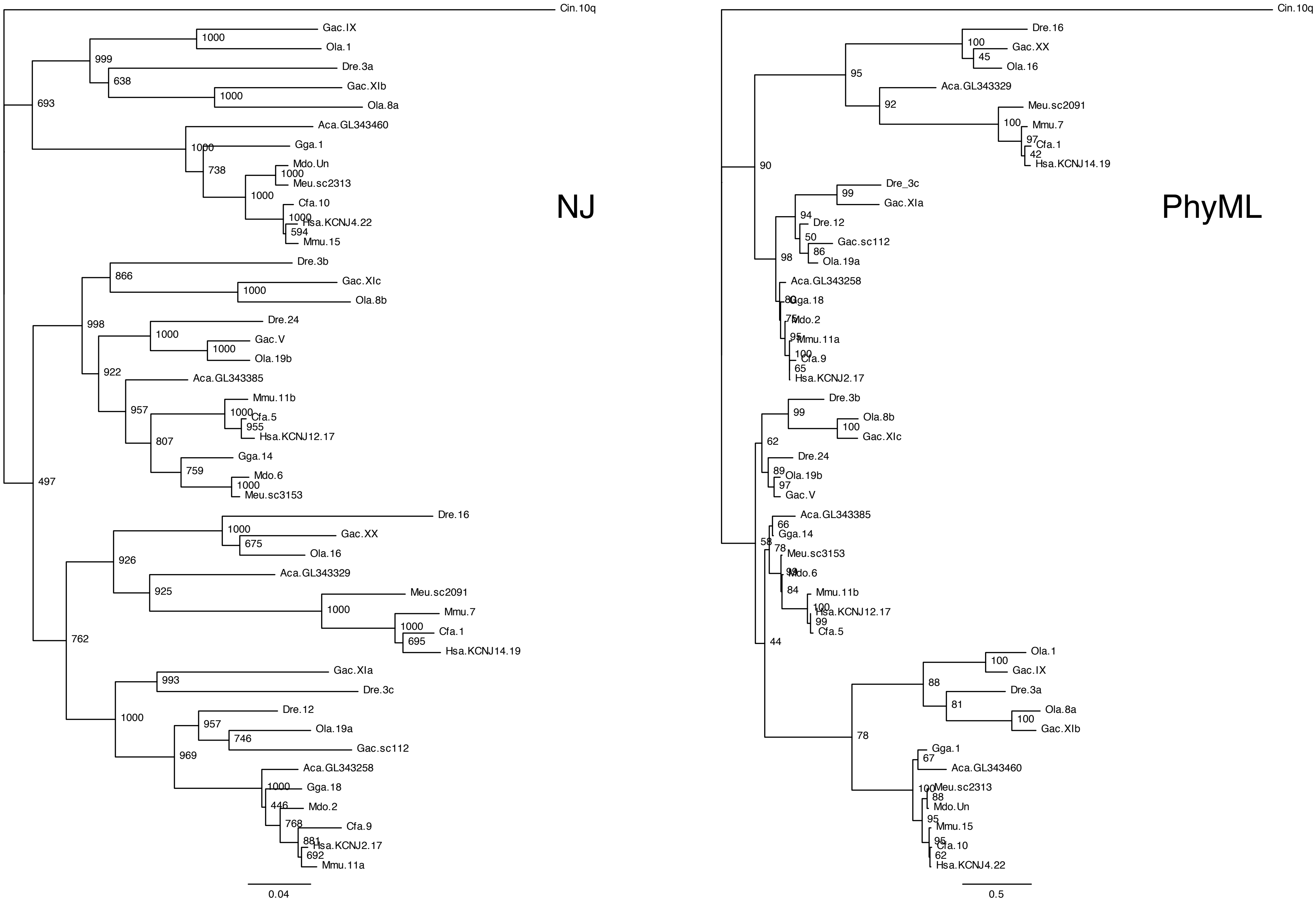

Figure S34. KCNJ

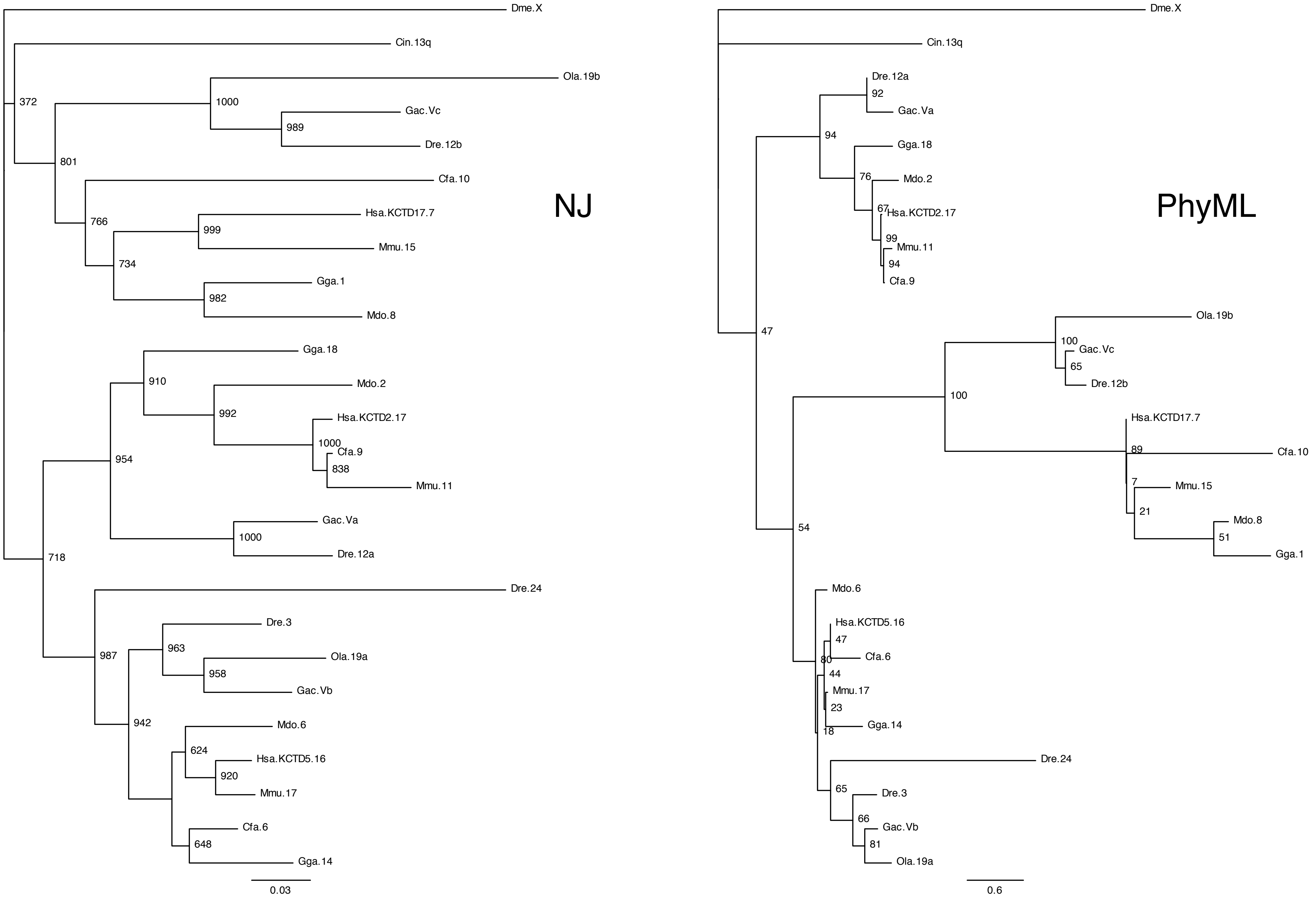

Figure S35. KCTD

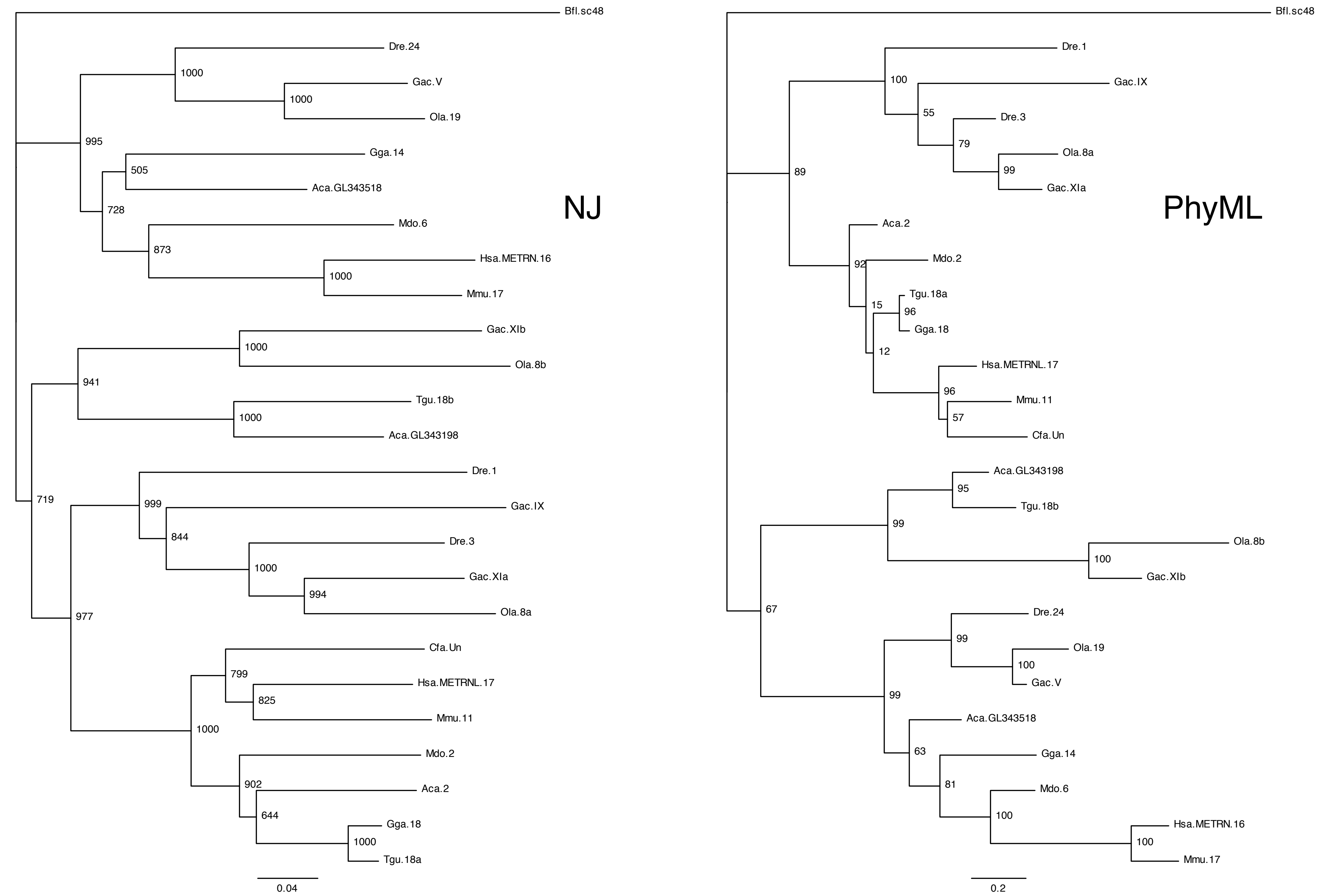

Figure S36. METRN

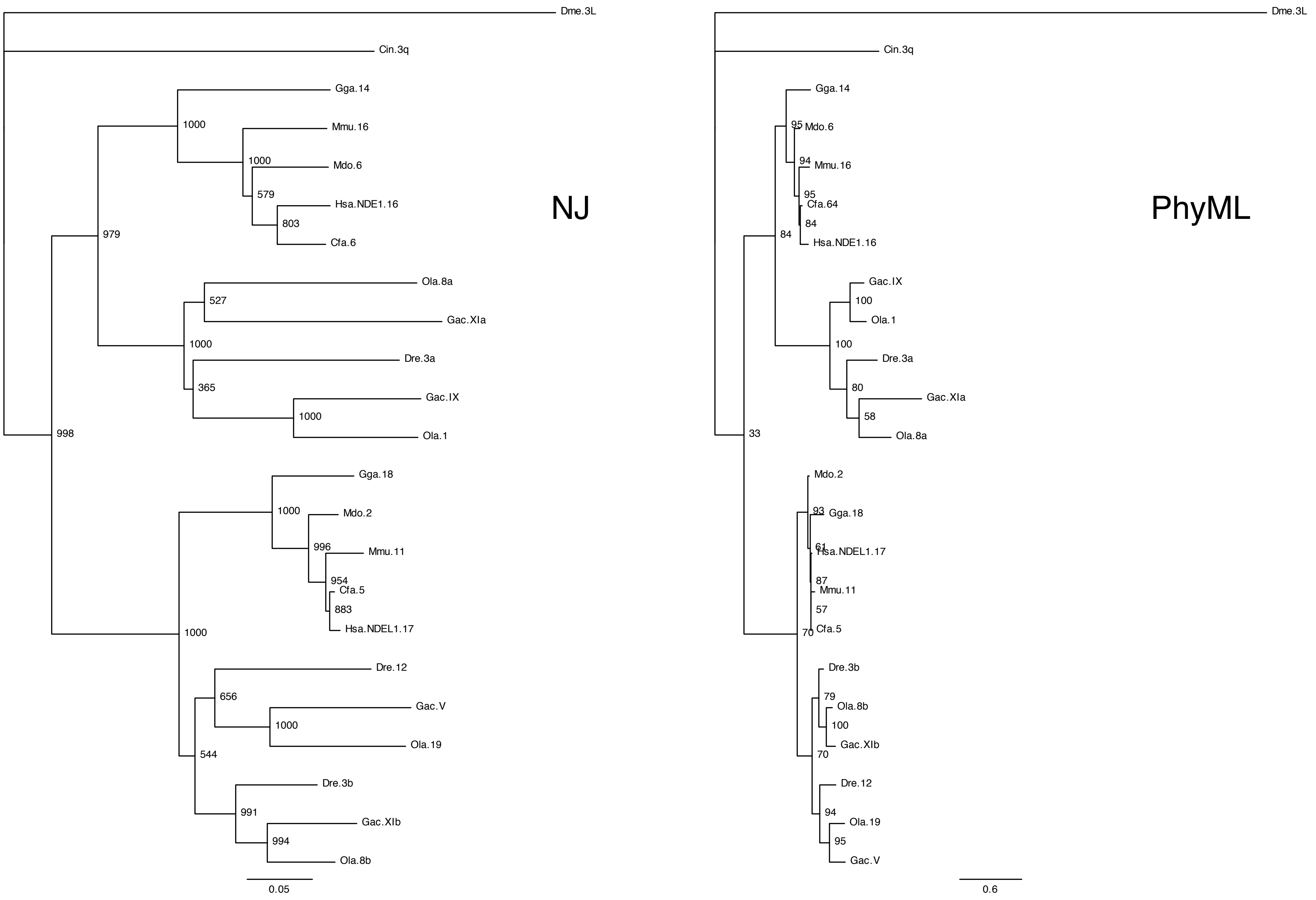

Figure S37. NDE

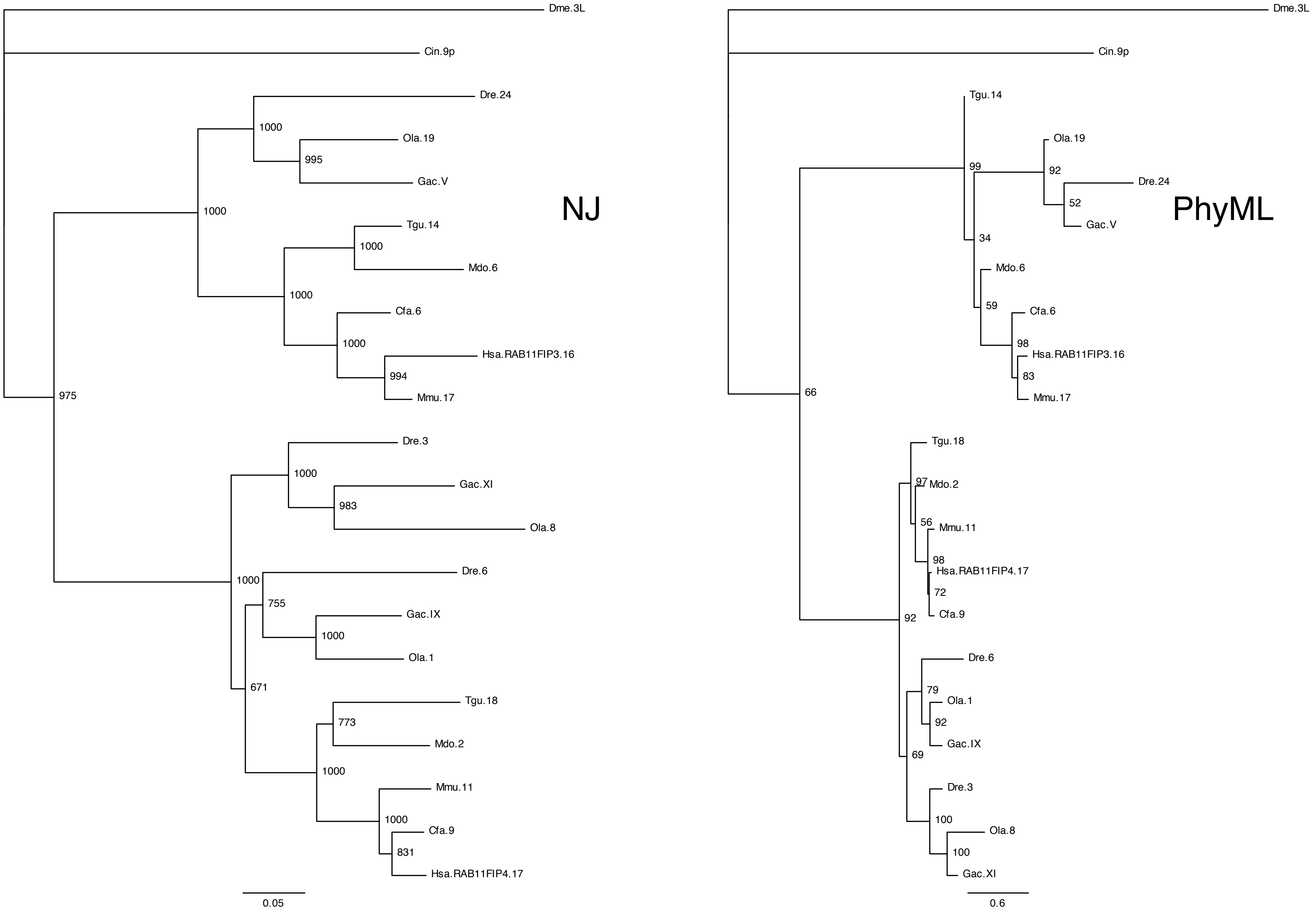

Figure S38. RAB11FIP

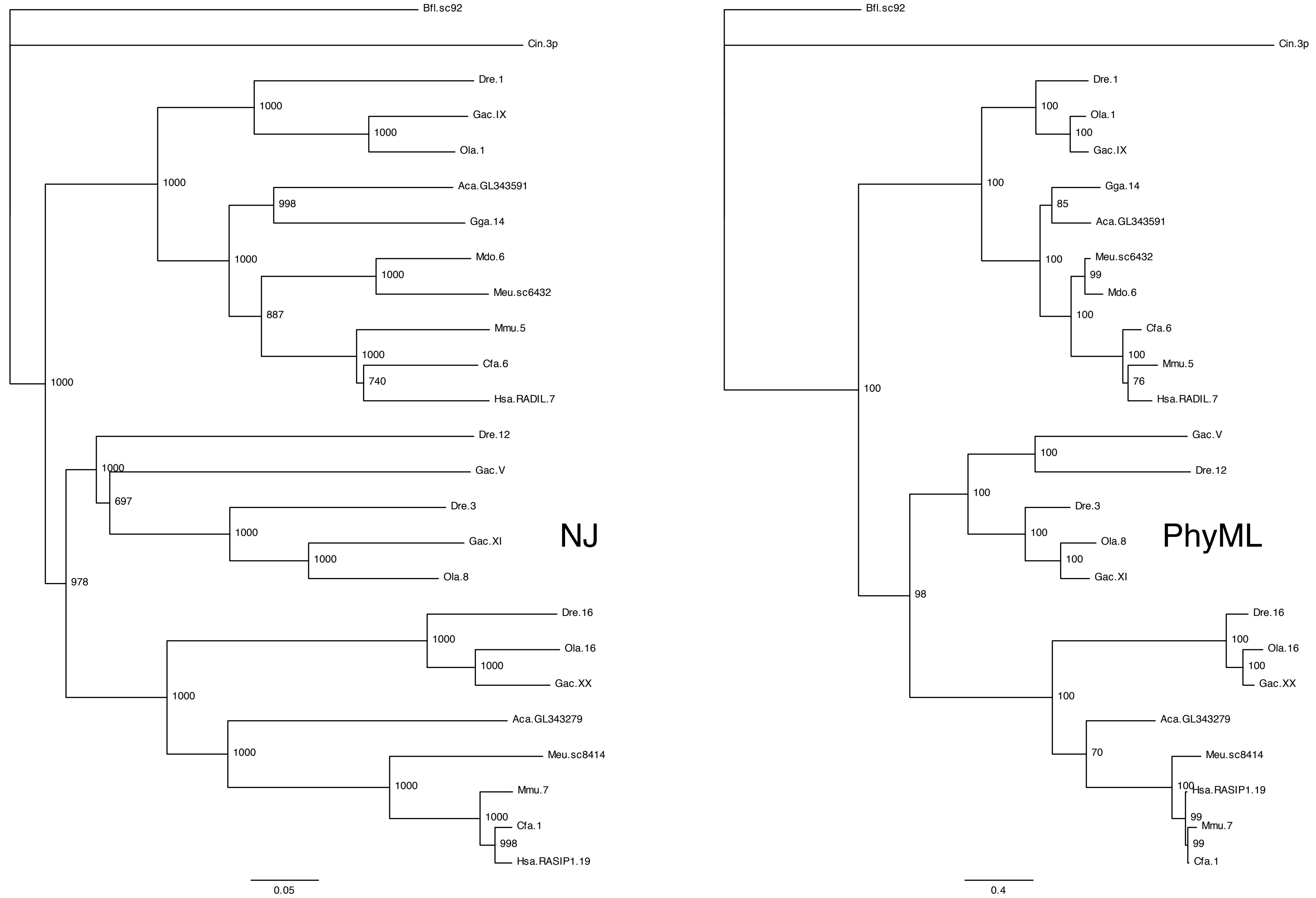

Figure S39. RADIL

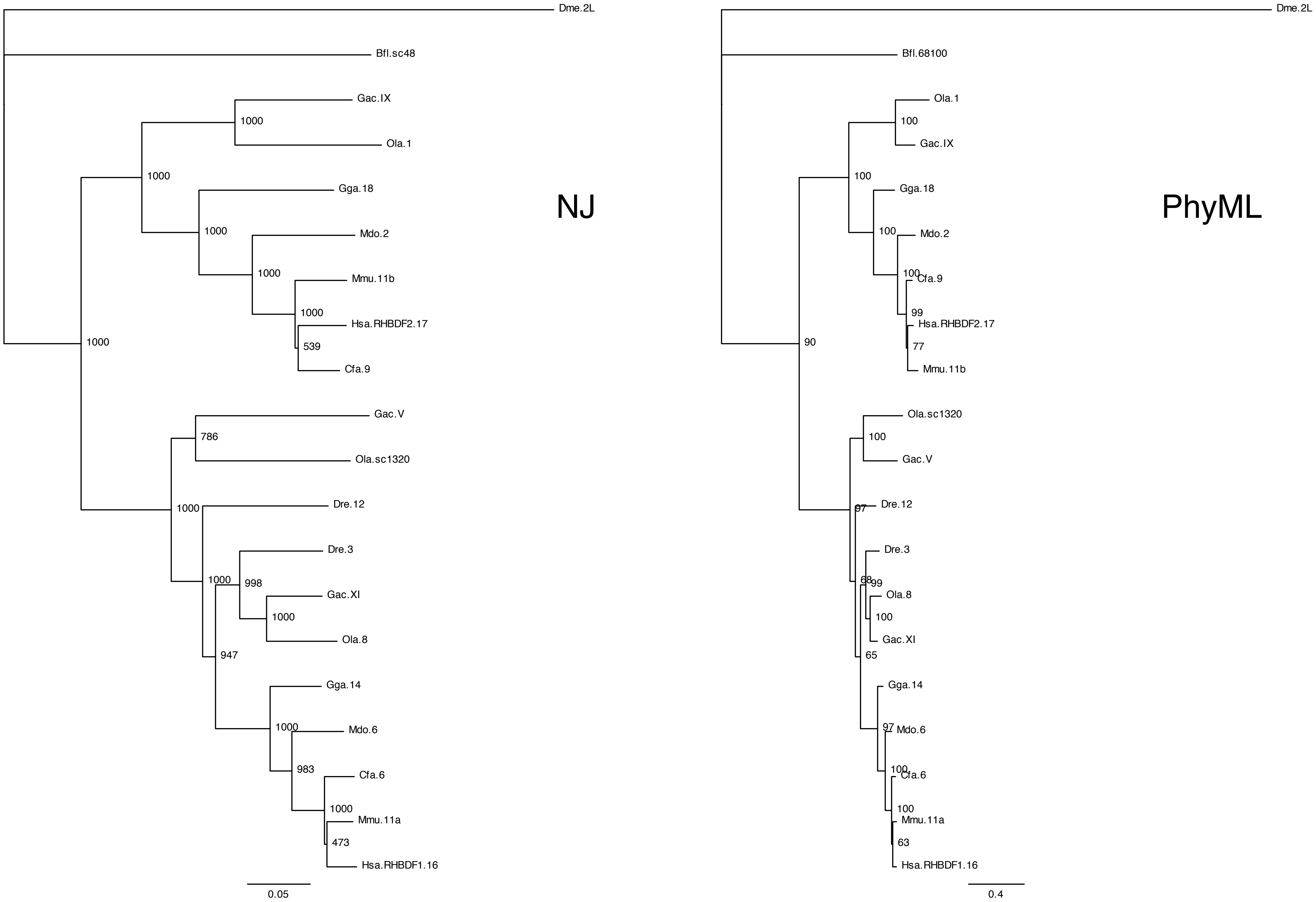

Figure S40. RHBDF

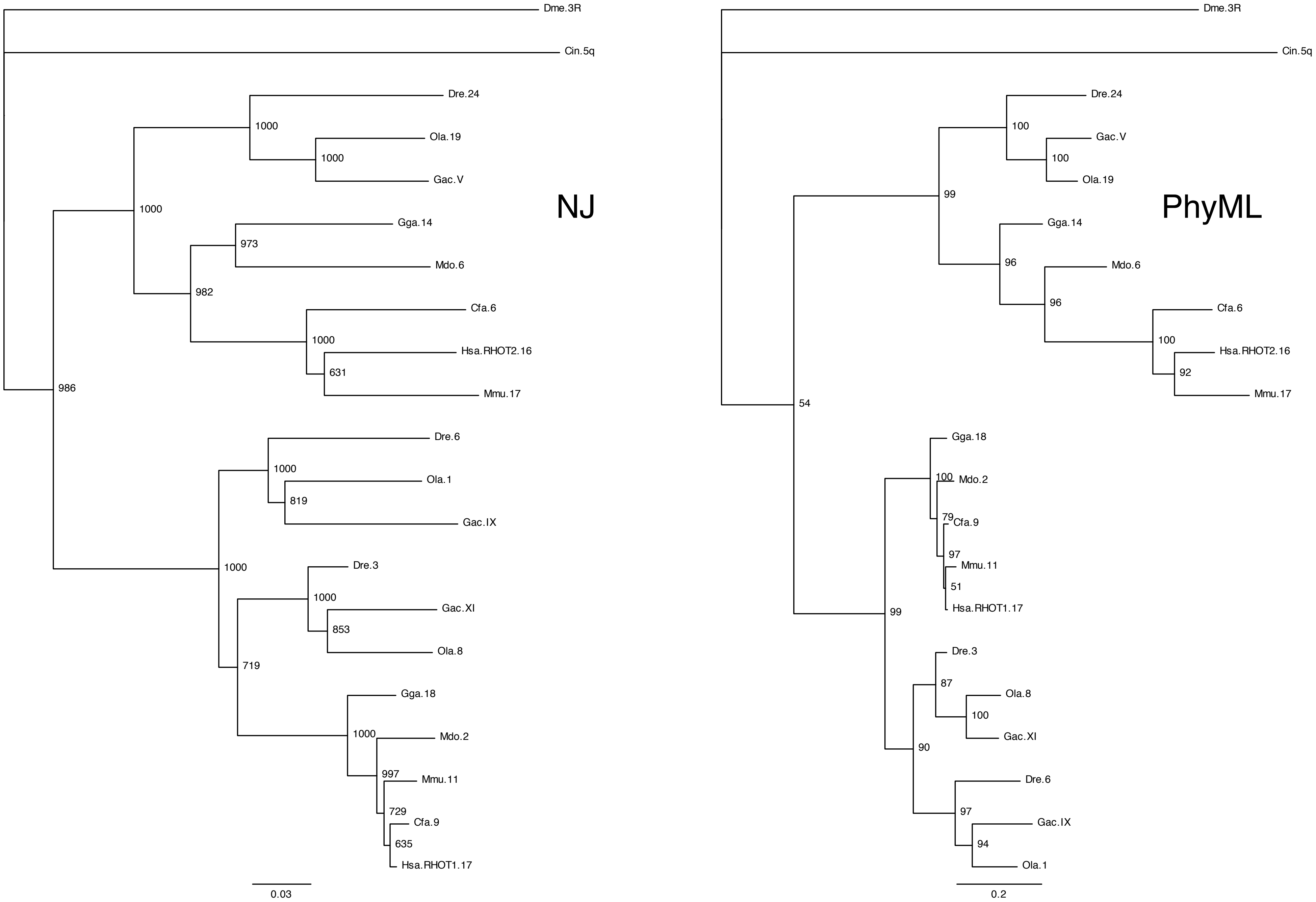

Figure S41. RHOT

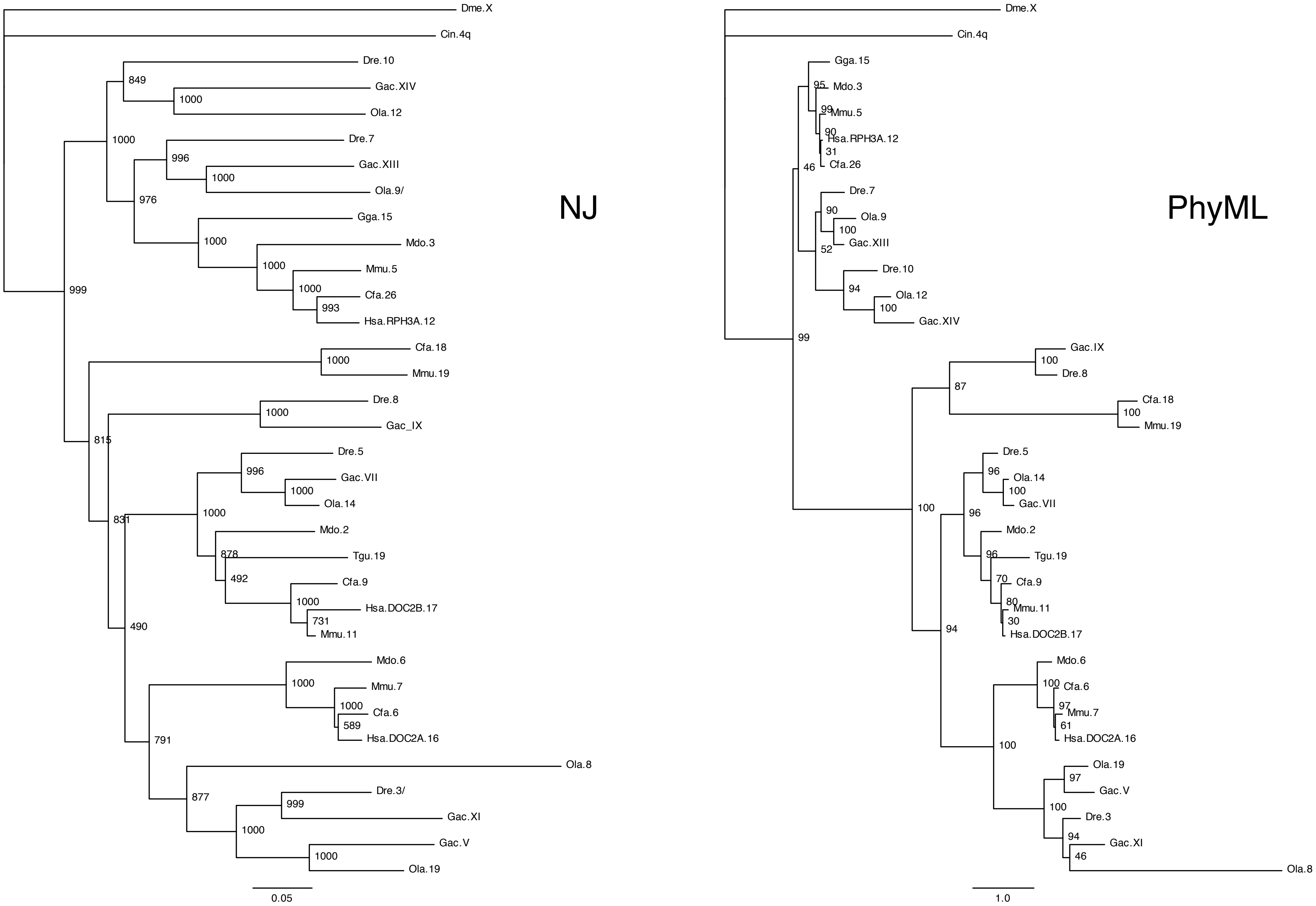

Figure S42. RPH3A

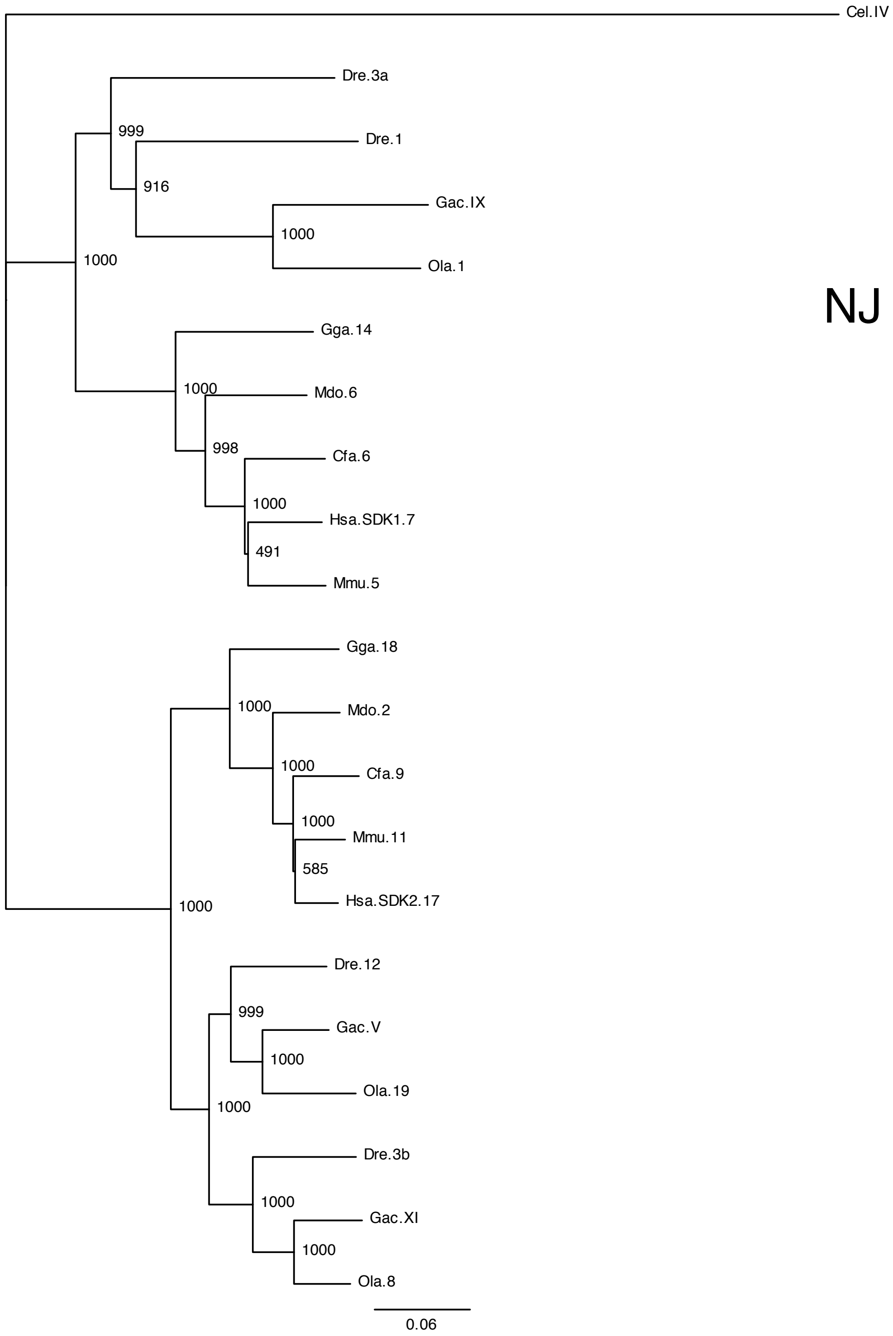

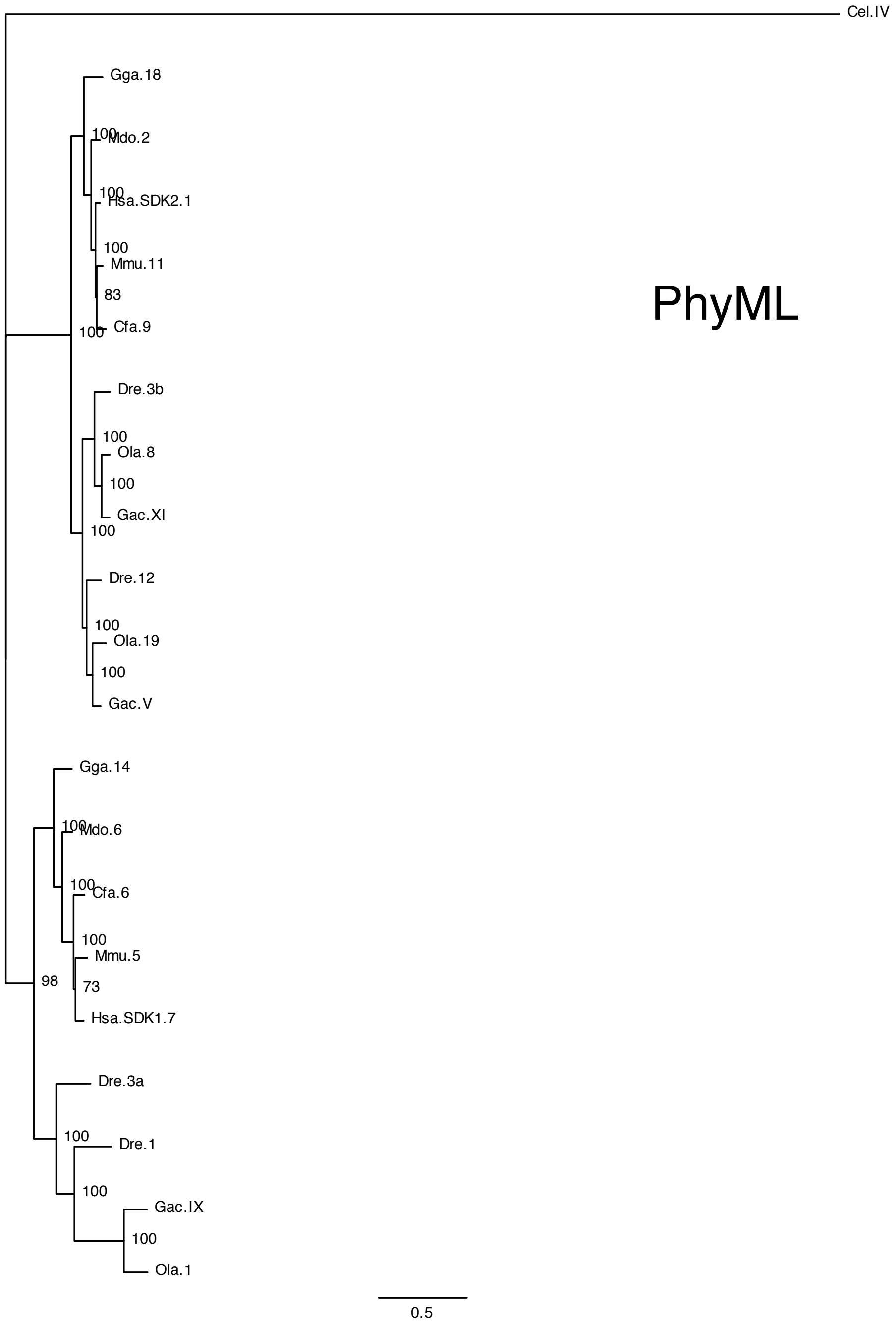

Figure S43. SDK

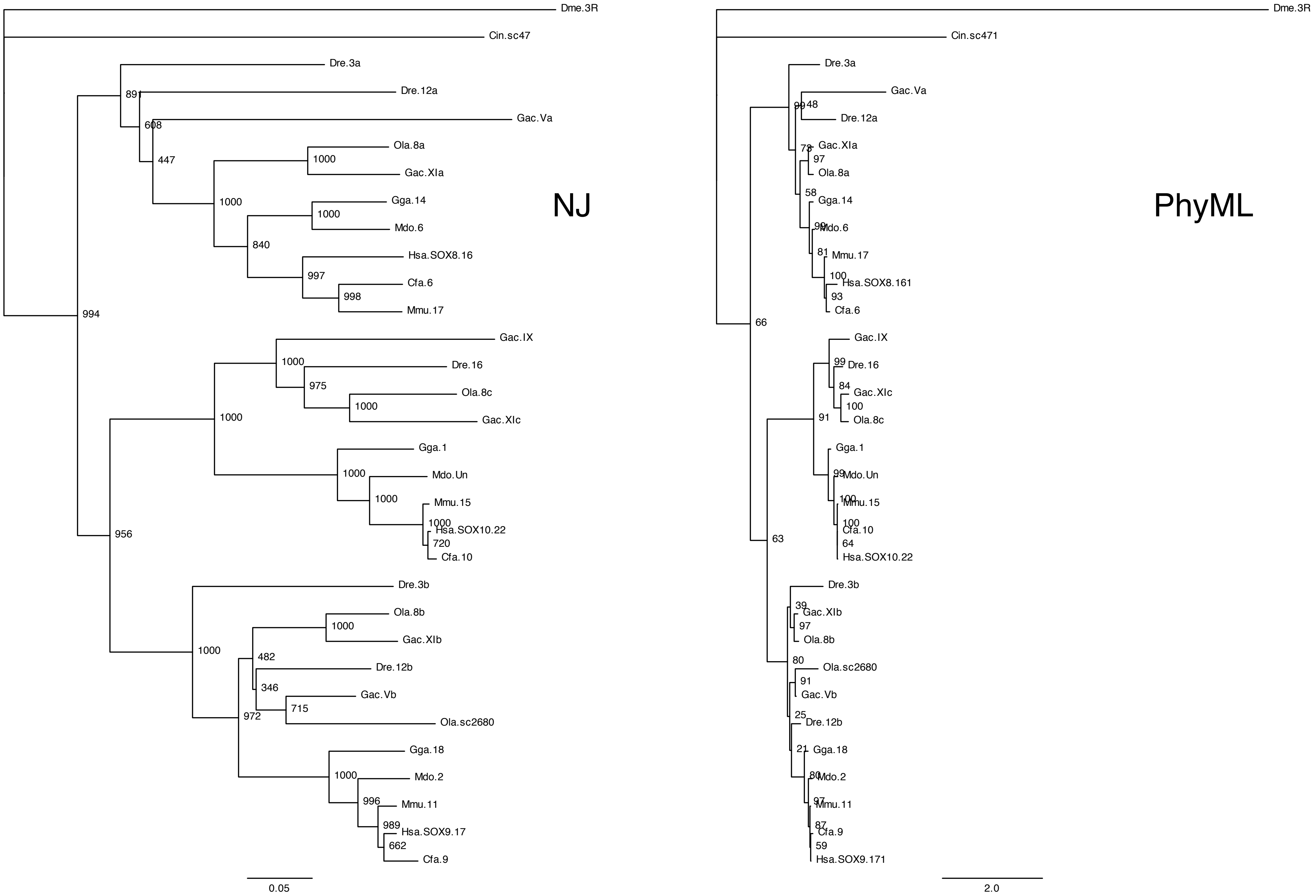

Figure S44. SOX

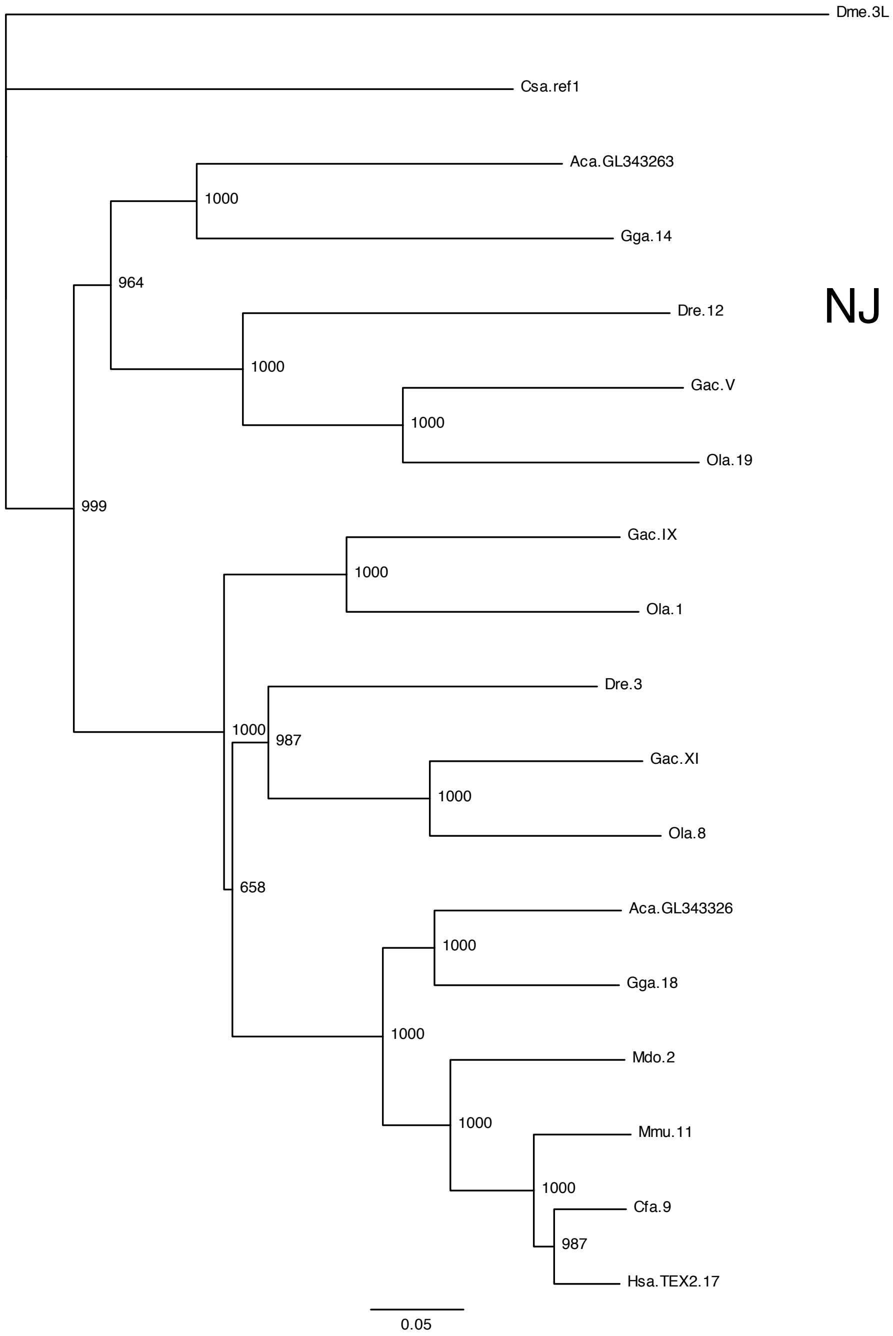

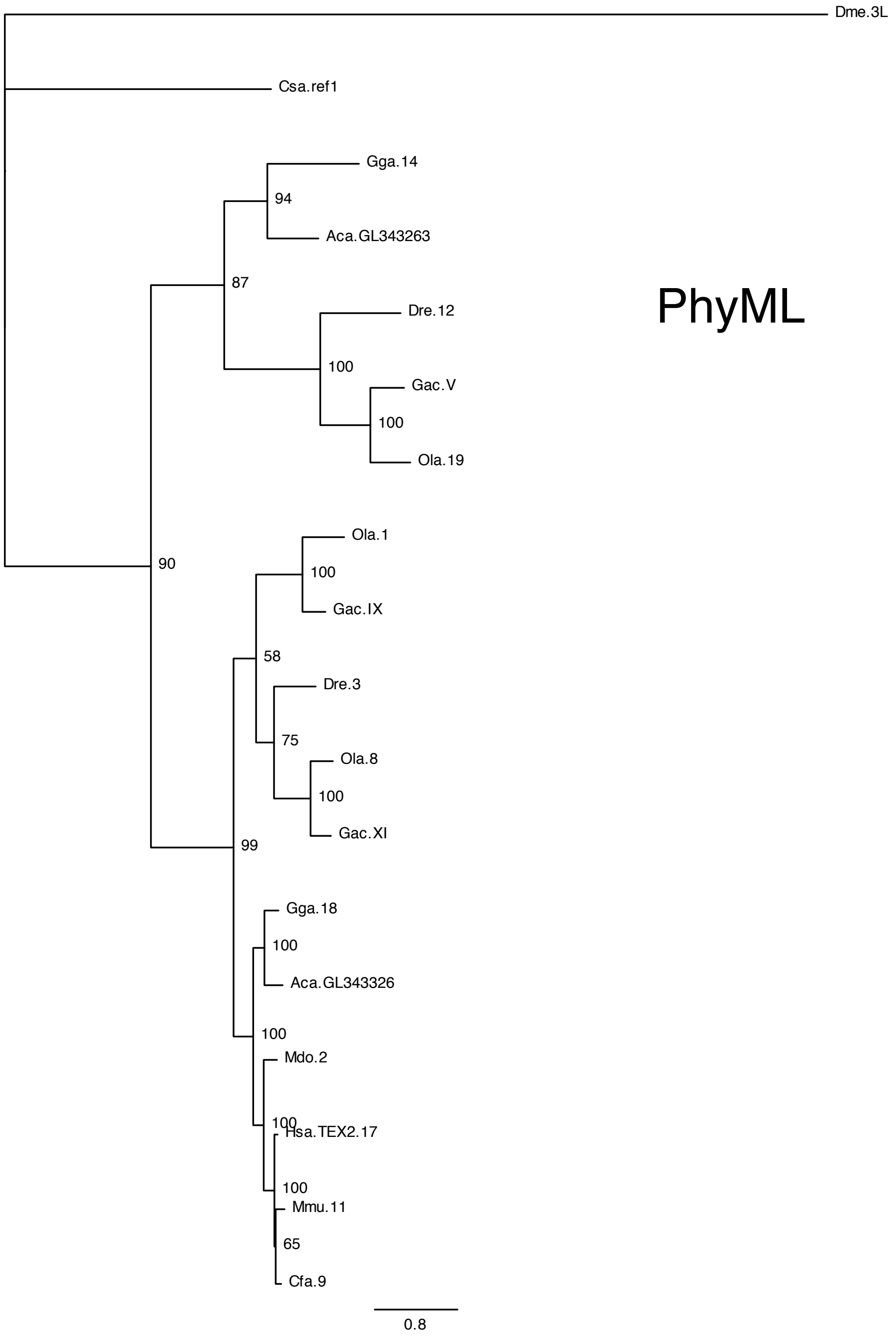

Figure S45. TEX2

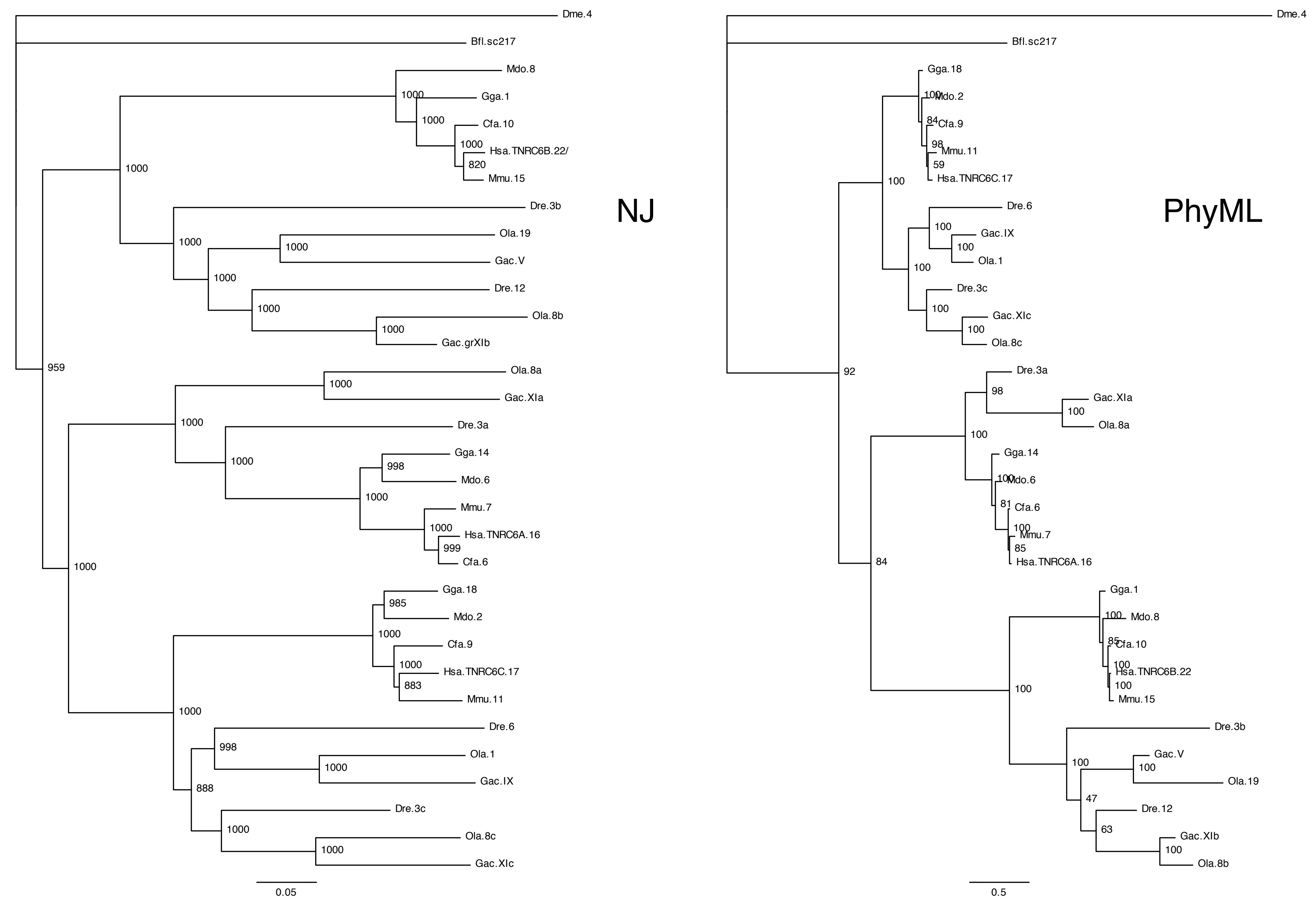

Figure S46. TNRC6

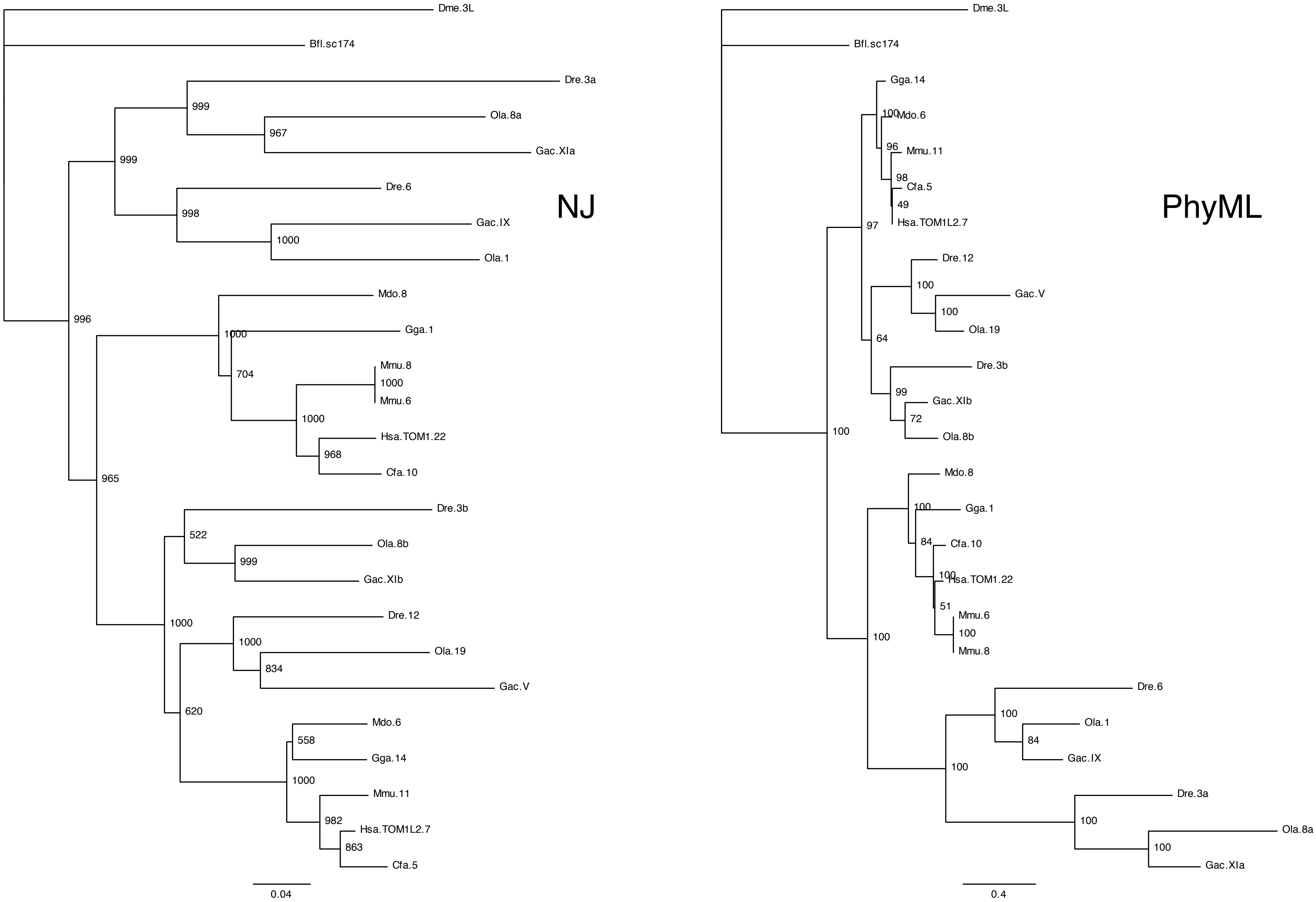

Figure S47. TOM1

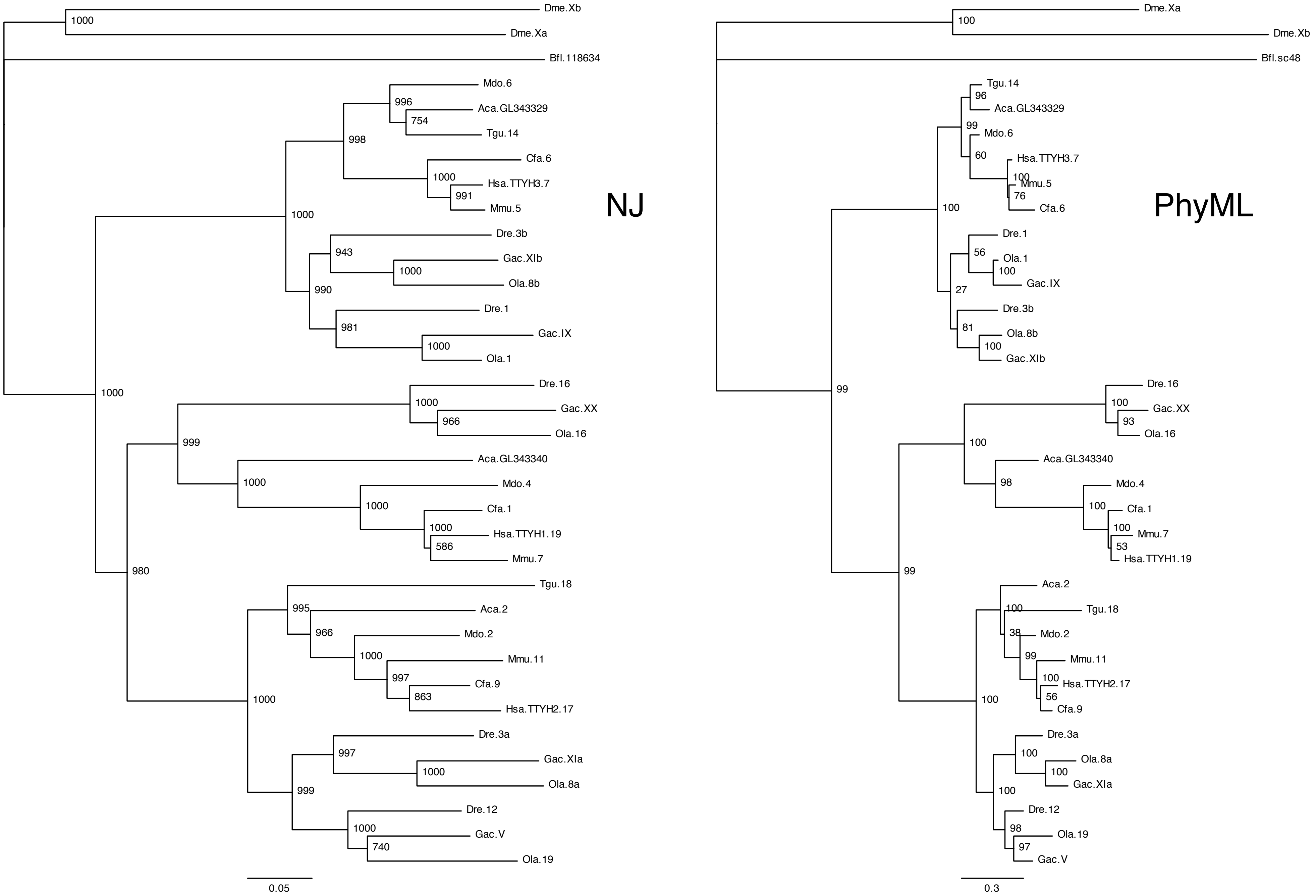

Figure S48. TTYH

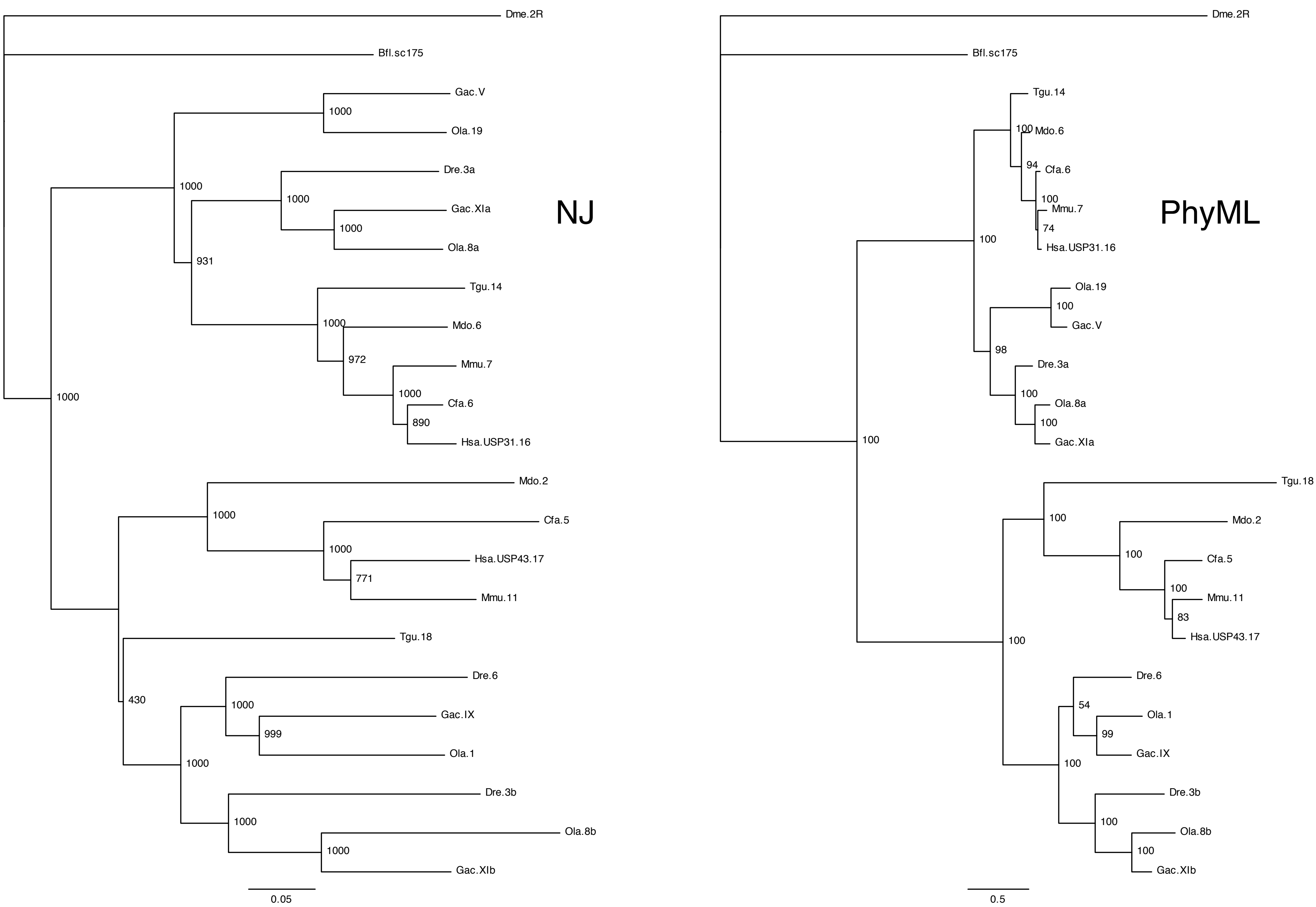

Figure S49. USP

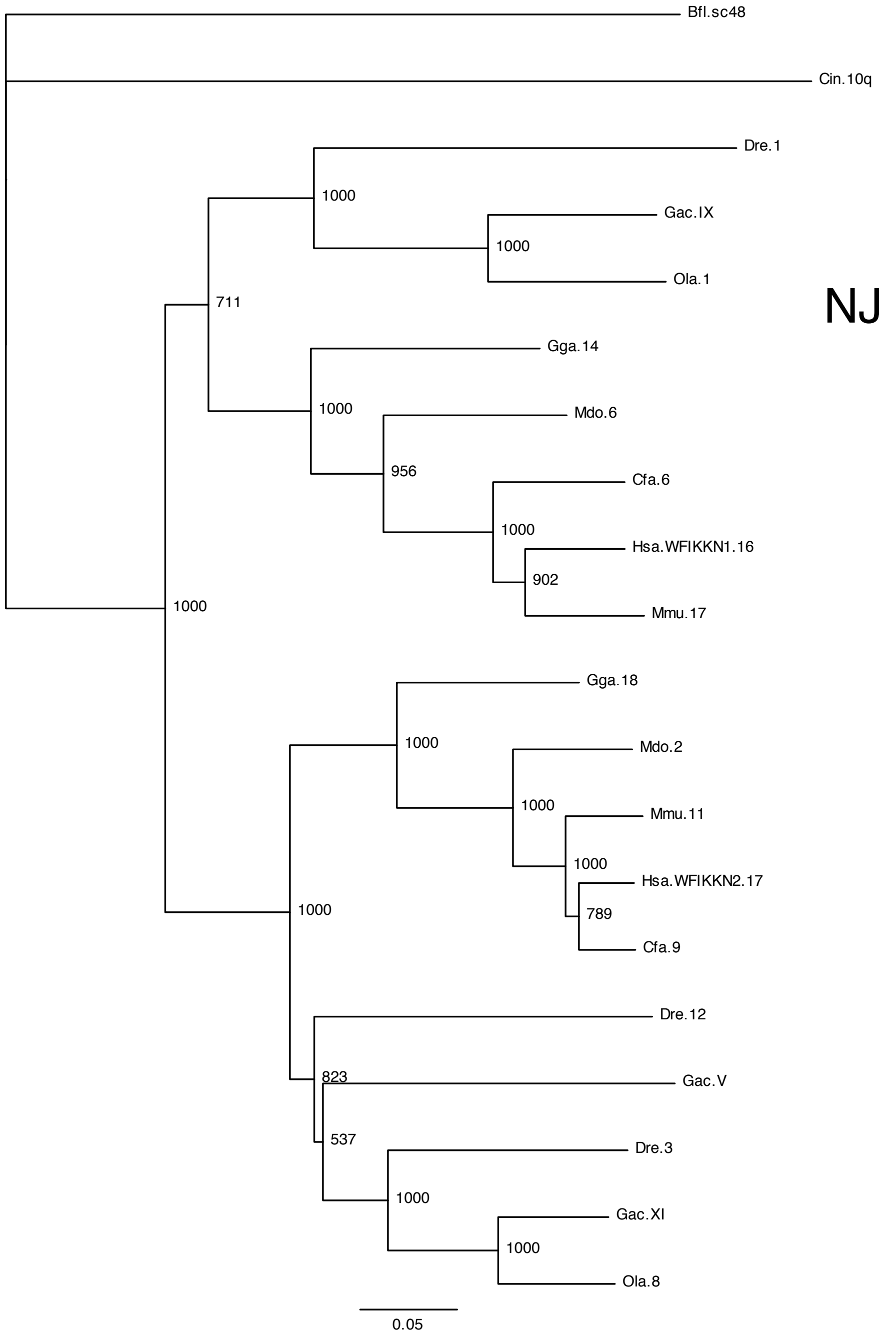

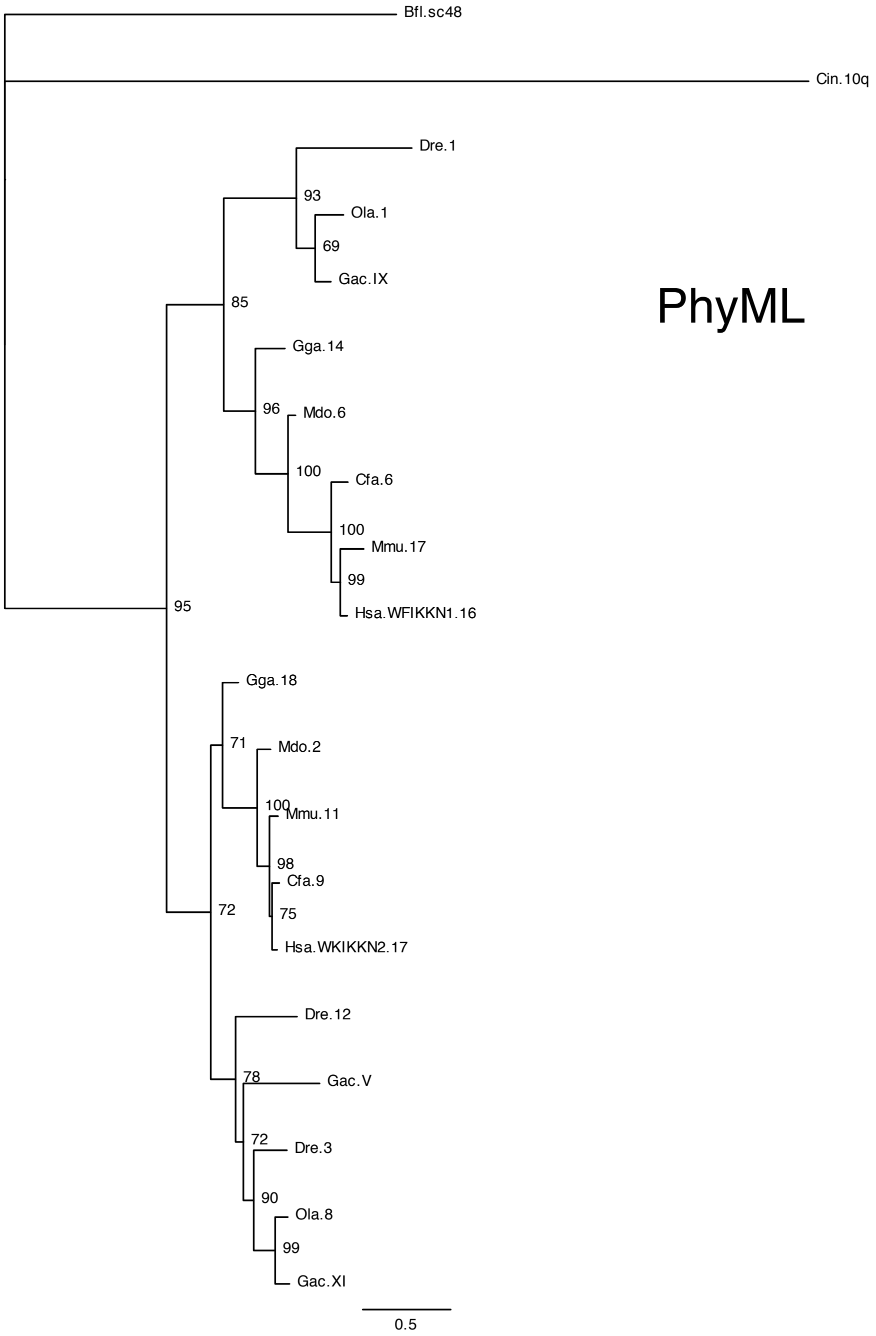

Figure S50. WFIKKN
